# A systematic review, meta-analysis *and* meta-regression amalgamating the driven approaches used *to* quantify dynamic cerebral autoregulation

**DOI:** 10.1177/0271678X241235878

**Published:** 2024-04-18

**Authors:** Joel S Burma, Marc-Antoine Roy, Courtney M Kennedy, Lawrence Labrecque, Patrice Brassard, Jonathan D Smirl

**Affiliations:** 1 Cerebrovascular Concussion Laboratory, Faculty of Kinesiology, 2129 University of Calgary, Calgary, Canada; 2 Sport Injury Prevention Research Centre, Faculty of Kinesiology, 2129 University of Calgary, Calgary, Canada; 3 Human Performance Laboratory, Faculty of Kinesiology, 2129 University of Calgary, Calgary, Canada; 4 Hotchkiss Brain Institute, University of Calgary, Calgary, Canada; 5 Integrated Concussion Research Program, University of Calgary, Calgary, Canada; 6 Alberta Children’s Hospital Research Institute, University of Calgary, Calgary, Canada; 7 Libin Cardiovascular Institute of Alberta, University of Calgary, Calgary, Canada; 8 Department of Kinesiology, Faculty of Medicine, Université Laval, Québec, Canada; 9 Research Center of the Institut Universitaire de Cardiologie et de Pneumologie de Québec, Québec, Canada

**Keywords:** Cerebral blood velocity, dynamic cerebral autoregulation, mean arterial pressure, transcranial Doppler ultrasound, transfer function analysis

## Abstract

Numerous driven techniques have been utilized to assess dynamic cerebral autoregulation (dCA) in healthy and clinical populations. The current review aimed to amalgamate this literature and provide recommendations to create greater standardization for future research. The PubMed database was searched with inclusion criteria consisting of original research articles using driven dCA assessments in humans. Risk of bias were completed using Scottish Intercollegiate Guidelines Network and Methodological Index for Non-Randomized Studies. Meta-analyses were conducted for coherence, phase, and gain metrics at 0.05 and 0.10 Hz using deep-breathing, oscillatory lower body negative pressure (OLBNP), sit-to-stand maneuvers, and squat-stand maneuvers. A total of 113 studies were included, with 40 of these incorporating clinical populations. A total of 4126 participants were identified, with younger adults (18–40 years) being the most studied population. The most common techniques were squat-stands (n = 43), deep-breathing (n = 25), OLBNP (n = 20), and sit-to-stands (n = 16). Pooled coherence point estimates were: OLBNP 0.70 (95%CI:0.59–0.82), sit-to-stands 0.87 (95%CI:0.79–0.95), and squat-stands 0.98 (95%CI:0.98–0.99) at 0.05 Hz; and deep-breathing 0.90 (95%CI:0.81–0.99); OLBNP 0.67 (95%CI:0.44–0.90); and squat-stands 0.99 (95%CI:0.99–0.99) at 0.10 Hz. This review summarizes clinical findings, discusses the pros/cons of the 11 unique driven techniques included, and provides recommendations for future investigations into the unique physiological intricacies of dCA.

## Introduction

Dynamic cerebral autoregulation (dCA) describes the ability of the cerebral vessels to respond to rapid changes in arterial blood pressure (ABP).^1,2^ The evaluation, quantification, and interpretation of dCA is not a simple mission considering researchers and clinicians have many options in terms of evaluation methods, analytical approaches, and metrics to examine this construct.^3^ One widespread technique to quantify dCA is transfer function analysis (TFA) on spontaneous and forced ABP and cerebral blood velocity (CBv) oscillations.^4,5^ The aim of TFA is to estimate variables reflecting the dynamic behavior of dCA, assuming that dCA acts as a linear control system.^4^ As a reminder, a system is termed “linear” if it has two mathematical properties: homogeneity (i.e. a change in the amplitude of the input signal leads to a matching change in the amplitude of the output signal) and additivity (i.e. any signals added at the input produce signals that are added at the output).^6^ The metrics resulting from TFA are coherence (fraction of the ABP which is linearly related to CBv), phase (difference in the timing of the ABP and CBv waveforms), and gain (amplitude of CBv change for a given change in ABP).^4,7^ This technique has demonstrated the cerebrovasculature acts as a high-pass filter, where oscillations slower than 0.20 Hz are dampened and oscillations above this threshold pass through unimpeded.^4,5,7^ However, regulation from <0.01 Hz to 0.20 Hz is not consistent, where slower oscillations in this range (e.g., 0.05 Hz compared to 0.10 Hz), will display lower gain values and higher phase values (i.e., greater regulation).^4,5,7^

Spontaneous ABP fluctuations have been commonly used for dCA quantification using TFA considering the natural variability in ABP usually comprises beat-to-beat changes, which are able to generate a dCA response.^4,5,7^ Spontaneous ABP oscillations are appealing for investigators who want to quantify dCA in clinical populations in whom it is not possible, or safe, to induce large transient ABP changes (e.g., acute stroke patients). This being acknowledged, the limited amplitude of these fluctuations (i.e., low signal-to-noise ratio) will generally lead to less reliable and reproducible estimations of dCA metrics when using TFA.^8,9^ In fact, dCA is a nonstationary phenomenon, meaning dCA is not constant over time, which in itself affects the reproducibility of dCA metrics calculated from spontaneous ABP fluctuations.^10^

One important assumption of TFA is the autoregulatory responses are linear, which is not the case, especially with spontaneous ABP oscillations.^3,10^ One strategy to increase the input (e.g., ABP) power and improve the linear interpretability of TFA metrics is by forcing larger transient fluctuations in ABP.^3^ This strategy provides changes in ABP, which are representative of physiologically and clinically pertinent challenges to dCA and increases our confidence there is a causal relationship between changes in ABP and CBv. Indeed, the dCA response may be undetectable with spontaneous ABP fluctuations because of other sources of physiological noise (e.g., respiration, vascular tone) in the signals.^11^ Over the years, investigators have utilized several techniques to induce large transient changes in ABP and CBv, such as repeated squat-stands maneuvers (usually at 0.05 and 0.10 Hz),^9,12–17^ repeated sit-stands,^18–20^ oscillatory lower body negative pressure (OLBNP),^8,21,22^ respiratory-induced oscillations,^23,24^ and passive leg raising.^25^

Previous work has demonstrated the repeated squat-stands maneuvers model to increase the input power and improve the linear interpretability and reproducibility of TFA metrics,^8,9^ however, limited information is available for the other techniques used to force ABP oscillations. Accordingly, the aims of this systematic review and meta-analysis are 1) to cover the pros and cons associated with the various methods currently employed to enhance TFA coherence and improve the signal-to-noise ratio (i.e., repeated squat-stands maneuvers, OLBNP, deep-breathing, passive leg raises, sit-to-stands maneuvers, rhythmic handgrip contractions, head-up tilt; thigh cuffs, cold pressor test, Valsalva maneuver); 2) to derive pooled TFA estimates for coherence, phase, gain, and normalized gain values from the commonly used driven techniques; 3) to summarize the clinical studies, which have used forced ABP oscillations to identify the feasibility and safety of driven techniques compared to spontaneous measures; 4) to provide future directions to augment standardization within the dCA research realm; and 5) to describe the general limitations of the included studies and the current state of the field.

## Methods

The current systematic review conformed with all recommendations and standards put forth within the Preferred Reporting Items for Systematic Reviews and Meta-Analysis guidelines.^26,27^ The search strategy and inclusion and exclusion criteria were developed by JBS, PB, and JDS for the PubMed database, as this database indexes the near majority of original investigations into the cerebral pressure-flow relationship. With the aid of a librarian, the search terms and strategy were refined, ensuring several seed articles were included in the final search strategy. Articles were compiled in Endnote (Philadelphia, PA, United States) to remove duplicates and manage all references.

Inclusion criteria consisted of studies that assess the cerebral pressure-flow relationship using driven approaches. These were defined as methods employed to cause ABP oscillations for a minimum of two minutes, where either TFA or other analytical approaches could be performed at the frequency the oscillations were driven. Previous work has demonstrated a minimum of ∼4-minutes are required to elicit valid and reliable TFA estimates during squat-stand manvuers;^28^ however, the two-minute threshold was used as a conservative approach to ensure all driven articles were included. Recording length was nonetheless assessed during the study quality phase (i.e., Risk of Bias). Additional eligibility criteria included: the sample population being human participants, transcranial Doppler ultrasound was used to assess the cerebral pressure-flow relationship, written in English, had to have a sample size with five or more participants, which ensured smaller validation studies were included and discussed based on the merit a novel approach may have, original research articles (randomized control trials, cross-sectional studies, cohort studies, or quasi-experimental designs), and articles published between January 1st, 1982 and November 1st, 2023. This time frame was used as the first paper to use transcranial Doppler ultrasound to assess velocity within the intracranial arteries was by Aaslid and colleagues in 1982.^29^ Finally, blood pressure had to be obtained through either an invasive arterial catheter or a non-invasive finger photoplethysmography (e.g., Finapres) with the ability to detect peak-systolic and end-diastolic pressure on a beat to beat basis.^30^

Rapid, title and abstract, and full-text screening took place within the Rayyan platform (https://rayyan.ai/). The rapid screening was completed by JSB to remove non-original articles (e.g., reviews, theses, case reports, etc.), those not written in English, and studies conducted in animal models. Two independent and blinded reviewers completed the title and abstract and full-text screening, which was completed by a combination of JSB, MAR, CMK, and LL. Discrepancies were resolved by JSB. To ensure all eligible articles were included, reference lists from included articles were scanned and double checked to identify any articles that may have been missed by the search strategy.

From the studies satisfying inclusion criteria, the following data were extracted: title, authors, published year, location of study, sex of participants, age of participants, number of participants, study inclusion and exclusion criteria, vessel insonated, driven technique used, frequencies of ABP oscillations, concurrent neuroimaging modality used, outcome metrics used to quantify dCA, clinical population included, sample size calculation, statistical analyses, results, and key findings from the studies. Age from the participants involved in the included studies were stratified into groupings of children (5–12 years), adolescent (13–17 years), young adulthood (18–39 years), middle adulthood (40–64 years), and/or older adulthood (65+ years) based on the mean/median and deviation of the included age within each given study. Data extraction was completed by JSB, MAR, CMK, LL, and PB.

Risk of bias was completed on included studies using a modified Scottish Intercollegiate Guideline Network (SIGN)^31^ and the Methodological Index for Non-Randomized Studies (MINORS).^32^ The former categorizes studies into high risk of bias (inadmissible), moderate risk of bias (acceptable), and low risk of bias (high-quality). The risk of bias refers to each studies ability to control for potential confounding influences that may influence the outcome measures. For example, a study that considered the menstrual cycle/hormones for female participants, assessed cardiorespiratory fitness, and controlled for breathing patterns during data collection, would be rated as lower risk of bias (i.e., high quality) given it controlled for several physiologically confounding variables. Moreover, elevations and reductions in carbon dioxide levels are known to result in 3–6%/Torr elevations and 1–3%/Torr reductions in CBv, respectively.^33^ Therefore, if a driven method was used that involved respiratory challenges/changes, a study was deemed high risk of bias (i.e., low quality) if it did not report carbon dioxide values across the task and/or controlled for this within the statistical analysis. The MINORS checklist consists of 12 items ranging from 0 to 2, categorized as an item not being reported (0), reported but inadequate (1), and reported and adequate (2).^32^ Articles were randomized between authors where two independent, blinded reviews were completed JSB, MAR, CMK, LL, and PB.). To minimize conflicts of interest, authors from the current author group were not allowed to complete risk of bias and/or data extraction on articles they authored. JSB settled any discrepancies.

Meta-analyses were planned *a priori* to be completed on dCA outcome metrics that had several articles with sufficient homogeneity regarding the driven technique utilized, point-estimates of interest, and outcome metrics (e.g., deep breathing at 0.10 Hz using TFA, OLBNP at 0.05 Hz using project pursuit regression.^27^ Upon completion of data extraction, the most common approach used was TFA under eucapnic conditions, with a lesser degree of articles completing directional sensitivity, projection pursuit regression, or another analyses. However, these latter lacked sufficient articles across research groups to provide meaningful pooled estimates. Therefore, meta-analyses were performed on TFA metrics including mean arterial pressure (MAP) power spectral density (PSD) (mmHg^2^/Hz), middle cerebral artery (MCA) and posterior cerebral artery (PCA) PSD ((cm/s)^2^/Hz), coherence, phase (radians), gain (cm/s/mmHg), and normalized gain (%/mmHg).^7^ While only two studies are required to produce meaningful pooled estimates for a meta-analysis, this only holds true if there is sufficient homogeneity between studies. Therefore, a conservative approach was employed in the current investigation, where meta-analysis was employed if three or more studies completed the same driven technique at the same point-estimate of interest. Inverse variance meta-analyses with Sidik and Jonkman method for random effects,^34^ were used to compute weighted mean difference pooled estimates and the associated 95% confidence intervals (95%CI) based on a null value of zero (i.e., comparator). The Sidik and Jonkman method was used based on its favorable error rate, especially with a limited number of studies included in a meta-analysis.^34^ To quantify study heterogeneity, a complimentary approach of the Q-statistic p-value (Q-pval) and I^2^ statistic was used, where the former determines if the observed effect size variation is significant beyond chance alone, while the latter determines the magnitude of heterogeneity.^35^ Thresholds of heterogeneity from the I^2^ statistic were classified as low (0.00–0.25), moderate (0.25–0.50), and high (0.50–1.00).^35^ To quantify the magnitude difference in TFA outcome metrics between driven approaches for the accumulation of studies, Wilcoxon *r* effect sizes were computed using thresholds of 0.00–0.30 (small), 0.30–0.50 (moderate), and 0.50–1.00 (large). To better understand potential covariates of interest that may explain some of the variation between studies, meta-regression was used for TFA estimates produced from squat-stand maneuvers given these meta-analysis contained a minimum of 23 groupings.^36^ For the linear regressions, sex (female only [reference group], male only, and mixed), age (continuous), and sample size (continuous) were included as predictor variables that may influence the TFA squat-stands maneuvers outcome variables. Alpha was set a-priori at 0.05.

Of the five *a priori* aims, the meta-analyses and meta-regression assessed the robustness of various driven techniques and created pooled estimates. A qualitative narrative was used for the remaining four *a priori* aims describing the pros and cons of each driven technique, the use of these techniques in various clinical populations, suggestions for future research based on the current findings, and the common limitations identified in the included studies during the risk of bias screening.

## Results

### Screening and risk of bias

**Figure 1.**
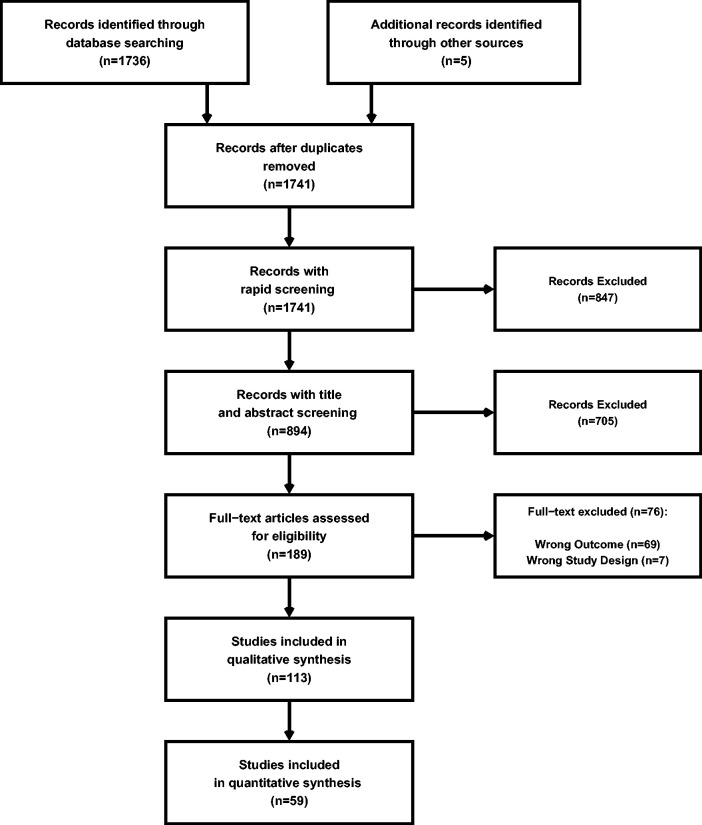
A PRISMA diagram denoting the number of studies screened and included in both the qualitative and quantitative syntheses.

A total of 1671 articles were assessed for eligibility with 185 being screened for eligibility at the full-text stage (Figure 1). Qualitative synthesis was conducted on the 113 articles that met all eligibility criteria with 59 of these additionally being utilized in the meta-analyses of TFA estimates (i.e., quantitative synthesis) (Figure 1). Of the included articles (Figure 1), 19.5% (n = 22) were deemed high risk of bias, 51.3% (n = 58) were deemed moderate risk of bias, and 29.2% (n = 33) were deemed low risk of bias assessed through the SIGN checklists (Table 1). These are further stratified by driven technique in Table 1 with Supplemental A presenting the full bibliography of included articles. The individual SIGN and MINORS ratings for all studies are displayed in Supplemental B.

**Table 1.** Risk of bias assessments on included articles using the Scottish Intercollegiate Guideline Network checklists. This categorizes articles into high risk of bias (inadmissible), moderate risk of bias (acceptable), and low risk of bias (high-quality).

Driven technique	High quality	Acceptable	Inadmissible	Total studies
*Cyclical Pump Flow*	0 (0%)	0 (0%)	1 (100%)	1
*Deep Breathing*	1 (4%)	12 (48%)	12 (48%)	25
*Head Up Tilt*	0 (0%)	0 (0%)	2 (100%)	2
*Leg Cuff*	0 (0%)	0 (0%)	2 (100%)	2
*Leg Raises*	0 (0%)	1 (100%)	0 (0%)	1
*Neck Suction*	0 (0%)	2 (66.7%)	1 (33.3%)	3
*Oscillatory LBNP*	6 (30%)	12 (60%)	2 (10%)	20
*Rhythmic Handgrip*	0 (0%)	0 (0%)	1 (100%)	1
*Sit to Stand*	3 (18.8%)	12 (75%)	1 (6.3%)	16
*Squat-Stand Maneuvers*	24 (55.8%)	18 (41.9%)	1 (2.3%)	43
*Ventilated Breathing*	0 (0%)	1 (100%)	0 (0%)	1
Total	33 (29.2%)	58 (51.3%)	22 (19.5%)	113

LBNP: lower body negative pressure.

### Study demographics and characteristics

**Figure 2.**
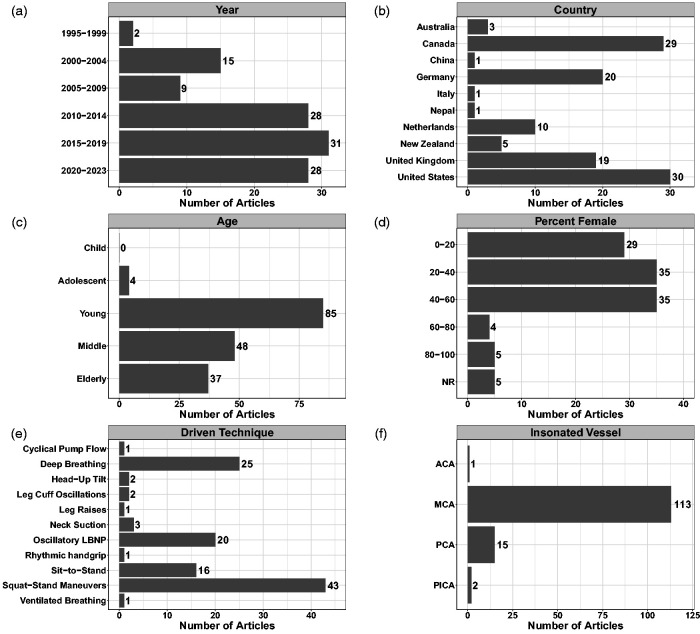
Demographics and characteristics of the included studies with respect to (a) year of publication, (b) country of data collection, (c) age of participants, (d) percentage of female participants included, (e) driven technique used, and (f) vessel(s) insonated. The sum for panels b, c, e, and f exceed the total number of studies included in the review, as some investigations were conducted in multiple countries, with more than one driven technique, included multiple age groupings, and/or insonated more than one vessel. Sex was not reported (NR) in five studies. Anterior cerebral artery (ACA); middle cerebral artery (MCA); posterior cerebral artery (PCA), and posterior inferior cerebral artery (PICA).

Figure 2 highlights there were two publications related to that topic before 2000, with an increasing number of articles with each successive decade. Over half of these articles were completed in North America (52.2%) and the vast majority had data collection within either North America or Europe (94.7%) (Figure 2). Younger participants were the most studied individuals with only four (3.5%) of the included studies involving adolescent participants (Figure 2). No investigations were completed in children (Figure 2). The average proportion of females included across studies was 35% (median: 34%, interquartile range: 13.8–50.0%) (Figure 2). The most common techniques included squat-stands maneuvers (n = 43; 38.1%), deep-breathing (n = 25; 22.1%), OLBNP (n = 20; 17.7%), and sit-to-stand maneuvers (n = 16; 14.2%), while the remaining techniques were used in three or fewer studies (Figure 2). The middle cerebral artery (MCA) was insonated in every study, with a handful also including the posterior cerebral artery (PCA) (n = 15; 13.2%) (Figure 2). Two studies insonated the posterior inferior cerebellar artery (1.8%) and one insonated the anterior cerebral artery (0.9%) (Figure 2). Supplemental C displays the study characteristics and demographics for each individual study, while Supplemental D denotes the studies that have been completed in clinical populations (n = 40).

### Meta-Analysis pooled estimates and effect size differences

**Figure 3.**
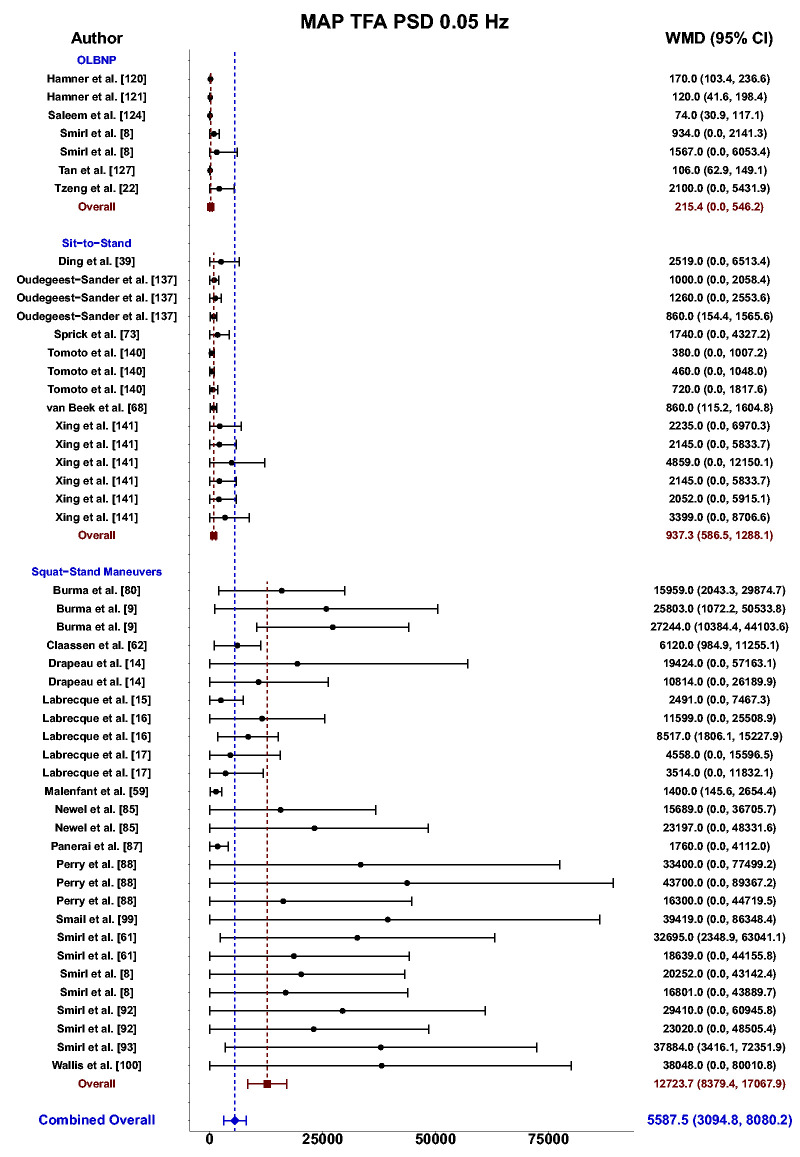
Forest plot depicting the weighted mean difference (WMD) and 95% confidence intervals (CI) for mean arterial pressure (MAP) transfer function analysis (TFA) power spectral density (PSD) values at 0.05 Hz using oscillatory lower body negative pressure (OLBNP), sit-to-stand maneuvers, and squat-stand maneuvers. The pooled estimates from all studies were also computed across all included driven techniques.

**Figure 4. fig4-0271678X241235878:**
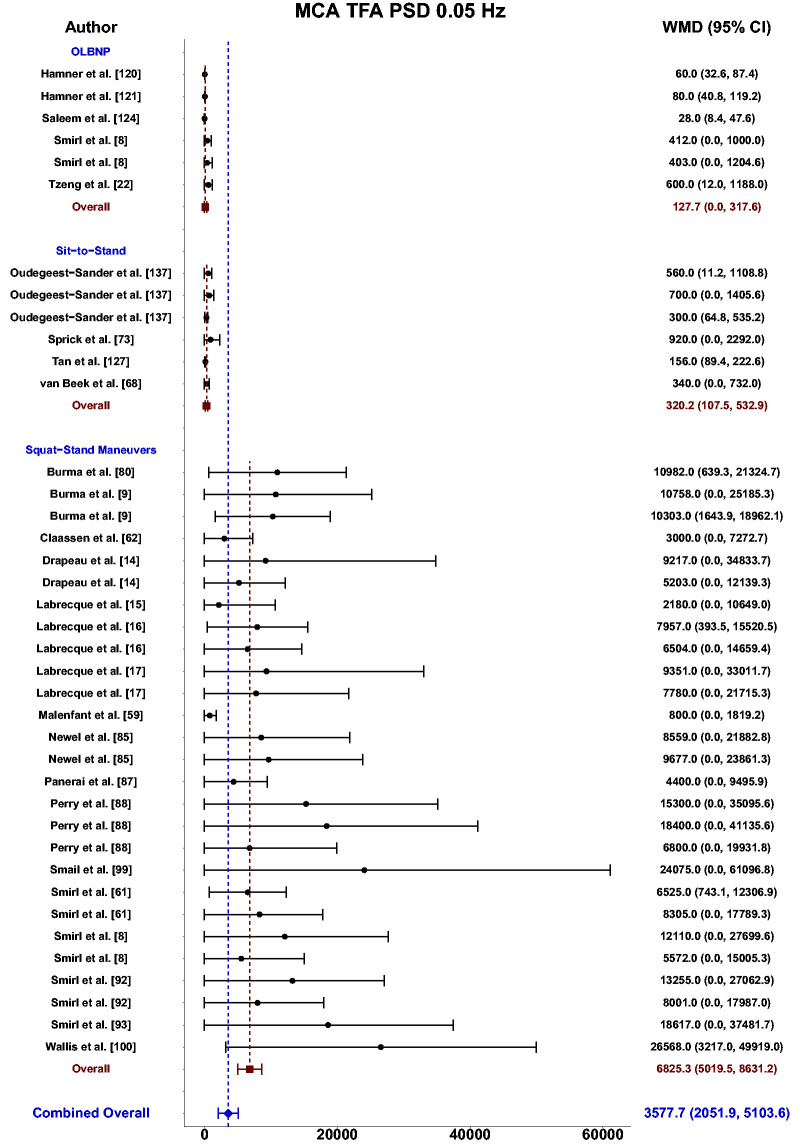
Forest plot depicting the weighted mean difference (WMD) and 95% confidence intervals (CI) for middle cerebral artery (MCA) transfer function analysis (TFA) power spectral density (PSD) values at 0.05 Hz using oscillatory lower body negative pressure (OLBNP), sit-to-stand maneuvers, and squat-stand maneuvers. The pooled estimates from all studies were also computed across all included driven techniques.

**Figure 5. fig5-0271678X241235878:**
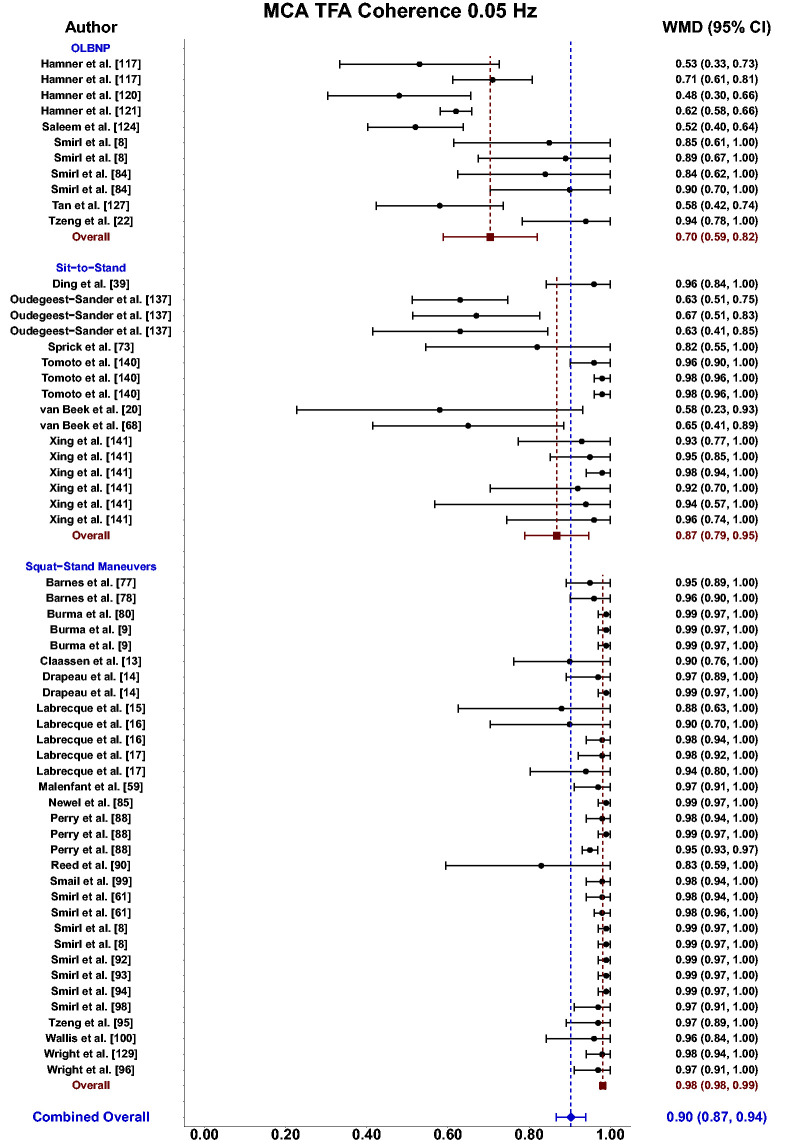
Forest plot depicting the weighted mean difference (WMD) and 95% confidence intervals (CI) for transfer function analysis (TFA) coherence values derived in the middle cerebral artery (MCA) at 0.05 Hz using oscillatory lower body negative pressure (OLBNP), sit-to-stand maneuvers, and squat-stand maneuvers. The pooled estimates from all studies were also computed across all included driven techniques.

Meta-analysis revealed the pooled MAP PSD estimates at 0.05 Hz was 215 mmHg^2^/Hz (95%CI: 0–546; Q-pval = 0.154; I^2^ = 0.36; n = 7) for OLBNP, 937 mmHg^2^/Hz (95%CI: 586–1288; Q-pval = 0.852; I^2^ = 0.00; n = 15) for sit-to-stand maneuvers, and 12724 mmHg^2^/Hz (95%CI: 8379–17068; Q-pval < 0.001; I^2^ = 0.57; n = 27) for squat-stands maneuvers (Table 2 and Figure 3). These translated to 0.05 Hz MCA PSD estimates of 128 cm/s^2^/Hz (95%CI: 0–318; Q-pval = 0.025; I^2^ = 0.61; n = 6) for OLBNP, 320 cm/s^2^/Hz (95%CI: 108–533; Q-pval = 0.208; I^2^ = 0.30; n = 6) for sit-to-stand maneuvers, and 6825 cm/s^2^/Hz (95%CI: 5019–8631; Q-pval < 0.001; I^2^ = 0.64; n = 27) for squat-stands maneuvers (Table 2 and Figure 4). Pooled estimates of MCA coherence at 0.05 Hz was 0.70 (95%CI: 0.59–0.82; Q-pval < 0.001; I^2^ = 0.77; n = 11) for OLBNP, 0.87 (95%CI: 0.79–0.95; Q-pval < 0.001; I^2^ = 0.79; n = 16) for sit-to-stand maneuvers, and 0.98 (95%CI: 0.98–0.99; Q-pval = 0.823; I^2^ = 0.00; n = 32) for squat-stands maneuvers (Table 2 and Figure 5). Supplemental E demonstrates greater values were obtained when comparing squat-stands to both OLBNP and sit-to-stands at 0.05 Hz for MAP PSD, MCA PSD, and coherence (Wilcoxon *r* range: 0.54–0.71 [large]). Sit-to-stands elicited higher MAP PSD, MCA PSD, and coherence values at 0.05 Hz compared to OLBNP (Wilcoxon *r* range: 0.37–0.47 [moderate] (Supplemental E).

**Table 2.** Meta-analysis with pooled estimates (95% confidence intervals) for transfer function analysis metrics at driven frequencies where there were 3 or more included studies for each respective methodological approach. Heterogeneity between studies were quantified with the I^2^ statistic, with a higher percent representing more heterogeneity. However, it is important to note a high I^2^ estimate is not always synonymous with physiological relevant heterogeneity.

	Deep-breathing	Oscillatory lower body negative pressure	Sit-to-stand maneuvers	Squat-stand maneuvers
*Power spectral densities (mmHg^2^/Hz or cm/s^2^/Hz)*				
MAP 0.05 Hz	–	215 (0, 546)I² = 0.36, p = 0.154, N = 7, n = 75	937 (586, 1288) I² = 0.00, p = 0.852, N = 15, n = 474	12724 (8379, 17068) I² = 0.57, p < 0.001, N = 27, n = 333
MAP 0.10 Hz	64 (0, 207) I² = 0.86, p = 0.001, N = 3, n = 38	634 (386, 882) I² = 0.00, p = 0.968, N = 4, n = 35	–	11206 (7892, 14519) I² = 0.63, p < 0.001, N = 26, n = 301
MCA 0.05 Hz	–	128 (0, 318) I² = 0.61, p = 0.025, N = 6, n = 59	320 (108, 533) I² = 0.30, p = 0.208, N = 6, n = 114	6825 (5019, 8631) I² = 0.42, p = 0.012, N = 27, n = 333
MCA 0.10 Hz	17 (0, 47) I² = 0.00, p = 0.566, N = 3, n = 38	359 (64, 654) I² = 0.00, p = 0.806, N = 4, n = 35	–	9612 (6585, 12638) I² = 0.64, p < 0.001, N = 26, n = 301
PCA 0.05 Hz	–	–	–	3531 (2820, 4243) I² = 0.00, p = 0.992, N = 5, n = 50
PCA 0.10 Hz	–	–	–	4601 (2804, 6397)I² = 0.00, p = 0.943, N = 5, n = 50
*Coherence (no units)*				
MCA 0.03 Hz	–	0.49 (0.38, 0.61) I² = 0.81, p < 0.001, N = 6, n = 72	–	–
MCA 0.05 Hz	–	0.70 (0.59, 0.82) I² = 0.77, p < 0.001, N = 11, n = 118	0.87 (0.79, 0.95) I² = 0.79, p < 0.001, N = 16, n = 455	0.98 (0.98, 0.99)I² = 0.00, p = 0.823, N = 32, n = 641
MCA 0.07 Hz	–	0.66 (0.36, 0.96) I² = 0.92, p < 0.001, N = 4, n = 54	–	–
MCA 0.10 Hz	0.83 (0.61, 1.06) I² = 0.49, p = 0.097, N = 5, n = 63	0.90 (0.81, 0.99) I² = 0.83, p < 0.001, N = 7, n = 64	0.67 (0.44, 0.90) I² = 0.00, p = 0.597, N = 3, n = 67	0.99 (0.99, 0.99) I² = 0.00, p = 0.999, N = 32, n = 629
PCA 0.05 Hz	–	–	–	0.99 (0.98, 0.99)I² = 0.00, p = 0.993, N = 9, n = 106
PCA 0.10 Hz	–	–	–	0.99 (0.99, 0.99)I² = 0.00, p > 0.999, N = 8, n = 93
*Phase (radians)*				
MCA 0.03 Hz	–	0.75 (0.66, 0.84) I² = 0.00, p = 0.882, N = 3, n = 36	–	–
MCA 0.05 Hz	–	0.92 (0.65, 1.19) I² = 0.76, p < 0.001, N = 8, n = 82	0.81 (0.73, 0.88) I² = 0.00, p = 0.996, N = 16, n = 455	0.74 (0.66, 0.82)I² = 0.50, p = 0.001, N = 32, n = 634
MCA 0.10 Hz	0.88 (0.61, 1.14) I² = 0.35, p = 0.111, N = 12, n = 283	0.55 (0.50, 0.61) I² = 0.00, p = 0.967, N = 7, n = 64	0.91 (0, 1.96) I² = 0.70, p = 0.037, N = 3, n = 67	0.48 (0.40, 0.56)I² = 0.78, p < 0.001, N = 33, n = 643
PCA 0.05 Hz	–	–	–	0.70 (0.58, 0.82)I² = 0.00, p = 0.584, N = 8, n = 93
PCA 0.10 Hz	0.74 (0.56, 0.93) I² = 0.00, p = 0.892, N = 5, n = 122	–	–	0.52 (0.25, 0.79)I² = 0.87, p < 0.001, N = 8, n = 93
*Gain (cm/s/mmHg)*				
MCA 0.03 Hz	–	0.39 (0.28, 0.50) I² = 0.65, p = 0.015, N = 6, n = 72	–	–
MCA 0.05 Hz	–	0.57 (0.48, 0.67) I² = 0.72, p < 0.001, N = 11, n = 118	0.60 (0.51, 0.69) I² = 0.00, p = 0.954, N = 6, n = 127	0.67 (0.64, 0.71)I² = 0.00, p > 0.999, N = 26, n = 503
MCA 0.07 Hz	–	0.58 (0.37, 0.79) I² = 0.84, p < 0.001, N = 4, n = 54	–	–
MCA 0.10 Hz	0.98 (0.66, 1.31) I² = 0.32, p = 0.185, N = 7, n = 100	1.03 (0.83, 1.23) I² = 0.45, p = 0.091, N = 7, n = 64	0.63 (0.42, 0.85) I² = 0.00, p = 0.677, N = 3, n = 67	0.87 (0.82, 0.92)I² = 0.00, p = 0.933, N = 26, n = 498
PCA 0.05 Hz	–	–	–	0.41 (0.36, 0.47)I² = 0.00, p = 0.941, N = 8, n = 93
PCA 0.10 Hz	–	–	–	0.56 (0.49, 0.64)I² = 0.00, p = 0.908, N = 8, n = 93
*Normalized gain (%/mmHg)*				
MCA 0.05 Hz	–	–	1.40 (1.20, 1.60) I² = 0.02, p = 0.423, N = 14, n = 408	1.06 (1.03, 1.10)I² = 0.00, p > 0.999, N = 23, n = 448
MCA 0.10 Hz	0.87 (0.72, 1.02) I² = 0.00, p = 0.858, N = 4, n = 130	–	–	1.39 (1.33, 1.46)I² = 0.00, p = 0.994, N = 24, n = 472
PCA 0.05 Hz	–	–	–	1.07 (0.97, 1.17)I² = 0.00, p = 0.964, N = 7, n = 77
PCA 0.10 Hz	0.79 (0.59, 0.99) I² = 0.00, p = 0.808, N = 3, n = 74	–	–	1.45 (1.36, 1.54)I² = 0.00, p = 0.976, N = 7, n = 77

N represents the number of studies included and n represents the number of individuals included. nGain: normalized gain; MCA: middle cerebral artery; PCA: posterior cerebral artery.

**Figure 6. fig6-0271678X241235878:**
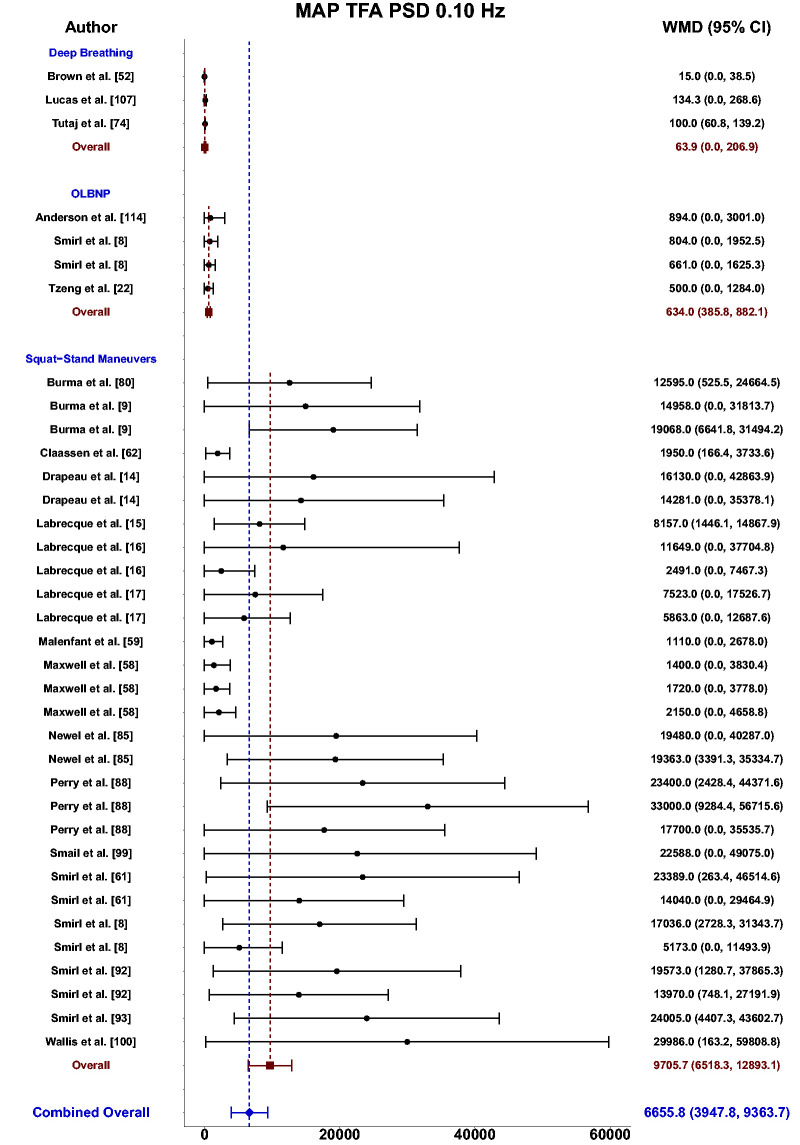
Forest plot depicting the weighted mean difference (WMD) and 95% confidence intervals (CI) for mean arterial pressure (MAP) transfer function analysis (TFA) power spectral density (PSD) values at 0.10 Hz using deep breathing, oscillatory lower body negative pressure (OLBNP), and squat-stand maneuvers. The pooled estimates from all studies were also computed across all included driven techniques.

**Figure 7. fig7-0271678X241235878:**
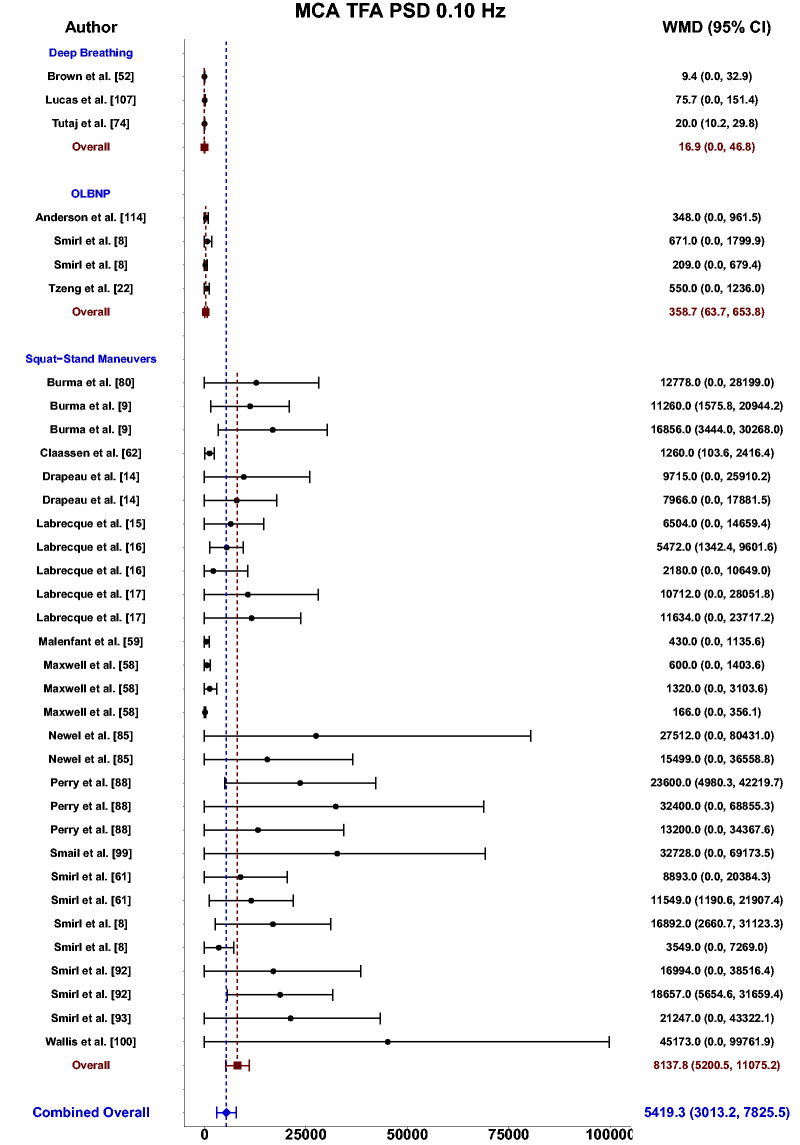
Forest plot depicting the weighted mean difference (WMD) and 95% confidence intervals (CI) for middle cerebral artery (MCA) transfer function analysis (TFA) power spectral density (PSD) values at 0.10 Hz deep breathing, oscillatory lower body negative pressure (OLBNP), and squat-stand maneuvers. The pooled estimates from all studies were also computed across all included driven techniques.

**Figure 8.**
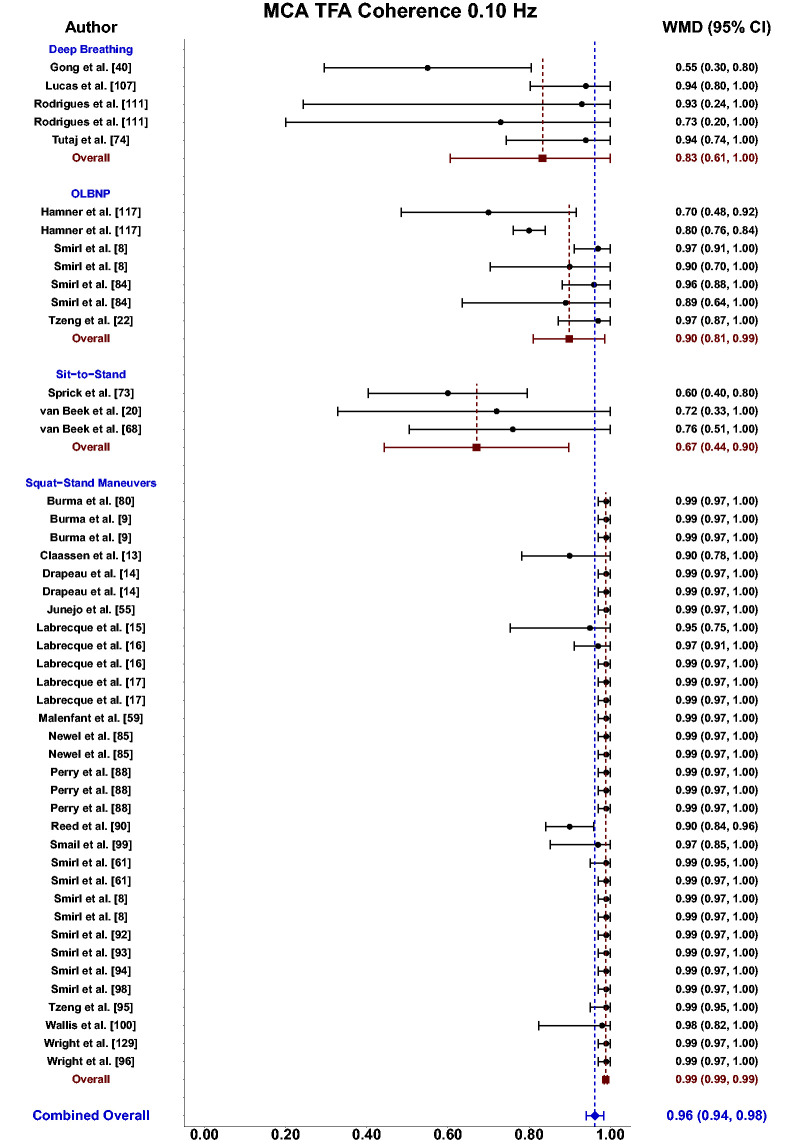
Forest plot depicting the weighted mean difference (WMD) and 95% confidence intervals (CI) for transfer function analysis (TFA) coherence values derived in the middle cerebral artery (MCA) at 0.10 Hz using deep breathing, oscillatory lower body negative pressure (OLBNP), sit-to-stand maneuvers, and squat-stand maneuvers. The pooled estimates from all studies were also computed across all included driven techniques.

Meta-analysis revealed the pooled MAP PSD estimates at 0.10 Hz was 64 mmHg^2^/Hz (95%CI: 0–207; Q-pval = 0.001; I^2^ = 0.86; n = 3) for deep breathing, 634 mmHg^2^/Hz (95%CI: 386–882; Q-pval = 0.968; I^2^ = 0.00; n = 4) for OLBNP, and 11206 mmHg^2^/Hz (95%CI: 7892, 14519; Q-pval < 0.001; I^2^ = 0.63; n = 26) for squat-stands maneuvers (Table 2 and Figure 6). These translated to 0.10 Hz MCA PSD estimates of 17 cm/s^2^/Hz (95%CI: 0–47; Q-pval = 0.566; I^2^ = 0.00; n = 3) for deep-breathing, 359 cm/s^2^/Hz (95%CI: 64–654; Q-pval = 0.806; I^2^ = 0.00; n = 4) for OLBNP, and 9612 cm/s^2^/Hz (95%CI: 6585–12638; Q-pval < 0.001; I^2^ = 0.64; n = 26) for squat-stands maneuvers (Table 2 and Figure 7). Pooled estimates of MCA coherence at 0.10 Hz was 0.83 (95%CI: 0.61–1.00; Q-pval = 0.097; I^2^ = 0.49; n = 5) for deep-breathing, 0.90 (95%CI: 0.81–0.99; Q-pval < 0.001; I^2^ = 0.83; n = 7) for OLBNP, 0.67 (95%CI: 0.44–0.90; Q-pval = 0.597; I^2^ = 0.00; n = 3) for OLBNP, and 0.99 (95%CI: 0.99–0.99; Q-pval = 0.999; I^2^ = 0.00; n = 32) for squat-stands maneuvers (Table 2 and Figure 8). Supplemental F demonstrates greater values were obtained when comparing squat-stands to deep breathing, OLBNP, sit-to-stands at 0.10 Hz for MAP PSD, MCA PSD, and coherence (Wilcoxon *r* range: 0.37–0.65 [moderate-to-large]). Sit-stands elicited greater MAP PSD and MCA PSD compared to deep breathing (Wilcoxon *r*: 0.77 [large]) but lower coherence (Wilcoxon *r*: −0.41) (Supplemental F). Conversely, all aforementioned variables were lower for sit-stands compared to OLBNP (Wilcoxon *r* range: −0.29–0.68 [small-to-large]) (Supplemental F). Finally, OLBNP had greater MAP PSD and MCA PSD (Wilcoxon *r*: 0.80 [large]) compared to deep breathing, with minimal differences for coherence (Wilcoxon *r*: 0.03 [small]) (Supplemental F).

Pooled estimates for phase, gain, and normalized gain for the MCA and PCA across driven techniques are additionally presented in Table 2 with the associated forest plots for the MCA metrics in Supplemental G-L.

### Meta-regression for squat-stand maneuvers

The meta-regression for the TFA metrics from squat-stands are displayed in Supplemental M. Of note, males had greater 0.05 Hz MCA coherence values compared to females (0.05; 95%CI: 0.01, 0.10; p = 0.027) and smaller gain values (−0.14 cm/s/mmHg; 95%CI: −0.28, 0.00; p = 0.050) (Supplemental M). Age showed an inverse relationship with 0.05 Hz MCA PSD (−240 cm/s^2^/Hz; 95%CI: −417, −64; p = 0.010), 0.10 Hz MAP PSD (−262 mmHg/Hz; 95%CI: −484, −40; p = 0.023), and 0.10 Hz MCA PSD (−426 cm/s^2^/Hz; 95%CI: −690, −112; p = 0.009) (Supplemental M). However, sample size had no association with any TFA metrics of interest (all p > 0.071) (Supplemental M).

### Clinical studies using driven techniques

A total of 40 (38%) of the included studies comprised some quantification of dCA within clinical populations that were categorized into clinical groupings of brain injuries (n = 17),^23,24,37–51^ cardiovascular disease/disorders (n = 10),^52–61^ cognitive decline associated with aging (n = 7),^62–68^ pregnancy or preeclampsia (n = 3),^69–71^ migraineurs (n = 1),^72^ kidney disease (n = 1),^73^ and glaucoma (n = 1)^74^ (Supplemental D). From these, techniques used to induce ABP oscillations included deep-breathing (n = 18),^23,24,37,40,43–48,51,52,67,69–72,74^ squat-stands maneuvers (n = 8),^49,50,53,55,58,59,61,62^ sit-to-stand maneuvers (n = 8),^39,54,63–66,68,73^ neck suction (n = 2),^56,60^ leg cuff oscillations (n = 1),^38^ rhythmic handgrip (n = 1),^41^ ventilated breathing (n = 1),^42^ and cyclical pump flow (n = 1).^57^ The majority of deep-breathing techniques (56%)^23,24,40,43–48,51^ were used in patients with artery stenosis and/or occlusion from laboratories based in Germany (89%).^23,24,43–48,51,52,67,69–72,74^ Additionally, the three pregnancy/preeclampsia studies used deep-breathing techniques.^69–71^ Aside from one study where the breathing frequency was unknown,^37^ the remaining 17 had participants breathe at 0.10 Hz (6 breaths per minute).^23,24,40,43–48,51,52,67,69–72,74^ Of the 8 clinical studies using squat-stands maneuvers, 6 completed these at both 0.05 and 0.10 Hz (75%),^49,50,53,59,61,62^ while the other two used 0.10 Hz individually (25%).^55,58^ These investigations included a wide range of clinical populations such as patients with Alzheimer’s disease, dementia, or mild cognitive impairment;^62^ concussion;^49,50^ long-term heart transplant recipients;^53,61^ patients with atrial fibrillation and/or pulmonary arterial hypertension;^55,59^ and at cardiovascular risk/with cardiovascular disease.^58^ The sit-to-stand maneuvers have been completed primarily in individuals with Alzheimer’s disease, dementia, or mild cognitive impairment (63%),^63–66,68^ while the others included mild-to-severe traumatic brain injury,^39^ kidney disease,^73^ and those using a left ventricular assist device.^54^ These maneuvers were completed at 0.05 Hz only in 6 studies (75%),^39,54,63–66^ while the remaining 2 studies used both 0.05 and 0.10 Hz.^68,73^ Across studies that compared clinical populations to healthy controls, 19 reported reduced autoregulatory metrics.^23,24,41,43,44,46–50,52,55,56,59,60,67,68,71,74^ These populations included artery stenosis or occlusion (n = 7);^23,24,43,44,46–48^ concussion (n = 2);^49,50^ Type II Diabetes (n = 2);^52,60^ Alzheimer’s disease, dementia, and mild cognitive impairment (n = 1);^68^ early postpartum (n = 1);^71^ arterial fibrillation (n = 1);^55^ ischemic stroke (n = 1);^41^ cirrhosis and portal hypertension (n = 1);^56^ pulmonary arterial hypertension (n = 1);^59^ cerebral amyloid angiopathy (n = 1);^67^ and glaucoma (n = 1).^74^

### General strengths and weaknesses of methods employed to enhance TFA signal-to-noise ratios

#### Squat-stand maneuvers

This is the most widely used method for enhancing the signal-to-noise ratio within the driven dCA literature (Figure 2 and Supplemental C).^8,9,12–17,28,49,50,53,55,58,59,61,62,75–100^ The first study employing this technique was performed by Birch and colleagues in 1995^12^ and investigated the changes to dCA phase during hypocapnia, eucapnia, and hypercapnia. In this study, it was revealed squat-stands maneuvers are feasible, require no additional equipment, and can elicit very large and robust ABP and CBv oscillations.^12^ Since this seminal work, there have been many follow-up studies demonstrating there are several ways in which the repeated squat-stands maneuvers can be performed, with participants either going to: maximal depth,^101^ moderate level (90-degree bend at the back of the knee),^8^ or a shallow squat (45-degree bend at the back of the knee).^101^ The deep and shallow squats appear to elicit oscillations of ∼15–20 mmHg (from minimum to maximum) in ABP and ∼30–50 cm/s in CBv,^101^ whereas the mid-range tend to elicit slightly larger ABP swings in the 30–50 mmHg range and CBv swings of ∼40–70 cm/s.^8,9,28,85,94,98^ The main difference in the approaches appears to be a greater engagement of the skeletal muscle pump^102,103^ during the squats with a 90-degree bend in the back of the knee, which augments the Frank-Starling mechanism.^103^ This increased venous return with the active engagement of the lower limb muscles during squat-stands maneuvers likely explains the enhanced coherence and reproducibility of this approach over the passive seating and lack of lower limb contractions that occur during the sit-to-stand maneuvers. Using the squat-stands maneuvers within driven dCA investigations has consistently been shown to result in coherence values approaching 1.00 in both the MCA and PCA (Figures 5 and 8). Most importantly, this method has also been shown to have the greatest intraindividual reproducibility for the associated TFA phase and gain metrics in both younger and older adults.^8^ As such, this approach has been referred to as the *“gold-standard”* for the linear interpretation of dCA assessed through TFA.^8^ Nevertheless, squat-stands maneuvers are not without limitation. Completing five minutes of repeated squat-stands maneuvers may be difficult to sustain and/or are not feasible for several clinical populations (e.g., spinal cord injury, chronic kidney disease, stroke, etc.). The mild change in posture has been shown to produce ∼2–3 Torr swings in P_ET_CO_2_, which may additionally slightly alter CBv. Further, a consideration with squat-stand maneuvers is that the large MAP oscillations produced may overwhelm the cerebrovasculature by providing a stimulus that surpasses the autoregulatory limit. Nevertheless, a previous investigation denoted the signalling of the neurovascular coupling response remains intact during squat-stand maneuvers at both 0.05 and 0.10 Hz minimizing this concern.^104^ Despite the limitations, it is the recommendation of this review, that whenever possible, repeated squat-stands maneuvers should be performed to maximize the signal-to-noise ratio between ABP and CBv as it has the highest coherence values and most reproducible TFA phase and gain metrics.^8^

#### Deep/paced breathing

Deep/paced breathing is the second most common method for driving oscillations in ABP for driven dCA research investigations. It has been employed in 24 research studies to date (Figure 2 and Supplemental C),^23,24,37,40,42–48,51,52,67,69–72,74,105–111^ with the first research investigation performed by Diehl et al., in 1995.^23^ Most often this method involves controlling breathing rates at 0.10 Hz, although 0.167 Hz^105,108,110^ and 0.25 Hz^108,110^ have also been employed. This approach can elicit the desired effect of creating ∼10–20 mmHg oscillations in ABP^23,24,47,112^ through a complex series of physiological mechanisms including alterations to the respiratory, autonomic nervous, cardiorespiratory, and cardiovascular systems.^113^ The main benefit of this approach to augment ABP oscillations is that minimal specialized equipment is required outside of a standard inline gas analyzer^4,5^ and can moderately increase coherence (0.83; 95%CI: 0.29, 1.00) (Figure 8). Albeit the 95%CI are variable due to only a few studies reporting coherence in healthy participants. This approach also has a substantial limitation, namely these interactions can often result in a significant reduction in P_ET_CO_2_ levels of ∼2 to >5 Torr across the 5-minute breathing period where dCA data is collected and analyzed. While this P_ET_CO_2_ reduction, often has minimal effect on the mean level of ABP during the data collection period, it gradually reduces CBv across the same time period by ∼2 to >7 cm/s,^47^ which in turn will also affect the associated TFA phase (artificially increased) and gain (artificially decreased) across the data collection period. Therefore, this approach, while widely used (Figure 2 and Supplemental C), also has a direct impact on the dCA metrics being examined in these investigations and therefore is not recommended as a primary method for augmenting the signal-to-noise ratio. Nevertheless, a solution to augment the internal validity of this approach is to perform this technique during concurrent end-tidal clamping to ensure P_ET_CO_2_ values remain constant.

#### Oscillatory lower body negative pressure

Although this method requires some very specialized equipment, namely an OLBNP chamber, it is the third most common method for driving ABP oscillations in the driven dCA research field (Figure 2 and Supplemental C).^8,21,22,114–130^ In brief, this method was proposed to alter ABP by reducing the atmospheric pressure surrounding the lower body and thus reducing the transmural pressures being applied to the compliant vessels in this portion of the body.^115^ Therefore, when the LBNP is applied in a cyclical manner (i.e., OLBNP), there will be induced oscillations on the venous blood flow returning to the heart, altering the Frank-Starling mechanism of the heart^131,132^ as well as the activation of the baroreceptors.^133–135^ This method was first employed in 2002 by Birch, Neil-Dwyer, and Murrills,^115^ who evoked a negative pressure between −98 and −130 Torr, which induced swings in ABP of ∼20 mmHg and ∼30 cm/s for CBv at a frequency of 0.083 Hz. Since this first investigation, there have been multiple iterations of OLBNP protocols with negative pressures generated between −20 Torr and −150 Torr, and at frequencies of 0.01, 0.03, 0.04, 0.05, 0.06, 0.07, 0.08, 0.083, 0.10, 0.167, and 0.25 Hz (Supplemental C). This truly highlights the utility and level of control that is available for this approach to create ABP oscillations in the driven dCA research field. There is no other method within this review that was capable of this breadth of applications (Supplemental C). When examining the outcomes, it appears as though TFA coherence was the highest for the 0.10 Hz oscillations (0.90; 95%CI: 0.81, 0.99) compared to 0.05 Hz oscillations (0.70; 95%CI: 0.59, 0.82) (Table 2 and Figures 3 and 4). This was the second highest coherence level across all methods that had sufficient sample sizes to be included in the pooled estimates. This is likely due to OLBNP eliciting the second greatest magnitude increase in MAP at 0.10 Hz (Figure 6). A downside to this approach, other than the requirement for the specialized equipment needed, appears to be the limited reproducibility that was shown in both younger and older adults.^8^ Despite coherence levels staying within ∼10% for between-day coefficient of variation metrics, individual participant TFA phase values were shown to vary by ∼15% to 75% between days and TFA gain was similar at a range of ∼10% to 80% between days.^8^ Further, the application of LBNP has been suggested to result in deeper inhalations, which in turn may influence P_ET_CO_2_ values.^136^ Researchers employing these methods must be cognizant of participants’ breathing patterns. With additional development into standardizing the extent of negative pressure application along with the frequency of oscillations, this method holds promise for future research investigations. However, with the broad applications that are currently in the field, it makes comparing across studies challenging.

#### Sit-to-stand maneuvers

Although a relatively recent addition to the TFA-based driven dCA field (first study published in 2010),^20^ this is one of the most widely used methods for evoking oscillations in ABP and CBv (Figure 2 and Supplemental C).^18–20,39,54,63–66,68,73,137–141^ In this approach, participants are asked to repeatedly sit down onto a standard height chair and then stand-up following set intervals. This is a relatively simple maneuver for most people to perform and can be executed within a wide range of clinical populations (Supplemental D). To date, there are two main frequencies that have been used in the broader literature (Figure 2 and Supplemental C), one for a 10-s sit, followed by a 10-s stand (0.05 Hz) and another slightly faster sequence with a 5-s sitting period, followed by a 5-s standing cycle (0.10 Hz). These frequencies were selected to elicit ABP (∼15–20 mmHg) and CBv (∼10–15 cm/s) oscillations within the very low (0.02–0.07 Hz) and low (0.07–0.20 Hz) frequency bands.^4,5,7^ Performing these maneuvers often results in improved TFA coherence; however, there is still quite a range in the reported coherence values (Table 2). The sit-to-stand maneuvers performed at 0.05 Hz appear to have a higher coherence (0.87; 95%CI: 0.79, 0.95) and much better consistency in the associated phase and gain metrics, as compared with ones executed at 0.10 Hz (0.67; 95%CI: 0.44, 0.90). Overall, this method of creating driven oscillations in ABP and CBv is widely used, and able to be performed within clinical populations, which are strong positive aspects for this technique. Unfortunately, the major drawback is the variation in the reported coherence values, especially at 0.10 Hz (Figure 8). Furthermore, when examining the TFA phase metrics between 0.05 and 0.10 Hz, the expected high-pass filter nature of dCA^4,5,7^ does not appear to be intact using this technique as the mean pooled phase value actually increases from 0.05 to 0.10 Hz (instead of decreasing as would be expected), which limits our ability to recommend this technique for future research investigations.

#### Neck suction

Three investigations used a neck suction technique to cause ABP oscillations through carotid baroreceptor stimulation (Figure 2 and Supplemental C).^56,60,142^ Lagi et al.,^56^ performed the first investigation in 2002 where participants were attached to a neck chamber that was connected to a vacuum and modulated by a specifically designed control unit. The three investigations completed these at 0.10 Hz independently. Two of the studies did not report the amplitude of the oscillations, while Purkayastha and colleagues^142^ used oscillations between 0 and +40 Torr. This stimulus produced CBv oscillations of ∼5–6 cm/s; however, the ability of the neck suction technique to augment coherence was modest with 0.10 Hz point estimates of ∼0.60–0.65.^142^ Similar to the OLBNP technique, a limitation of neck suction application is the specialized equipment required to produce ABP oscillations. The application of pressure to the neck may not be preferable to some individuals and/or clinical populations, potentially leading to selection bias in these studies. Given the limited ability of the neck suction technique to produce a meaningful increase in coherence, this technique is not currently recommended.

#### Head-up tilt

Repeated head-up tilt has been used to evoke oscillations in ABP for dCA analysis in two studies to date (Figure 2 and Supplemental C).^110,143^ The original investigation in this domain in 2001 by Hughson et al.,^143^ was performed by rapidly tilting individuals from a supine position to a 45-degree head-up tilt at 0.05 Hz. In the follow-up investigation in 2019 by Stok et al.,^110^ participants were tilted in a slow and consistent sinusoidal movement from supine to a 60-degree head-up tilt for four separate 2-minute periods at 0.042, 0.067, 0.10, and 0.167 Hz (ranging from 5 to 20 cycles in each 2-minute segment). The changes to the angle of the head relative to the heart during these induced oscillations are important to note as it will also result in a concomitant change to the cerebral perfusion pressure (CPP; mean arterial – intracranial pressure), with the supine posture resulting in a substantially higher CPP than the head-up tilt position.^143^ These posture influences also evoke substantially larger swings in CBv (∼15 cm/s at the start of the tilt period to ∼5 cm/s at the end of the same frequency period) than ABP (relatively consistent at ∼5–10 mmHg across the entire frequency period).^110^ One of the main factors affecting the gradual reduction in CBv swing depth during the head-up tilt, is a concurrent reduction in P_ET_CO_2_ of ∼5–10 Torr during the head-up tilt period. Unfortunately, neither study reported TFA coherence during the head-up tilt aspects of the respective investigations and therefore firm conclusions are unable to be made on the signal-to-noise augmentation associated with these protocols. It is expected the coherence would have increased as a result of rhythmic head-up tilt. A major limitation of this approach, however, is the consistent P_ET_CO_2_ reduction throughout the head-up tilt period will have a substantial impact on the associated TFA phase and gain, which will make the interpretation of these metrics very challenging. Additionally, there is some specialized equipment required (a tilt table), which can also limit the ability of this approach to be employed in a widespread manner.

#### Leg/thigh cuff inflation/deflation

Two research studies have employed repeated leg/thigh cuff inflation/deflation techniques to evoke oscillations in ABP and CBv for TFA based dCA assessments (Figure 2 and Supplemental C).^38,144^ In the first investigation in 2007 by Aaslid and colleagues^38^, the stimulus was evoked by rapidly inflating a set of large leg cuffs above systolic ABP for a period of 15 s, at which point they were rapidly deflated to 0 mmHg for another 15 s. This period was repeated for a minimum of 8 cycles (0.033 Hz) and evoked swings of ∼15 mmHg for ABP and ∼8 cm/s for CBv. In the second investigation in 2012 by Katsogridakis et al.,^144^ thigh cuffs were rapidly inflated (and deflated) from 10 mmHg to either 80 or 150 mmHg at frequencies of 0.05, 0.10, and 0.025 Hz. This approach also evoked ABP swings of ∼15 mmHg; however, the CBv oscillations were only ∼5 cm/s. Neither study reported P_ET_CO_2_ data and therefore no interpretations can be made with respect to the effects of P_ET_CO_2_ on the TFA outcome metrics. Furthermore, only the study by Katsogridakis et al.,^144^ reported coherence values and these were surprisingly low (0.30–0.65) and indicate the thigh cuff maneuvers are likely not sufficient to evoke substantial and consistent changes in ABP and CBv. Therefore, the additional specialized equipment required for this form of driven dCA assessment (rapid thigh cuff inflation/deflation systems) along with the limited (or non-existent) augmentation to TFA coherence and reported discomfort associated with cuff inflation,^25^ restricts the ability to recommend this approach for future driven dCA assessments.

#### Cyclical pump flow

This is a very rare method within the broader literature and has been used in just one study to date (Figure 2 and Supplemental C)^57^ for enhancing the input-output relationship between ABP and CBv. This is due to it involving a very specialized clinical population (patients undergoing cardiopulmonary bypass) and extensive specialized equipment.^57^ In this study, variations in ABP were created through the centrifugal pump transitioning from 2.4 L/min/m^2^ up to 2.8 L/min/m^2^ back to 2.4 L/min/m^2^ and then down to 2.0 L/min/m^2^ in a cyclical step-wise manner at a rate of 0.10 Hz, resulting in an oscillation of ∼20 mmHg and ∼10 cm/s, for ABP and CBv, respectively. Without a driven stimulus during cardiopulmonary bypass surgery, there are virtually no oscillations in ABP and therefore it would be inappropriate to attempt to determine the dCA response under this clinical procedure. When employed, this method demonstrated it was very effective at inducing the desired increase in coherence at the point-estimate of 0.10 Hz (approaching 1.00) and alterations in TFA phase (increased with hypocapnia, decreased with hypercapnia) and TFA gain (decreased with hypocapnia, increased with hypercapnia) that are consistent with the broader literature.^12,145^ The major limitation of this technique is that it is not ethical, nor feasible, to be performed in the general population as it requires a great deal of invasive surgery, many medications, and specialized equipment.

#### Passive leg raises

Passive leg raises were only performed in a single-driven dCA research investigation (Figure 2 and Supplemental C).^25^ Elting et al. (2014)^25^ proposed the application of passive leg raises as a way to evoke venous blood shifts to alter ventricular preload and thus create oscillations in ABP in clinical settings with minimal specialized equipment required. Participants were positioned in an adjustable medical chair in a supine posture and then their legs were rapidly lifted (∼1 s) to about a 60-degree angle, held for 4 s and then lowered back to the horizontal plane over a ∼1 s period (10 s total cycle: 0.10 Hz). The passive leg raises resulted in ABP swings of ∼5–10 mmHg along with ∼5 cm/s swings in CBv.^25^ There was also a slight reduction in P_ET_CO_2_ that was observed during the passive leg raises. Overall, it was noted, the inclusion of passive leg raises resulted in no change to TFA coherence over the spontaneous oscillations that were present during the supine rest in the medical chair and no consistent changes observed to TFA phase and gain metrics. The authors of this prior investigation concluded their overall results were disappointing, and the authors of the current review article agree. Passive leg raises are not able to be recommended for augmenting coherence and improving the interpretability of dCA phase and gain metrics despite being well-tolerated and requiring minimal patient co-operation.^25^

#### Rhythmic handgrip contractions

The approach to drive ABP oscillations via rhythmic handgrip was performed once in the driven dCA field in 2004 (Figure 2 and Supplemental C).^41^ These researchers examined a population that had suffered from an ischemic MCA stroke and had the participants perform rhythmic handgrip contractions using the unaffected arm by squeezing a sphygmomanometer cuff. The contractions were performed at 20% of a 10-s maximal contraction strength, held for 20 s and released for 20 s (0.025 Hz).^41^ It was reported that ∼10 to 15 mmHg swings in ABP occurred at this frequency. No data was reported on the CBv alterations, coherence, nor P_ET_CO_2_ during the rhythmic handgrip contractions. The general strength of this method is the applicability for use in this clinical setting; however, the evoked oscillation for ABP is relatively small and without additional information provided for CBv swings nor TFA coherence outcomes, it is not possible to recommend this approach for broader applications in its current form.

#### Ventilated breathing

To date, mechanically-ventilated breathing for the purpose of augmenting the signal-to-noise ratio in driven dCA has only been performed in a single study (Figure 2 and Supplemental C).^42^ In this investigation, severely brain injured patients were mechanically ventilated at 6 breaths per minute (0.10 Hz), which resulted in oscillations of ∼5–15 mmHg in ABP and ∼1–4 mmHg in intracranial pressure (ICP).^42^ While coherence was not directedly reported in this investigation, it was stated that coherence was above 0.50 and P_ET_CO_2_ was unchanged during this investigation. This is a very novel and interesting manner to evoke clear oscillations in ABP and ICP in a clinical bed-rest population. However, the very specialized equipment and invasive nature in which the ABP oscillations were developed limits this approach in other non-clinical domains.

## Discussion

The purpose of this systematic review was to amalgamate the literature assessing the cerebral pressure-flow relationship using driven techniques in both healthy and clinical populations. Each of these techniques were discussed in detail regarding the benefits, limitations, and recommendations for researchers/clinicians to consider. Further, the clinical studies were summarized with an aim to guide future high-quality research studies and to promote more homogeneity across the field of dCA. Of importance, the current review displays pooled estimates across a multitude of studies using deep-breathing, OLBNP, sit-to-stand, and squat-stands maneuvers that provide additional reference values future studies can compare healthy and clinical populations against.

### Future directions for driven autoregulation assessments

Based upon the prior research employing these 11 different techniques for augmenting the signal-to-noise ratio in driven dCA investigations, it is apparent there have been many different approaches that have shown various levels of improvements in the TFA coherence metric (Table 2 and Figures 3 [Fig fig4-0271678X241235878][Fig fig5-0271678X241235878][Fig fig6-0271678X241235878][Fig fig7-0271678X241235878]to 8). Based on the summarized findings for each of these approaches, researchers should employ squat-stands maneuvers to a 90-degree bend at the back of the knee whenever possible as it has consistently been revealed to have the highest coherence and least variance in the associated phase and gain values (Table 2 and Figures 3 [Fig fig4-0271678X241235878][Fig fig5-0271678X241235878][Fig fig6-0271678X241235878][Fig fig7-0271678X241235878]to 8). The physiological explanation for this was detailed above in the results section. This method does appear to be the *“gold-standard”*^8^ for creating the optimal signal-to-noise ratio^4,5,7^ and creating the most linear system between ABP and CBv for interpreting the associated TFA phase and gain metrics.

In the current analysis, TFA PSD, coherence, phase, gain, and normalized gain were pooled where sex, age, and sample sizes were able to be included in meta-regression analyses as covariates of interest. Nonetheless, other factors, such as cardiorespiratory fitness,^14,16,17^ sex hormones, contraceptive usage, cytokines, and blood biomarkers may have had a modifying influence on the TFA estimates. However, few studies have been conducted examining these influences. Exploration into these factors is highly recommended to better understand the specific factors researchers should seek to control to maximize the internal validity of a given study. Accumulating evidence suggests regulation within the cerebral pressure-flow relationship differs across the cardiac cycle.^9,50,80,85,94,146,147^ These investigations have largely been conducted using TFA metrics, and thus application into time-domain and other analyses are warranted. Finally, the use of other imaging modalities [e.g., functional near-infrared spectroscopy (fNIRS)] and the monitoring of volumetric cerebral blood flow and cerebral perfusion pressure (i.e., mean arterial pressure – intracranial pressure) in future studies are encouraged to provide more meaningful results for metrics quantifying dCA across different brain regions.

### Clinical implications

A single technique did not emerge in its ability to demarcate clinical from healthy populations. Deep-breathing and squat-stand maneuvers were able to identify clinical differences 61% and 50% of the time, respectively; while sit-to-stands demonstrated less than favorable results at 13%. Nonetheless, a lack of difference does not necessarily directly lead to the proposition that a given driven technique is more clinically relevant than another. For example, the studies using squat-stand maneuvers in long-term heart transplant recipients identified a lack of differences compared to age-matched and donor-matched controls. The null findings do not reduce the sensitivity of squat-stand maneuvers but rather highlight a clinical population where dCA remains intact. Ultimately, to identify the specific driven approaches that have the best sensitivity and specificity for demarcating impairments within the cerebral pressure-flow relationship, completing three or four driven approaches in a population that is known to have impairments would be fruitful. A final issue with respect to creating clinical pooled estimates is the lack of description of uncontrolled confounding influences. These may additionally have an interaction effect in clinical studies making it more complex to compute pooled estimates (e.g., sex and systolic blood pressure). Nevertheless, these findings highlight the importance of clearly defining the inclusion and exclusion criteria of a clinical population, which will make it easier in the future to produce more homogenous clinical pooled dCA estimates.

In clinical studies, spontaneous protocols generally result in low amplitude ABP oscillations, which may contribute to insufficient coherence for reliable TFA estimates.^8^ One way to deal with this issue would be to examine the minimal amplitude of ABP oscillation required for a given population to adequately quantify dCA using TFA. However, researchers should be cognizant of the safety and feasibility of inducing large and rapid ABP oscillations over the course of more than a few minutes for some of these clinical populations (e.g., stroke, traumatic brain injury, etc.). Further, while spontaneous measures have been recommended for safety reasons within clinical populations,^11^ it cannot be overstated the underlying physiology should not be compromised simply for the sake of the mathematical analysis, given the poor reproducibility spontaneous measures have consistently produced.^8^ Therefore, it is recommended the safest technique that also induces the largest ABP oscillations be used with respect to the population of interest. For example, while squat-stands maneuvers elicit the largest ABP challenges, using such large transient ABP oscillations in patients with acute heart failure or hemorrhagic stroke is not recommended for safety reasons as this could result in further complications. However, driven techniques such as squat-stands maneuvers have been used in long-term heart-transplant recipients^53,61^ and in patients with atrial fibrillation^55^ and pulmonary arterial hypertension^59^, once physiologically stable and in the presence of trained healthcare clinicians. Driven techniques in acute pathological conditions, if deemed necessary, should routinely be conducted within a hospital and/or location that has medical assistance ready to intervene if necessary.

In the case where driven techniques are unable to be used in clinical populations where sufficient ABP oscillations cannot be elicited, it may be necessary for dCA assessments in these populations to move beyond TFA. Rather the use of metrics that rely less on the assumption of linearization may be favourable. One argument in support of this view is that by completely linearizing the cerebral-pressure flow system, such as when using squat-stands maneuvers, we may be getting the information from the system in its linear state (i.e., very high coherence) with high accuracy. However, by doing so, it may override other physiological components that normally contribute to the system with respect to time-domain analysis and/or other metrics. Thus, dCA quantification in the presence of lower coherence should not be necessarily discarded, as it could offer their own proper insights on the behaviour of dCA. In conjunction, cohesion between a given dCA analysis method, underlying physiological mechanisms, and clinical outcomes would be a decisive requirement to ensure the validity and reliability of a given autoregulatory approach. Other proposed analyses include wavelet decomposition analysis, pursuit regression analysis, principal dynamic mode analysis, as well as analytical methods examining the closed-loop interactions between ABP and CBv such as the Granger causality analysis and conditional transfer entropy analysis, which have all been detailed elsewhere.^3^

While driven techniques have been employed across a wide range of clinical populations, it is imperative readers assess the statistical analyses, control of confounding variables, and the specific driven techniques used as these may contribute to sources of measurement and/or misclassification biases. Despite this, the results in the current investigation highlight the safety and feasibility of using driven approaches to quantify the cerebral pressure-flow relationship in cerebrovascular, cardiovascular, and neurological disease; changes associated with healthy aging; and even during pregnancy. Nonetheless, to fully understand the autoregulatory deficits that underpin different disease/clinical presentations, it is imperative the most robust techniques are used that lead to reliable and valid conclusions.

## Limitations

A main limitation of the current review is due to only the PubMed database being indexed. Nevertheless, this database publishes the majority of physiology journals and articles examining the cerebral pressure-flow relationship. Additionally, to be exhaustive, the authors screened the reference lists of included articles to identify any article that were potentially missing; however, no additional articles were missed by the search strategy. The MINORS items that most affected the risk of bias and internal validity of the included articles were the absence of a blinded evaluation of the outcome measures during the analysis stage and the lack of reporting a sample size calculation (4%). Very few studies controlled for confounding factors such as biological sex, age, hormonal cycle, cardiorespiratory fitness, and P_ET_CO_2_ values. Hence, the results of this review should be considered with caution when extended to females and children/adolescents, as these were largely underrepresented populations (Figure 2). Articles including elderly populations consisted of only 36% (Table 2) of the articles included in this analysis, with the majority of these comprising only males. It is postulated elderly females and males may have a greater degree of similarity with respect to dCA, once females become post-menopausal;^148^ however, future research is warranted to confirm this proposition. Only studies estimating cerebral blood flow changes using transcranial Doppler ultrasound were included in the current analysis. This modality was chosen as it has been the most used to quantify dCA due to its high temporal resolution. Another technique that could serve as an alternative to transcranial Doppler ultrasound is fNIRS. However, the correlation between both modalities is low,^64^ which is likely due to fNIRS measuring the microvasculature, while transcranial Doppler ultrasound insonates intracranial arteries. Therefore, differences in the temporal characteristics and waveform morphology make data difficult to compare. Due to the lack of driven studies using fNIRS and the heterogeneous outcomes, studies including metrics derived from fNIRS monitoring were not considered in the current systematic review.

Meta-analyses were only capable of being conducted on TFA metrics as it is the most popular and widely used analytical approach to assess dCA.^4,5^ A major limitation when interpreting TFA metrics is its assumption for linearity, which is tested by the coherence metric. A low coherence can be explained by extraneous noise in the signal, a nonlinear system relating input and output, changes in the output due to more than one input, an absence of a relationship between input and output, or a combination of these factors. Therefore, in the presence of low coherence, it is impossible to assume linearity, which limits the interpretation of TFA metrics due to more variability.^8,9^ The use of other analytical methods is warranted to better understand the cerebral pressure-flow relationship in the frequency- (non-linear analysis) (e.g., wavelet decomposition analysis, projection pursuit regression) and time-domains (e.g., autoregulatory index, rate of regulation, directional sensitivity, mean flow index in response to cerebral perfusion pressure/arterial blood pressure, autoregressive-moving average models). It should be noted some of the aforementioned approaches have primarily been used during spontaneous dCA assessments. Hence, the implementation of these statistical analyses with driven ABP may unveil more information regarding the physiological underpinnings of dCA. It is thus recommended multiple analyzing models be used when examining dCA to provide a comprehensive analysis of the cerebral pressure-flow relationship.

## Conclusions

This systematic review examined the studies to date that have assessed the cerebral pressure-flow relationship via various driven techniques. These have been completed across a wide array of both healthy and clinical populations, with the most common analysis conducted being TFA at 0.05 and/or 0.10 Hz. Given this, meta-analyses were performed, where pooled TFA estimates (coherence, phase, and gain) and the associated 95% confidence intervals were derived for deep breathing, OLBNP, sit-to-stand maneuvers, and squat-stands maneuvers. These pooled estimates provide additional reference values studies can compare their healthy and clinical populations against. While each driven technique has its benefits and limitations, future studies should seek to utilize methodological approaches that induce the greatest degree of ABP oscillations. Nonetheless, consideration is required for acute/unstable clinical populations, where driven techniques may be unsafe/impractical.

## Supplemental Material

sj-pdf-1-jcb-10.1177_0271678X241235878 - Supplemental material for A systematic review, meta-analysis, *and* meta-regression amalgamating the driven approaches used *to* quantify dynamic cerebral autoregulation Supplemental material, sj-pdf-1-jcb-10.1177_0271678X241235878 for A systematic review, meta-analysis, *and* meta-regression amalgamating the driven approaches used *to* quantify dynamic cerebral autoregulation by Joel S Burma, Marc-Antoine Roy, Courtney M Kennedy, Lawrence Labrecque, Patrice Brassard and Jonathan D Smirl in Journal of Cerebral Blood Flow & Metabolism

## Data Availability

Data are available upon reasonable request to the corresponding author (JSB).
