# Supplementary material for: A systematic review, meta-analysis and meta-regression amalgamating the driven approaches used to quantify dynamic cerebral autoregulation
Source: J Cereb Blood Flow Metab. 2024 Apr 18;44(8):1271–97. doi: 10.1177/0271678X241235878 (PMC11342731; doi:10.1177/0271678X241235878)
Supplement: sj-pdf-1-jcb-10.1177_0271678X241235878 - Supplemental material for A systematic review, meta-analysis, and meta-regression amalgamating the driven approaches used to quantify dynamic cerebral autoregulation [file sj-pdf-1-jcb-10.1177_0271678X241235878.pdf]

Autoregulation is a highway, I wanna drive it: A systematic review, meta-analysis, and meta-regression amalgamating the driven approaches used to quantify dynamic cerebral autoregulation.

Joel S. Burma, Marc-Antoine Roy, Courtney M. Kennedy, Lawrence Labrecque, Patrice Brassard, Jonathan D. Smirl

Supplemental Materials

Table of Contents

Supplemental A: Bibliography of Included Articles 2

Supplemental B: Included Studies and Risk of Bias Scoring 11

Supplemental C: Study Characteristics and Demographics. 16

Supplemental D: Studies Using Driven Techniques in Clinical Populations. 25

Supplemental E: 0.05 Hz TFA Wilcoxon r Effect Sizes 28

Supplemental F: 0.10 Hz TFA Wilcoxon r Effect Sizes 29

Supplemental G: Forest Plot of Middle Cerebral Artery 0.05 Hz Phase 30

Supplemental H: Forest Plot of Middle Cerebral Artery 0.05 Hz Gain.................................... 31

Supplemental I: Forest Plot of Middle Cerebral Artery 0.05 Hz Normalized Gain 32

Supplemental J: Forest Plot of Middle Cerebral Artery 0.10 Hz Phase................................... 33

Supplemental K: Forest Plot of Middle Cerebral Artery 0.10 Hz Gain 34

Supplemental L: Forest Plot of Middle Cerebral Artery 0.10 Hz Normalized Gain.................. 35

Supplemental M: Meta-Regression Beta Coefficients and 95% Confidence Intervals for Squat-Stand Maneuver TFA Estimates 35

Supplemental A: Bibliography of Included Articles

Aaron SE, Hamner JW, Ozturk ED, Hunt DL, Iaccarino MA, Meehan WP, 3rd, Howell DR, Tan CO (2021) Cerebrovascular Neuroprotection after Acute Concussion in Adolescents. Ann Neurol 90 (1):43-51. doi:10.1002/ana.26082

Aaslid R, Blaha M, Sviri G, Douville CM, Newell DW (2007) Asymmetric dynamic cerebral autoregulatory response to cyclic stimuli. Stroke 38 (5):1465-1469. doi:10.1161/strokeaha.106.473462

Abbariki F, Roy MA, Labrecque L, Drapeau A, Imhoff S, Smirl JD, Brassard P (2022) Influence of high-intensity interval training to exhaustion on the directional sensitivity of the cerebral pressure-flow relationship in young endurance-trained men. Physiol Rep 10 (13):e15384. doi:10.14814/phy2.15384

Aengevaeren VL, Claassen JA, Levine BD, Zhang R (2013) Cardiac baroreflex function and dynamic cerebral autoregulation in elderly Masters athletes. J Appl Physiol (1985) 114 (2):195-202. doi:10.1152/japplphysiol.00402.2012

Anderson GK, Rosenberg AJ, Barnes HJ, Bird J, Pentz B, Byman BRM, Jendzjowsky N, Wilson RJA, Day TA, Rickards CA (2021) Peaks and valleys: oscillatory cerebral blood flow at high altitude protects cerebral tissue oxygenation. Physiol Meas 42 (6). doi:10.1088/1361-6579/ac0593

Barnes SC, Ball N, Haunton VJ, Robinson TG, Panerai RB (2017a) The cerebrocardiovascular response to periodic squat-stand maneuvers in healthy subjects: a time-domain analysis. Am J Physiol Heart Circ Physiol 313 (6):H1240-h1248. doi:10.1152/ajpheart.00331.2017

Barnes SC, Ball N, Haunton VJ, Robinson TG, Panerai RB (2018) How many squat-stand manoeuvres to assess dynamic cerebral autoregulation? Eur J Appl Physiol 118 (11):2377-2384. doi:10.1007/s00421-018-3964-2

Barnes SC, Ball N, Panerai RB, Robinson TG, Haunton VJ (2017b) Random squat/stand maneuvers: a novel approach for assessment of dynamic cerebral autoregulation? J Appl Physiol (1985) 123 (3):558-566. doi:10.1152/japplphysiol.00316.2017

Birch AA, Dirnhuber MJ, Hartley-Davies R, Iannotti F, Neil-Dwyer G (1995) Assessment of autoregulation by means of periodic changes in blood pressure. Stroke 26 (5):834-837. doi:10.1161/01.str.26.5.834

Birch AA, Neil-Dwyer G, Murrills AJ (2002) The repeatability of cerebral autoregulation assessment using sinusoidal lower body negative pressure. Physiological measurement 23 (1):73-83. doi:papers3://publication/uuid/0A2F942E-843F-41B7-9B71-FCD7FF26DEE3

Brassard P, Ferland-Dutil H, Smirl JD, Paquette M, Le Blanc O, Malenfant S, Ainslie PN (2017) Evidence for hysteresis in the cerebral pressure-flow relationship in healthy men. Am J Physiol Heart Circ Physiol 312 (4):H701-h704. doi:10.1152/ajpheart.00790.2016

Brothers RM, Zhang R, Wingo JE, Hubing KA, Crandall CG (2009) Effects of heat stress on dynamic cerebral autoregulation during large fluctuations in arterial blood pressure. J Appl Physiol (1985) 107 (6):1722-1729. doi:10.1152/japplphysiol.00475.2009

Brown CM, Dütsch M, Ohring S, Neundörfer B, Hilz MJ (2004) Cerebral autoregulation is compromised during simulated fluctuations in gravitational stress. Eur J Appl Physiol 91 (2-3):279-286. doi:10.1007/s00421-003-0965-5

Brown CM, Marthol H, Zikeli U, Ziegler D, Hilz MJ (2008) A simple deep breathing test reveals altered cerebral autoregulation in type 2 diabetic patients. Diabetologia 51 (5):756-761. doi:10.1007/s00125-008-0958-3

Burma JS, Copeland P, Macaulay A, Khatra O, Smirl JD (2020a) Comparison of diurnal variation, anatomical location, and biological sex within spontaneous and driven dynamic cerebral autoregulation measures. Physiol Rep 8 (11):e14458. doi:10.14814/phy2.14458

Burma JS, Copeland P, Macaulay A, Khatra O, Wright AD, Smirl JD (2020b) Dynamic cerebral autoregulation across the cardiac cycle during 8 hr of recovery from acute exercise. Physiol Rep 8 (5):e14367. doi:10.14814/phy2.14367

Burma JS, Kennedy CM, Penner LC, Miutz LN, Galea OA, Ainslie PN, Smirl JD (2021a) Long-term heart transplant recipients: heart rate-related effects on augmented transfer function coherence during repeated squat-stand maneuvers in males. Am J Physiol Regul Integr Comp Physiol 321 (6):R925-r937. doi:10.1152/ajpregu.00177.2021

Burma JS, Miutz LN, Newel KT, Labrecque L, Drapeau A, Brassard P, Copeland P, Macaulay A, Smirl JD (2021b) What recording duration is required to provide physiologically valid and reliable dynamic cerebral autoregulation transfer functional analysis estimates? Physiol Meas 42 (4). doi:10.1088/1361-6579/abf1af

Carter SE, Draijer R, Holder SM, Brown L, Thijssen DHJ, Hopkins ND (2018) Regular walking breaks prevent the decline in cerebral blood flow associated with prolonged sitting. J Appl Physiol (1985) 125 (3):790-798. doi:10.1152/japplphysiol.00310.2018

Claassen JA, Diaz-Arrastia R, Martin-Cook K, Levine BD, Zhang R (2009a) Altered cerebral hemodynamics in early Alzheimer disease: a pilot study using transcranial Doppler. J Alzheimers Dis 17 (3):621-629. doi:10.3233/jad-2009-1079

Claassen JA, Levine BD, Zhang R (2009b) Dynamic cerebral autoregulation during repeated squat-stand maneuvers. J Appl Physiol (1985) 106 (1):153-160. doi:10.1152/japplphysiol.90822.2008

Cornwell WK, 3rd, Tarumi T, Aengevaeren VL, Ayers C, Divanji P, Fu Q, Palmer D, Drazner MH, Meyer DM, Bethea BT, Hastings JL, Fujimoto N, Shibata S, Zhang R, Markham DW, Levine BD (2014) Effect of pulsatile and nonpulsatile flow on cerebral perfusion in patients with left ventricular assist devices. J Heart Lung Transplant 33 (12):1295-1303. doi:10.1016/j.healun.2014.08.013

de Heus RAA, de Jong DLK, Sanders ML, van Spijker GJ, Oudegeest-Sander MH, Hopman MT, Lawlor BA, Olde Rikkert MGM, Claassen J (2018) Dynamic Regulation of Cerebral Blood Flow in Patients With Alzheimer Disease. Hypertension 72 (1):139-150. doi:10.1161/hypertensionaha.118.10900

Diehl RR, Linden D, Lucke D, Berlit P (1995) Phase relationship between cerebral blood flow velocity and blood pressure. A clinical test of autoregulation. Stroke; a journal of cerebral circulation 26 (10):1801-1804. doi:papers3://publication/uuid/152C849F-AAF6-451F-A5D2-1E6E1CB38893

Ding K, Tarumi T, Tomoto T, McColloster M, Le T, Dieppa M, Diaz-Arrastia R, Bell K, Madden C, Cullum CM, Zhang R (2020) Impaired cerebral blood flow regulation in chronic traumatic brain injury. Brain Res 1743:146924. doi:10.1016/j.brainres.2020.146924

Drapeau A, Labrecque L, Imhoff S, Paquette M, Le Blanc O, Malenfant S, Brassard P (2019) Six weeks of high-intensity interval training to exhaustion attenuates dynamic cerebral autoregulation without influencing resting cerebral blood velocity in young fit men. Physiol Rep 7 (15):e14185. doi:10.14814/phy2.14185

Eames PJ, Potter JF, Panerai RB (2004) Influence of controlled breathing patterns on cerebrovascular autoregulation and cardiac baroreceptor sensitivity. Clin Sci (Lond) 106 (2):155-162. doi:10.1042/cs20030194

Elting JW, Aries MJ, van der Hoeven JH, Vroomen PC, Maurits NM (2014) Reproducibility and variability of dynamic cerebral autoregulation during passive cyclic leg raising. Med Eng Phys 36 (5):585-591. doi:10.1016/j.medengphy.2013.09.012

Favre ME, Serrador JM (2019) Sex differences in cerebral autoregulation are unaffected by menstrual cycle phase in young, healthy women. Am J Physiol Heart Circ Physiol 316 (4):H920-h933. doi:10.1152/ajpheart.00474.2018

Gommer ED, Shijaku E, Mess WH, Reulen JP (2010) Dynamic cerebral autoregulation: different signal processing methods without influence on results and reproducibility. Med Biol Eng Comput 48 (12):1243-1250. doi:10.1007/s11517-010-0706-y

Gong X, Liu J, Dong P, Zhang P, Li N, Zhao X, Wang Y (2013) Assessment of dynamic cerebral autoregulation in patients with basilar artery stenosis. PLoS One 8 (10):e77802. doi:10.1371/journal.pone.0077802

Hamner JW, Cohen MA, Mukai S, Lipsitz LA, Taylor JA (2004) Spectral indices of human cerebral blood flow control: responses to augmented blood pressure oscillations. J Physiol 559 (Pt 3):965-973. doi:10.1113/jphysiol.2004.066969

Hamner JW, Ishibashi K, Tan CO (2019) Revisiting human cerebral blood flow responses to augmented blood pressure oscillations. J Physiol 597 (6):1553-1564. doi:10.1113/jp277321

Hamner JW, Tan CO (2014) Relative contributions of sympathetic, cholinergic, and myogenic mechanisms to cerebral autoregulation. Stroke 45 (6):1771-1777. doi:10.1161/strokeaha.114.005293

Hamner JW, Tan CO, Lee K, Cohen MA, Taylor JA (2010) Sympathetic control of the cerebral vasculature in humans. Stroke 41 (1):102-109. doi:10.1161/strokeaha.109.557132

Hamner JW, Tan CO, Tzeng YC, Taylor JA (2012) Cholinergic control of the cerebral vasculature in humans. J Physiol 590 (24):6343-6352. doi:10.1113/jphysiol.2012.245100

Hughson RL, Edwards MR, O'Leary DD, Shoemaker JK (2001) Critical analysis of cerebrovascular autoregulation during repeated head-up tilt. Stroke; a journal of cerebral circulation 32 (10):2403-2408. doi:papers3://publication/uuid/BB87D857-15A0-41F0-B2D6-329073F0C439

Janzarik WG, Ehlers E, Ehmann R, Gerds TA, Schork J, Mayer S, Gabriel B, Weiller C, Prömpeler H, Reinhard M (2014) Dynamic cerebral autoregulation in pregnancy and the risk of preeclampsia. Hypertension 63 (1):161-166. doi:10.1161/hypertensionaha.113.01667

Janzarik WG, Gerber AK, Markfeld-Erol F, Sommerlade L, Allignol A, Reinhard M (2018) No long-term impairment of cerebral autoregulation after preeclampsia. Pregnancy Hypertens 13:171-173. doi:10.1016/j.preghy.2018.06.009

Janzarik WG, Jacob J, Katagis E, Markfeld-Erol F, Sommerlade L, Wuttke M, Reinhard M (2019) Preeclampsia postpartum: Impairment of cerebral autoregulation and reversible cerebral hyperperfusion. Pregnancy Hypertens 17:121-126. doi:10.1016/j.preghy.2019.05.019

Junejo RT, Braz ID, Lucas SJ, van Lieshout JJ, Phillips AA, Lip GY, Fisher JP (2020) Neurovascular coupling and cerebral autoregulation in atrial fibrillation. J Cereb Blood Flow Metab 40 (8):1647-1657. doi:10.1177/0271678x19870770

Katsogridakis E, Bush G, Fan L, Birch AA, Simpson DM, Allen R, Potter JF, Panerai RB (2012) Random perturbations of arterial blood pressure for the assessment of dynamic cerebral autoregulation. Physiol Meas 33 (2):103-116. doi:10.1088/0967-3334/33/2/103

Klein T, Bailey TG, Wollseiffen P, Schneider S, Askew CD (2020) The effect of age on cerebral blood flow responses during repeated and sustained stand to sit transitions. Physiol Rep 8 (9):e14421. doi:10.14814/phy2.14421

Kwan J, Lunt M, Jenkinson D (2004) Assessing dynamic cerebral autoregulation after stroke using a novel technique of combining transcranial Doppler ultrasonography and rhythmic handgrip. Blood Press Monit 9 (1):3-8. doi:10.1097/00126097-200402000-00002

Labrecque L, Burma JS, Roy MA, Smirl JD, Brassard P (2022) Reproducibility and diurnal variation of the directional sensitivity of the cerebral pressure-flow relationship in men and women. J Appl Physiol (1985) 132 (1):154-166. doi:10.1152/japplphysiol.00653.2021

Labrecque L, Drapeau A, Rahimaly K, Imhoff S, Brassard P (2021a) Dynamic cerebral autoregulation and cerebrovascular carbon dioxide reactivity in middle and posterior cerebral arteries in young endurance-trained women. Journal of Applied Physiology 130 (6):1724-1735. doi:10.1152/japplphysiol.00963.2020

Labrecque L, Rahimaly K, Imhoff S, Paquette M, Le Blanc O, Malenfant S, Drapeau A, Smirl JD, Bailey DM, Brassard P (2019) Dynamic cerebral autoregulation is attenuated in young fit women. Physiol Rep 7 (2):e13984. doi:10.14814/phy2.13984

Labrecque L, Rahimaly K, Imhoff S, Paquette M, Le Blanc O, Malenfant S, Lucas SJE, Bailey DM, Smirl JD, Brassard P (2017) Diminished dynamic cerebral autoregulatory capacity with forced oscillations in mean arterial pressure with elevated cardiorespiratory fitness. Physiol Rep 5 (21). doi:10.14814/phy2.13486

Labrecque L, Smirl JD, Brassard P (2021b) Utilization of the repeated squat-stand model for studying the directional sensitivity of the cerebral pressure-flow relationship. Journal of Applied Physiology 131 (3):927-936. doi:10.1152/japplphysiol.00269.2021

Lagi A, Laffi G, Cencetti S, Barletta G, Foschi M, Vizzutti F, Bandinelli R, Pantaleo P, Tosti Guerra C, Gentilini P, La Villa G (2002) Impaired sympathetic regulation of cerebral blood flow in patients with cirrhosis of the liver. Clin Sci (Lond) 103 (1):43-51. doi:10.1042/cs1030043

Lewis PM, Rosenfeld JV, Diehl RR, Mehdorn HM, Lang EW (2008) Phase shift and correlation coefficient measurement of cerebral autoregulation during deep breathing in traumatic brain injury (TBI). Acta Neurochir (Wien) 150 (2):139-146; discussion 146-137. doi:10.1007/s00701-007-1447-z

Liu Y, Birch AA, Allen R (2003) Dynamic cerebral autoregulation assessment using an ARX model: comparative study using step response and phase shift analysis. Med Eng Phys 25 (8):647-653. doi:10.1016/s1350-4533(03)00015-8

Lucas SJ, Lewis NC, Sikken EL, Thomas KN, Ainslie PN (2013) Slow breathing as a means to improve orthostatic tolerance: a randomized sham-controlled trial. J Appl Physiol (1985) 115 (2):202-211. doi:10.1152/japplphysiol.00128.2013

Malenfant S, Brassard P, Paquette M, Le Blanc O, Chouinard A, Nadeau V, Allan PD, Tzeng YC, Simard S, Bonnet S, Provencher S (2017) Compromised cerebrovascular regulation and cerebral oxygenation in pulmonary arterial hypertension. J Am Heart Assoc 6 (10). doi:10.1161/jaha.117.006126

Marthol H, Brown CM, Zikeli U, Ziegler D, Dimitrov N, Baltadzhieva R, Hilz MJ (2006) Altered cerebral regulation in type 2 diabetic patients with cardiac autonomic neuropathy. Diabetologia 49 (10):2481-2487. doi:10.1007/s00125-006-0368-3

Maxwell JD, Bannell DJ, Brislane A, Carter SE, Miller GD, Roberts KA, Hopkins ND, Low DA, Carter HH, Thompson A, Claassen JAHR, Thijssen DHJ, Jones H (2022) The impact of age, sex, cardio-respiratory fitness, and cardiovascular disease risk on dynamic cerebral autoregulation and baroreflex sensitivity. European Journal of Applied Physiology 122 (6):1531-1541. doi:10.1007/s00421-022-04933-3

Maxwell JD, France M, Finnigan LEM, Carter HH, Thijssen DHJ, Jones H (2021) Can exercise training enhance the repeated remote ischaemic preconditioning stimulus on peripheral and cerebrovascular function in high-risk individuals? Eur J Appl Physiol 121 (4):1167-1178. doi:10.1007/s00421-020-04580-6

Mense L, Reimann M, Rüdiger H, Gahn G, Reichmann H, Hentschel H, Ziemssen T (2010) Autonomic function and cerebral autoregulation in patients undergoing carotid endarterectomy. Circ J 74 (10):2139-2145. doi:10.1253/circj.cj-10-0365

Mol A, Meskers CGM, Sanders ML, Müller M, Maier AB, van Wezel RJA, Claassen J, Elting JWJ (2021) Cerebral autoregulation assessed by near-infrared spectroscopy: validation using transcranial Doppler in patients with controlled hypertension, cognitive impairment and controls. Eur J Appl Physiol 121 (8):2165-2176. doi:10.1007/s00421-021-04681-w

Newel KT, Burma JS, Carere J, Kennedy CM, Smirl JD (2022) Does oscillation size matter? Impact of added resistance on the cerebral pressure-flow Relationship in females and males. Physiol Rep 10 (10):e15278. doi:10.14814/phy2.15278

Oudegeest-Sander MH, van Beek AH, Abbink K, Olde Rikkert MG, Hopman MT, Claassen JA (2014) Assessment of dynamic cerebral autoregulation and cerebrovascular CO2 reactivity in ageing by measurements of cerebral blood flow and cortical oxygenation. Exp Physiol 99 (3):586-598. doi:10.1113/expphysiol.2013.076455

Panerai RB, Barnes SC, Nath M, Ball N, Robinson TG, Haunton VJ (2018) Directional sensitivity of dynamic cerebral autoregulation in squat-stand maneuvers. Am J Physiol Regul Integr Comp Physiol 315 (4):R730-r740. doi:10.1152/ajpregu.00010.2018

Panerai RB, Batterham A, Robinson TG, Haunton VJ (2021) Determinants of cerebral blood flow velocity change during squat-stand maneuvers. Am J Physiol Regul Integr Comp Physiol 320 (4):R452-r466. doi:10.1152/ajpregu.00291.2020

Panerai RB, Eames PJ, Potter JF (2003) Variability of time-domain indices of dynamic cerebral autoregulation. Physiol Meas 24 (2):367-381. doi:10.1088/0967-3334/24/2/312

Perry BG, Cotter JD, Korad S, Lark S, Labrecque L, Brassard P, Paquette M, Le Blanc O, Lucas SJE (2019) Implications of habitual endurance and resistance exercise for dynamic cerebral autoregulation. Exp Physiol 104 (12):1780-1789. doi:10.1113/ep087675

Purkayastha S, Maffuid K, Zhu X, Zhang R, Raven PB (2018) The influence of the carotid baroreflex on dynamic regulation of cerebral blood flow and cerebral tissue oxygenation in humans at rest and during exercise. Eur J Appl Physiol 118 (5):959-969. doi:10.1007/s00421-018-3831-1

Querido JS, Ainslie PN, Foster GE, Henderson WR, Halliwill JR, Ayas NT, Sheel AW (2013) Dynamic cerebral autoregulation during and following acute hypoxia: role of carbon dioxide. J Appl Physiol (1985) 114 (9):1183-1190. doi:10.1152/japplphysiol.00024.2013

Reed EL, Worley ML, Kueck PJ, Pietrafasa LD, Schlader ZJ, Johnson BD (2022) Cerebral vascular function following the acute consumption of caffeinated artificially- and sugar

sweetened soft drinks in healthy adults. Front Hum Neurosci 16:1063273. doi:10.3389/fnhum.2022.1063273
Reinhard M, Gerds TA, Grabiak D, Zimmermann PR, Roth M, Guschlbauer B, Timmer J, Czosnyka M, Weiller C, Hetzel A (2008a) Cerebral dysautoregulation and the risk of ischemic events in occlusive carotid artery disease. J Neurol 255 (8):1182-1189. doi:10.1007/s00415-008-0865-z
Reinhard M, Hetzel A, Lauk M, Lücking CH (2001) Dynamic cerebral autoregulation testing as a diagnostic tool in patients with carotid artery stenosis. Neurol Res 23 (1):55-63. doi:10.1179/016164101101198299
Reinhard M, Lorenz L, Sommerlade L, Allignol A, Urbach H, Weiller C, Egger K (2019) Impaired dynamic cerebral autoregulation in patients with cerebral amyloid angiopathy. Brain Res 1717:60-65. doi:10.1016/j.brainres.2019.04.014
Reinhard M, Müller T, Guschlbauer B, Timmer J, Hetzel A (2003a) Dynamic cerebral autoregulation and collateral flow patterns in patients with severe carotid stenosis or occlusion. Ultrasound Med Biol 29 (8):1105-1113. doi:10.1016/s0301-5629(03)00954-2
Reinhard M, Müller T, Guschlbauer B, Timmer J, Hetzel A (2003b) Transfer function analysis for clinical evaluation of dynamic cerebral autoregulation--a comparison between spontaneous and respiratory-induced oscillations. Physiol Meas 24 (1):27-43. doi:10.1088/0967-3334/24/1/303
Reinhard M, Müller T, Roth M, Guschlbauer B, Timmer J, Hetzel A (2003c) Bilateral severe carotid artery stenosis or occlusion - cerebral autoregulation dynamics and collateral flow patterns. Acta Neurochir (Wien) 145 (12):1053-1059; discussion 1059-1060. doi:10.1007/s00701-003-0137-8
Reinhard M, Roth M, Guschlbauer B, Czosnyka M, Timmer J, Weiller C, Hetzel A (2011) The course of dynamic cerebral autoregulation during cervical internal carotid artery occlusion. Neurol Res 33 (9):921-926. doi:10.1179/1743132811y.0000000022
Reinhard M, Schork J, Allignol A, Weiller C, Kaube H (2012) Cerebellar and cerebral autoregulation in migraine. Stroke 43 (4):987-993. doi:10.1161/strokeaha.111.644674
Reinhard M, Waldkircher Z, Timmer J, Weiller C, Hetzel A (2008b) Cerebellar autoregulation dynamics in humans. J Cereb Blood Flow Metab 28 (9):1605-1612. doi:10.1038/jcbfm.2008.48
Rickards CA, Sprick JD, Colby HB, Kay VL, Tzeng YC (2015) Coupling between arterial pressure, cerebral blood velocity, and cerebral tissue oxygenation with spontaneous and forced oscillations. Physiol Meas 36 (4):785-801. doi:10.1088/0967-3334/36/4/785
Rodrigues GD, Nobrega A, Soares P (2023) Respiratory training in older women: Unravelling central and peripheral hemodynamic slow oscillatory patterns. Exp Gerontol 172:112058. doi:10.1016/j.exger.2022.112058
Roy MA, Labrecque L, Perry BG, Korad S, Smirl JD, Brassard P (2022) Directional sensitivity of the cerebral pressure-flow relationship in young healthy individuals trained in endurance and resistance exercise. Exp Physiol 107 (4):299-311. doi:10.1113/ep090159
Saleem S, Teal PD, Kleijn WB, O'Donnell T, Witter T, Tzeng YC (2015) Non-Linear Characterisation of Cerebral Pressure-Flow Dynamics in Humans. PLoS One 10 (9):e0139470. doi:10.1371/journal.pone.0139470
Ševerdija EE, Gommer ED, Weerwind PW, Reulen JP, Mess WH, Maessen JG (2015) Assessment of dynamic cerebral autoregulation and cerebral carbon dioxide reactivity

during normothermic cardiopulmonary bypass. Med Biol Eng Comput 53 (3):195-203. doi:10.1007/s11517-014-1225-z
Smail OJ, Clarke DJ, Al-Alem Q, Wallis W, Barker AR, Smirl JD, Bond B (2023) Resistance exercise acutely elevates dynamic cerebral autoregulation gain. Physiol Rep 11 (8):e15676. doi:10.14814/phy2.15676
Smirl JD, Haykowsky MJ, Nelson MD, Tzeng YC, Marsden KR, Jones H, Ainslie PN (2014a) Relationship between cerebral blood flow and blood pressure in long-term heart transplant recipients. Hypertension 64 (6):1314-1320. doi:10.1161/hypertensionaha.114.04236
Smirl JD, Hoffman K, Tzeng YC, Hansen A, Ainslie PN (2015) Methodological comparison of active- and passive-driven oscillations in blood pressure; implications for the assessment of cerebral pressure-flow relationships. J Appl Physiol (1985) 119 (5):487-501. doi:10.1152/japplphysiol.00264.2015
Smirl JD, Hoffman K, Tzeng YC, Hansen A, Ainslie PN (2016) Relationship between blood pressure and cerebral blood flow during supine cycling: influence of aging. J Appl Physiol (1985) 120 (5):552-563. doi:10.1152/japplphysiol.00667.2015
Smirl JD, Lucas SJ, Lewis NC, duManoir GR, Smith KJ, Bakker A, Basnyat AS, Ainslie PN (2014b) Cerebral pressure-flow relationship in lowlanders and natives at high altitude. J Cereb Blood Flow Metab 34 (2):248-257. doi:10.1038/jcbfm.2013.178
Smirl JD, Peacock D, Burma JS, Wright AD, Bouliane KJ, Dierijck J, Kennefick M, Wallace C, van Donkelaar P (2022) An acute bout of controlled subconcussive impacts can alter dynamic cerebral autoregulation indices: a preliminary investigation. Eur J Appl Physiol 122 (4):1059-1070. doi:10.1007/s00421-022-04908-4
Smirl JD, Tzeng YC, Monteleone BJ, Ainslie PN (2014c) Influence of cerebrovascular resistance on the dynamic relationship between blood pressure and cerebral blood flow in humans. J Appl Physiol (1985) 116 (12):1614-1622. doi:10.1152/japplphysiol.01266.2013
Smirl JD, Wright AD, Ainslie PN, Tzeng YC, van Donkelaar P (2018) Differential systolic and diastolic regulation of the cerebral pressure-flow relationship during squat-stand manoeuvres. Acta Neurochir Suppl 126:263-268. doi:10.1007/978-3-319-65798-1_52
Sommerlade L, Schelter B, Timmer J, Reinhard M (2012) Grading of dynamic cerebral autoregulation without blood pressure recordings: a simple Doppler-based method. Ultrasound Med Biol 38 (9):1546-1551. doi:10.1016/j.ultrasmedbio.2012.05.003
Sprick JD, Jones T, Jeong J, DaCosta D, Park J (2022) Dynamic cerebral autoregulation is intact in chronic kidney disease. Physiol Rep 10 (21):e15495. doi:10.14814/phy2.15495
Stefanidis KB, Askew CD, Klein T, Lagopoulos J, Summers MJ (2019) Healthy aging affects cerebrovascular reactivity and pressure-flow responses, but not neurovascular coupling: A cross-sectional study. PLoS One 14 (5):e0217082. doi:10.1371/journal.pone.0217082
Stefanidis KB, Isbel B, Klein T, Lagopoulos J, Askew CD, Summers MJ (2020) Reduced cerebral pressure-flow responses are associated with electrophysiological markers of attention in healthy older adults. J Clin Neurosci 81:167-172. doi:10.1016/j.jocn.2020.09.034
Stok WJ, Karemaker JM, Berecki-Gisolf J, Immink RV, van Lieshout JJ (2019) Slow sinusoidal tilt movements demonstrate the contribution to orthostatic tolerance of cerebrospinal fluid movement to and from the spinal dural space. Physiol Rep 7 (4):e14001. doi:10.14814/phy2.14001

Tan CO (2012) Defining the characteristic relationship between arterial pressure and cerebral flow. J Appl Physiol (1985) 113 (8):1194-1200. doi:10.1152/japplphysiol.00783.2012
Tan CO, Hamner JW, Taylor JA (2013) The role of myogenic mechanisms in human cerebrovascular regulation. J Physiol 591 (20):5095-5105. doi:10.1113/jphysiol.2013.259747
Tarumi T, Dunsky DI, Khan MA, Liu J, Hill C, Armstrong K, Martin-Cook K, Cullum CM, Zhang R (2014) Dynamic cerebral autoregulation and tissue oxygenation in amnestic mild cognitive impairment. J Alzheimers Dis 41 (3):765-778. doi:10.3233/jad-132018
Tarumi T, Harris TS, Hill C, German Z, Riley J, Turner M, Womack KB, Kerwin DR, Monson NL, Stowe AM, Mathews D, Cullum CM, Zhang R (2015) Amyloid burden and sleep blood pressure in amnestic mild cognitive impairment. Neurology 85 (22):1922-1929. doi:10.1212/wnl.0000000000002167
Tomoto T, Repshas J, Zhang R, Tarumi T (2021) Midlife aerobic exercise and dynamic cerebral autoregulation: associations with baroreflex sensitivity and central arterial stiffness. J Appl Physiol (1985) 131 (5):1599-1612. doi:10.1152/japplphysiol.00243.2021
Tutaj M, Brown CM, Brys M, Marthol H, Hecht MJ, Dutsch M, Michelson G, Hilz MJ (2004) Dynamic cerebral autoregulation is impaired in glaucoma. J Neurol Sci 220 (1-2):49-54. doi:10.1016/j.jns.2004.02.002
Tzeng YC, Ainslie PN, Cooke WH, Peebles KC, Willie CK, MacRae BA, Smirl JD, Horsman HM, Rickards CA (2012) Assessment of cerebral autoregulation: the quandary of quantification. Am J Physiol Heart Circ Physiol 303 (6):H658-671. doi:10.1152/ajpheart.00328.2012
Tzeng YC, Chan GS, Willie CK, Ainslie PN (2011) Determinants of human cerebral pressure-flow velocity relationships: new insights from vascular modelling and Ca^{2+} channel blockade. J Physiol 589 (Pt 13):3263-3274. doi:10.1113/jphysiol.2011.206953
van Beek AH, Lagro J, Olde-Rikkert MG, Zhang R, Claassen JA (2012) Oscillations in cerebral blood flow and cortical oxygenation in Alzheimer's disease. Neurobiol Aging 33 (2):428.e421-431. doi:10.1016/j.neurobiolaging.2010.11.016
van Beek AH, Olde Rikkert MG, Pasman JW, Hopman MT, Claassen JA (2010) Dynamic cerebral autoregulation in the old using a repeated sit-stand maneuver. Ultrasound Med Biol 36 (2):192-201. doi:10.1016/j.ultrasmedbio.2009.10.011
Wallis WEG, Al-Alem Q, Lorimer H, Smail OJ, Williams GKR, Bond B (2023) The acute influence of amateur boxing on dynamic cerebral autoregulation and cerebrovascular reactivity to carbon dioxide. Eur J Appl Physiol. doi:10.1007/s00421-023-05324-y
Worley ML, Reed EL, Chapman CL, Kueck P, Seymour L, Fitts T, Zazulak H, Schlader ZJ, Johnson BD (2023) Acute beetroot juice consumption does not alter cerebral autoregulation or cardiovagal baroreflex sensitivity during lower-body negative pressure in healthy adults. Front Hum Neurosci 17:1115355. doi:10.3389/fnhum.2023.1115355
Wright AD, Smirl JD, Bryk K, Fraser S, Jakovac M, van Donkelaar P (2018a) Cerebral autoregulation is disrupted following a season of contact sports participation. Front Neurol 9:868. doi:10.3389/fneur.2018.00868
Wright AD, Smirl JD, Bryk K, Fraser S, Jakovac M, van Donkelaar P (2018b) Sport-related concussion alters indices of dynamic cerebral autoregulation. Front Neurol 9:196. doi:10.3389/fneur.2018.00196

Wright AD, Smirl JD, Bryk K, van Donkelaar P (2018c) Systolic and diastolic regulation of the cerebral pressure-flow relationship differentially affected by acute sport-related concussion. Acta Neurochir Suppl 126:303-308. doi:10.1007/978-3-319-65798-1_59

Xing CY, Tarumi T, Meijers RL, Turner M, Repshas J, Xiong L, Ding K, Vongpatanasin W, Yuan LJ, Zhang R (2017) Arterial Pressure, Heart Rate, and Cerebral Hemodynamics Across the Adult Life Span. Hypertension 69 (4):712-720. doi:10.1161/hypertensionaha.116.08986

Yoshida H, Hamner JW, Ishibashi K, Tan CO (2018) Relative contributions of systemic hemodynamic variables to cerebral autoregulation during orthostatic stress. Journal of Applied Physiology 124 (2):321-329. doi:10.1152/japplphysiol.00700.2017

Zhang R, Zuckerman JH, Iwasaki K, Wilson TE, Crandall CG, Levine BD (2002) Autonomic neural control of dynamic cerebral autoregulation in humans. Circulation 106 (14):1814-1820. doi:10.1161/01.cir.0000031798.07790.fe

Supplemental B: Included Studies and Risk of Bias Scoring

Authors	Title	Country	MINORS Score	SIGN ROB
Aaron et al.	Cerebrovascular Neuroprotection after Acute Concussion in Adolescents	United States	16	(-)
Aaslid et al.	Asymmetric dynamic cerebral autoregulatory response to cyclic stimuli	United States	17	(-)
Abbariki et al.	Influence of high-intensity interval training to exhaustion on the directional sensitivity of the cerebral pressure-flow relationship in young endurance-trained men	Canada	20	(++)
Aengevaeren et al.	Cardiac baroreflex function and dynamic cerebral autoregulation in elderly Masters athletes	United States	19	(+)
Anderson et al.	Peaks and valleys: oscillatory cerebral blood flow at high altitude protects cerebral tissue oxygenation	United States	20	(+)
Barnes et al.	How many squat-stand manoeuvres to assess dynamic cerebral autoregulation?	United Kingdom	18	(++)
Barnes et al.	Random squat/stand maneuvers: a novel approach for assessment of dynamic cerebral autoregulation?	United Kingdom	18	(+)
Barnes et al.	The cerebrocardiovascular response to periodic squat-stand maneuvers in healthy subjects: a time-domain analysis	United Kingdom	17	(-)
Birch et al.	Assessment of autoregulation by means of periodic changes in blood pressure	United Kingdom	20	(+)
Birch et al.	The repeatability of cerebral autoregulation assessment using sinusoidal lower body negative pressure	United Kingdom	19	(-)
Brassard et al.	Evidence for hysteresis in the cerebral pressure-flow relationship in healthy men	Canada	20	(+)
Brothers et al.	Effects of heat stress on dynamic cerebral autoregulation during large fluctuations in arterial blood pressure	United States	21	(++)
Brown et al.	A simple deep breathing test reveals altered cerebral autoregulation in type 2 diabetic patients	Germany	18	(+)
Brown et al.	Cerebral autoregulation is compromised during simulated fluctuations in gravitational stress	Germany	19	(+)
Burma et al.	Comparison of diurnal variation, anatomical location, and biological sex within spontaneous and driven dynamic cerebral autoregulation measures	Canada	23	(++)
Burma et al.	Dynamic cerebral autoregulation across the cardiac cycle during 8 hr of recovery from acute exercise	Canada	21	(++)
Burma et al.	Long-term heart transplant recipients: heart rate-related effects on augmented transfer function coherence during repeated squat-stand maneuvers in males	Canada	18	(+)
Burma et al.	What recording duration is required to provide physiologically valid and reliable dynamic cerebral autoregulation transfer functional analysis estimates?	Canada	20	(++)
Carter et al.	Regular walking breaks prevent the decline in cerebral blood flow associated with prolonged sitting	United Kingdom	21	(+)
Claassen et al.	Altered Cerebral Hemodynamics in Early Alzheimer Disease: A Pilot Study Using Transcranial Doppler	United States	18	(++)
Claassen et al.	Dynamic cerebral autoregulation during repeated squat-stand maneuvers	United States	20	(++)
Cornwell et al.	Effect of pulsatile and nonpulsatile flow on cerebral perfusion in patients with left ventricular assist devices	United States, Netherlands	21	(+)
de Heus et al.	Dynamic Regulation of Cerebral Blood Flow in Patients With Alzheimer Disease	Netherlands	20	(++)

Diehl et al.	Phase relationship between cerebral blood flow velocity and blood pressure. A clinical test of autoregulation	Germany	18	(-)
Ding et al.	Impaired cerebral blood flow regulation in chronic traumatic brain injury	United States	19	(+)
Drapeau et al.	Six weeks of high-intensity interval training to exhaustion attenuates dynamic cerebral autoregulation without influencing resting cerebral blood velocity in young fit men	Canada	19	(+)
Eames et al.	Influence of controlled breathing patterns on cerebrovascular autoregulation and cardiac baroreceptor sensitivity	United Kingdom	20	(+)
Elting et al.	Reproducibility and variability of dynamic cerebral autoregulation during passive cyclic leg raising	Netherlands	21	(+)
Favre et al.	Sex differences in cerebral autoregulation are unaffected by menstrual cycle phase in young, healthy women	United States	20	(++)
Gommer et al.	Dynamic cerebral autoregulation: different signal processing methods without influence on results and reproducibility	Netherlands	21	(-)
Gong et al.	Assessment of Dynamic Cerebral Autoregulation in Patients with Basilar Artery Stenosis	China	20	(-)
Hamner et al.	Cholinergic control of the cerebral vasculature in humans	United States	20	(+)
Hamner et al.	Relative contributions of sympathetic, cholinergic, and myogenic mechanisms to cerebral autoregulation	United States	21	(+)
Hamner et al.	Revisiting human cerebral blood flow responses to augmented blood pressure oscillations	United States	21	(++)
Hamner et al.	Spectral indices of human cerebral blood flow control: responses to augmented blood pressure oscillations	United States	21	(+)
Hamner et al.	Sympathetic control of the cerebral vasculature in humans	United States	21	(+)
Hughson et al.	Critical analysis of cerebrovascular autoregulation during repeated head-up tilt	Canada	19	(-)
Janzarik et al.	Dynamic cerebral autoregulation in pregnancy and the risk of preeclampsia	Germany	19	(+)
Janzarik et al.	No long-term impairment of cerebral autoregulation after preeclampsia	Germany	17	(-)
Janzarik et al.	Preeclampsia postpartum: Impairment of cerebral autoregulation and reversible cerebral hyperperfusion	Germany	18	(-)
Junejo et al.	Neurovascular coupling and cerebral autoregulation in atrial fibrillation	United Kingdom	23	(++)
Katsogridakis et al.	Random perturbations of arterial blood pressure for the assessment of dynamic cerebral autoregulation	United States	18	(-)
Klein et al.	The effect of age on cerebral blood flow responses during repeated and sustained stand to sit transitions	Australia	20	(+)
Kwan et al.	Assessing dynamic cerebral autoregulation after stroke using a novel technique of combining transcranial Doppler ultrasonography and rhythmic handgrip	United Kingdom	17	(-)
Labrecque et al.	Diminished dynamic cerebral autoregulatory capacity with forced oscillations in mean arterial pressure with elevated cardiorespiratory fitness	Canada	20	(++)
Labrecque et al.	Dynamic cerebral autoregulation and cerebrovascular carbon dioxide reactivity in middle and posterior cerebral arteries in young endurance-trained women	Canada	20	(++)
Labrecque et al.	Dynamic cerebral autoregulation is attenuated in young fit women	Canada	20	(++)
Labrecque et al.	Reproducibility and diurnal variation of the directional sensitivity of the cerebral pressure-flow relationship in men and women	Canada	19	(++)
Labrecque et al.	Utilization of the repeated squat-stand model for studying the directional sensitivity of the cerebral pressure-flow relationship	Canada	19	(++)

Lagi et al.	Impaired sympathetic regulation of cerebral blood flow in patients with cirrhosis of the liver	Italy	21	(+)
Lewis et al.	Phase shift and correlation coefficient measurement of cerebral autoregulation during deep breathing in traumatic brain injury (TBI)	Germany	20	(+)
Liu et al.	Dynamic cerebral autoregulation assessment using an ARX model: comparative study using step response and phase shift analysis	United Kingdom	17	(-)
Lucas et al.	Slow breathing as a means to improve orthostatic tolerance: a randomized sham-controlled trial	Canada	19	(++)
Malenfant et al.	Compromised Cerebrovascular Regulation and Cerebral Oxygenation in Pulmonary Arterial Hypertension	Canada	20	(+)
Marthol et al.	Altered cerebral regulation in type 2 diabetic patients with cardiac autonomic neuropathy	Germany	16	(-)
Maxwell et al.	Can exercise training enhance the repeated remote ischaemic preconditioning stimulus on peripheral and cerebrovascular function in high-risk individuals?	United Kingdom	22	(++)
Maxwell et al.	The impact of age, sex, cardio-respiratory fitness, and cardiovascular disease risk on dynamic cerebral autoregulation and baroreflex sensitivity	United Kingdom	20	(++)
Mense et al.	Autonomic function and cerebral autoregulation in patients undergoing carotid endarterectomy	Germany	17	(-)
Mol et al.	Cerebral autoregulation assessed by near-infrared spectroscopy: validation using transcranial Doppler in patients with controlled hypertension, cognitive impairment and controls	Netherlands	14	(-)
Newel et al.	Does oscillation size matter? Impact of added resistance on the cerebral pressure-flow Relationship in females and males	Canada	22	(+)
Oudegeest-Sander et al.	Assessment of dynamic cerebral autoregulation and cerebrovascular CO2 reactivity in ageing by measurements of cerebral blood flow and cortical oxygenation	Netherlands	20	(+)
Panerai et al.	Determinants of cerebral blood flow velocity change during squat-stand maneuvers	United Kingdom	20	(+)
Panerai et al.	Directional sensitivity of dynamic cerebral autoregulation in squat-stand maneuvers	United Kingdom	20	(+)
Panerai et al.	Variability of time-domain indices of dynamic cerebral autoregulation	United Kingdom	19	(+)
Perry et al.	Implications of habitual endurance and resistance exercise for dynamic cerebral autoregulation	Canada, New Zealand	20	(++)
Purkayastha et al.	The influence of the carotid baroreflex on dynamic regulation of cerebral blood flow and cerebral tissue oxygenation in humans at rest and during exercise	United States	21	(+)
Querido et al.	Dynamic cerebral autoregulation during and following acute hypoxia: role of carbon dioxide	Canada	20	(+)
Reed et al.	Cerebral vascular function following the acute consumption of caffeinated artificially- and sugar sweetened soft drinks in healthy adults	United States	21	(++)
Reinhard et al.	Bilateral severe carotid artery stenosis or occlusion - cerebral autoregulation dynamics and collateral flow patterns	Germany	14	(-)
Reinhard et al.	Cerebellar and cerebral autoregulation in Migraine	Germany	20	(+)
Reinhard et al.	Cerebellar autoregulation dynamics in humans	Germany	20	(+)
Reinhard et al.	Cerebral dysautoregulation and the risk of ischemic events in occlusive carotid artery disease	Germany	21	(+)
Reinhard et al.	Dynamic cerebral autoregulation and collateral flow patterns in patients with severe carotid stenosis or occlusion	Germany	18	(-)
Reinhard et al.	Dynamic cerebral autoregulation testing as a diagnostic tool in patients with carotid artery stenosis	Germany	21	(+)

Reinhard et al.	Impaired dynamic cerebral autoregulation in patients with cerebral amyloid angiopathy	Germany	18	(+)
Reinhard et al.	The course of dynamic cerebral autoregulation during cervical internal carotid artery occlusion	Germany	16	(-)
Reinhard et al.	Transfer function analysis for clinical evaluation of dynamic cerebral autoregulation--a comparison between spontaneous and respiratory-induced oscillations	Germany	17	(+)
Rickards et al.	Coupling between arterial pressure, cerebral blood velocity, and cerebral tissue oxygenation with spontaneous and forced oscillations	United States, New Zealand	20	(+)
Rodrigues et al.	Respiratory training in older women: Unravelling central and peripheral hemodynamic slow oscillatory patterns	Brazil	17	(+)
Roy et al.	Directional sensitivity of the cerebral pressure-flow relationship in young healthy individuals trained in endurance and resistance exercise	Canada	17	(++)
Saleem et al.	Non-Linear Characterisation of Cerebral Pressure-Flow Dynamics in Humans	New Zealand	21	(++)
Ševerdija et al.	Assessment of dynamic cerebral autoregulation and cerebral carbon dioxide reactivity during normothermic cardiopulmonary bypass	Netherlands	19	(-)
Smail et al.	Resistance exercise acutely elevates dynamic cerebral autoregulation gain.	United Kingdom	20	(++)
Smirl et al.	An acute bout of controlled subconcussive impacts can alter dynamic cerebral autoregulation indices: a preliminary investigation	Canada	20	(++)
Smirl et al.	Cerebral pressure-flow relationship in lowlanders and natives at high altitude	Canada, Nepal	18	(+)
Smirl et al.	Differential Systolic and Diastolic Regulation of the Cerebral Pressure-Flow Relationship During Squat-Stand Manoeuvres	Canada	19	(+)
Smirl et al.	Influence of cerebrovascular resistance on the dynamic relationship between blood pressure and cerebral blood flow in humans	Canada	21	(+)
Smirl et al.	Methodological comparison of active- and passive-driven oscillations in blood pressure; implications for the assessment of cerebral pressure-flow relationships	Canada	21	(++)
Smirl et al.	Relationship between blood pressure and cerebral blood flow during supine cycling: influence of aging	Canada	19	(+)
Smirl et al.	Relationship between cerebral blood flow and blood pressure in long-term heart transplant recipients	Canada, United Kingdom	21	(++)
Sommerlade et al.	Grading of dynamic cerebral autoregulation without blood pressure recordings: a simple Doppler-based method	Germany	19	(-)
Sprick et al.	Dynamic cerebral autoregulation is intact in chronic kidney disease	United States	19	(+)
Stefanidis et al.	Healthy aging affects cerebrovascular reactivity and pressure-flow responses, but not neurovascular coupling: A cross-sectional study	Australia	19	(+)
Stefanidis et al.	Reduced cerebral pressure-flow responses are associated with electrophysiological markers of attention in healthy older adults	Australia	20	(+)
Stok et al.	Slow sinusoidal tilt movements demonstrate the contribution to orthostatic tolerance of cerebrospinal fluid movement to and from the spinal dural space	Netherlands	21	(-)
Tan et al.	Defining the characteristic relationship between arterial pressure and cerebral flow	United States	20	(+)
Tan et al.	The role of myogenic mechanisms in human cerebrovascular regulation	United States	21	(+)
Tarumi et al.	Amyloid burden and sleep blood pressure in amnestic mild cognitive impairment	United States	19	(+)
Tarumi et al.	Dynamic cerebral autoregulation and tissue oxygenation in amnestic mild cognitive impairment	United States	21	(++)

Tomoto et al.	Midlife aerobic exercise and dynamic cerebral autoregulation: associations with baroreflex sensitivity and central arterial stiffness	United States	20	(+)
Tutaj et al.	Dynamic cerebral autoregulation is impaired in glaucoma	Germany	20	(+)
Tzeng et al.	Assessment of cerebral autoregulation: the quandary of quantification	New Zealand, United States, United Kingdom	19	(++)
Tzeng et al.	Determinants of human cerebral pressure-flow velocity relationships: new insights from vascular modelling and Ca2+ channel blockade	New Zealand	20	(+)
van Beek et al.	Dynamic cerebral autoregulation in the old using a repeated sit-stand maneuver	Netherlands	21	(+)
van Beek et al.	Oscillations in cerebral blood flow and cortical oxygenation in Alzheimer's disease	Netherlands	19	(++)
Wallis et al.	The acute infuence of amateur boxing on dynamic cerebral autoregulation and cerebrovascular reactivity to carbon dioxide	United Kingdom	22	(+)
Worley et al.	Acute beetroot juice consumption does not alter cerebral autoregulation or cardiovagal baroreflex sensitivity during lower-body negative pressure in healthy adults	United States	21	(++)
Wright et al.	Cerebral autoregulation is disrupted following a season of contact sports participation	Canada	20	(+)
Wright et al.	Sport-related concussion alters indices of Dynamic cerebral autoregulation	Canada	20	(+)
Wright et al.	Systolic and Diastolic Regulation of the Cerebral Pressure-Flow Relationship Differentially Affected by Acute Sport-Related Concussion	Canada	20	(+)
Xing et al.	Arterial Pressure, Heart Rate, and Cerebral Hemodynamics across the Adult Life Span	United States	21	(+)
Yoshida et al.	Relative contributions of systemic hemodynamic variables to cerebral autoregulation during orthostatic stress	United States	21	(++)
Zhang et al.	Autonomic neural control of dynamic cerebral autoregulation in humans	United States	21	(+)

Supplemental C: Study Characteristics and Demographics.

Authors	Sex	Age	Vessel	Driven Method	Frequency	Outcomes
Aaron et al.	57 Total (30F and 27M)<br>Controls: 29 (16F and 13M)<br>Concussion: 28 (14F and 14M)	Controls: 18.2 ± 2.3<br><br>Concussion: 17.4 ± 2.1	MCA	Deep Breathing	NR	PPR (Falling Slope, Rising Slope, Autoregulatory Slope, Autoregulatory Range)
Aaslid et al.	24 Total (NR)<br>TBI: 14 (3F and 11M)<br>Control: 10 (NR)	TBI: 32 (r: 12-63)<br>Control: 34 (r: 27-56)	MCA	Leg Cuff Oscillations	0.033	Autoregulatory Gain Up = delta Critical Closing Pressure Up / delta Arterial Blood Pressure Up<br>Autoregulatory Gain Down = delta Critical Closing Pressure Up / delta Arterial Blood Pressure Up
Abbariki et al.	18 Total (0F and 18M)	27 ± 5	MCA	Squat-Stand manevuers	0.05, 0.10	Hysteresis (absolute delta MCAv, absolute delta MAP, time delta MCAv, time delta MAP, delta MCAv time / delta MAP time)
Aengevaeren et al.	23 Total (8F and 15M)<br>Master Athletes: 11 (3F and 8M)<br>Sedentary Elderly: 12 (5F and 7M)	Master Athletes: 73 ± 6<br>Sedentary Elderly: 71 ± 6	MCA	Sit-to-Stand	0.05	TFA (PSD, coherence, gain, nGain, phase) - band averages in VLF (0.02-0.07 Hz), LF (0.07-0.20 Hz), HF (0.20-0.35 Hz).
Anderson et al.	8 Total (4F and 4M)	30.1 ± 7.6	MCA	Oscillatory LBNP	0.10	TFA (PSD)
Barnes et al.	20 Total (4F and 16M)	22.2 ± 2.1	MCA	Squat-Stand manevuers	0.05	TFA (coherence, phase, gain) and ARI
Barnes et al.	29 Total (10F and 19M)	23.0 ± 4.9	MCA	Squat-Stand manevuers	0.05	TFA (coherence, gain, phase)<br>Autoregulatory Index
Barnes et al.	29 Total (10F and 19M)	23.0 ± 4.9	MCA	Squat-Stand manevuers	0.05	Time-domain: Physiological contributions to CBv
Birch et al.	5 Total (1F and 4M)	40 (r: 35-51)	MCA	Oscillatory LBNP	0.083	TFA (phase)
Birch et al.	14 Total (7F and 7M)	25 (r: 18-38)	MCA	Squat-Stand manevuers	0.10	TFA (phase)
Brassard et al.	58 Total (0F and 58M)	29 (r: 20-74)	MCA	Squat-Stand manevuers	0.05, 0.10	Hysteresis (absolute delta MCAv, absolute delta MA, delta MCAv hyper vs. hypo)
Brothers et al.	9 Total (7F and 2M)	37 ± 12	MCA	Oscillatory LBNP	0.05, 0.10	TFA (coherence, gain, phase)
Brown et al.	21 Total (6F and 15M)<br>Controls: 11 (4F and 7M)<br>Type II Diabetes: 10 (2F and 8M)	Controls: 58 ± 2<br>Type II Diabetes: 54 ± 3	MCA	Deep Breathing	0.1	TFA (PSD, gain, phase)
Brown et al.	16 Total (8F and 8M)	27 ± 1 (r: 25-30)	MCA	Oscillatory LBNP	0.10, 0.20	TFA (PSD, coherence, gain, phase)
Burma et al.	16 Total (8F and 8M)	Total: 25 ± 4<br>Female: 24 ± 3<br>Male: 25 ± 5	MCA, PCA	Squat-Stand manevuers	0.05, 0.10	TFA (PSD, coherence, gain, nGain, phase)
Burma et al.	9 Total (2F and 7M)	26 ± 5	MCA, PCA	Squat-Stand manevuers	0.05, 0.10	TFA (PSD, coherence, gain, nGain, phase)
Burma et al.	40 Total (0F and 40M)<br>Donor Matched Control: 16 (0F	Donor Matched Control: 26.6 ±	MCA	Squat-Stand manevuers	0.05, 0.10	TFA (PSD, coherence)

	and 16M)<br>Age Matched Control: 16 (0F and 16M)<br>Heart Transplant Recipient: 8 (0F and 8M)	4.3<br>Age Matched Control: 64.5 ± 6.3<br>Heart Transplant Recipient: 62.0 ± 8.3				
Burma et al.	70 Total (17F and 53M)	26 ± 5 (r: 20-39)	MCA, PCA	Squat-Stand manevuers	0.05, 0.10	TFA (PSD, coherence, gain, nGain, phase)
Carter et al.	15 Total (5F and 10M)	35.8 ± 10.2	MCA	Squat-Stand manevuers	0.10	TFA (phase and gain) - band averages in VLF (0.02-0.07 Hz), LF (0.07-0.20 Hz), HF (0.20-0.50 Hz)
Claassen et al.	17 Total (10F and 7M)<br>Controls: 8 (4F and 4M)<br>Alzheimer's: 9 (6F and 3M)	18 Total (10F and 7M)<br><br>Controls: 8 (4F and 4M)<br><br>Alzheimer's: 9 (6F and 3M)	MCA	Squat-Stand manevuers	0.025, 0.05, 0.10	TFA (PSD, coherence, gain, phase)
Claassen et al.	8 Total (4F and 4M)	30 ± 4	MCA	Squat-Stand manevuers	0.025, 0.05, 0.10	TFA (coherence, gain, phase)+P49
Cornwell et al.	24 Total (3F and 21M)<br>Pulsatile Left Ventricular Assist Device: 5 (0F and 5M)<br>Nonpulsatile Left Ventricular Assist Device: 9 (1F and 8M)<br>Healthy: 10 (2F and 8M)	Pulsatile Left Ventricular Assist Device: 52 ± 14<br>Nonpulsatile Left Ventricular Assist Device: 48 ± 14<br>Healthy: 48 ± 14	MCA	Sit-to-Stand	0.05	TFA (PSD, coherence, nGain, phase) - band averages in VLF (0.02-0.07Hz), LF (0.07-0.20 Hz)<br>Autoregulatory Index
de Heus et al.	137 Total (61F and 76M)<br>Controls: 47 (20F and 27M)<br>Dementia: 53 (28F and 25M)<br>MCI: 37 (13F and 24M)	Controls: 69.4 (95% CI: 68.3-70.5)<br>Dementia: 73.1 (95% CI: 71.4-74.8)<br>MCI: 69.2 (95% CI: 66.4-72.0)	MCA	Sit-to-Stand	0.05	TFA (coherence, gain, nGain, phase) - narrow band averages in VLF (0.04-0.06Hz)<br>Autoregulatory Index
Diehl et al.	80 Total (41F and 39M)<br>Healthy: 50 (25F and 25M)<br>Occlusive Cerebrovascular Disease: 20 (11F and 9M)<br>Intracranial AV Malformation: 10 (5F and 5M)	All: 44.7 ± 15<br>Occlusive Cerebrovascular Disease: 48.7 ± 17.9<br>Intracranial AV Malformation: 34.3 ± 15.8	MCA	Deep Breathing	0.10	TFA (phase)
Ding et al.	44 Total (20F and 24M)<br>Controls: 22 (10F and 12M)<br>TBI: 22 (10F and 12M)	Controls: 44.2 ± 13.2<br>TBI: 43.4 ± 14.1	MCA	Sit-to-Stand	0.05	TFA (PSD, coherence, nGain, phase)
Drapeau et al.	17 Total (0F and 17M)<br>HIIT85: 8 (0F and 8M)<br>HIIT115: 9 (0F and 9M)	HIIT85: 26 ± 5<br>HIIT115: 28 ± 6	MCA	Squat-Stand manevuers	0.05, 0.10	TFA (PSD, coherence, gain, nGain, phase)
Elting et al.	16 Total (8F and 8M)	32.5 ± 9.5	MCA	Leg Raises	0.10	TFA (PSD, coherence, gain, phase)<br>Autoregulatory Index

Favre et al.	26 Total (13F and 13M)	Female: 25 (r: 22-31)<br>Male: 25 (r: 20-35)	MCA, ACA	Squat-Stand manevuers	0.05	TFA (PSD, coherence, phase, nGain)
Gommer et al.	19 Total (5F and 14M)	28 (r: 18-53)	MCA	Deep Breathing	0.10	TFA (coherence, phase, gain, nGain)<br>Autoregulatory Index
Gong et al.	47 Total (15F and 32M)<br>Controls: 22 (7F and 15M)<br>Moderate Stenosis: 17 (6F and 11M)<br>Severe Stenosis: 8 (2F and 6M)	Controls: 50 ± 9<br>Moderate Stenosis: 51 ± 11<br>Severe Stenosis: 54 ± 13	MCA, PCA	Deep Breathing	0.10	TFA (coherence, gain, phase) - band averages (0.06-0.12)
Hamner et al.	9 Total (4F and 5M)	27.1 ± 0.77 (r: 21-30)	MCA	Oscillatory LBNP	0.03, 0.04, 0.05, 0.06, 0.07, 0.08	TFA (PSD, coherence, gain)
Hamner et al.	13 Total (0F and 13M)	23.8 ± 0.5	MCA	Oscillatory LBNP	NR	TFA (PSD, coherence, gain, phase)<br>PPR (Falling Slope, Rising Slope, Autoregulatory Slope, Autoregulatory Range)
Hamner et al.	9 Total (3F and 6M)	r: 23-40	MCA	Oscillatory LBNP	0.03, 0.05, 0.07	TFA (coherence, phase, gain)
Hamner et al.	11 Total (4F and 7M)	r: 21-40	MCA	Oscillatory LBNP	0.03, 0.04, 0.05, 0.06, 0.07, 0.08	TFA (PSD, coherence, gain)
Hamner et al.	43 Total (17F and 26M)	r:21 - 40	MCA	Oscillatory LBNP	0.03	PPR (Falling Slope, Rising Slope, Autoregulatory Slope, Autoregulatory Range)
Hughson et al.	8 Total (2F and 6M)	25 ± 5.9	MCA	Head-Up Tilt	0.05	TFA (gain, phase) - band averages in VLF (0.00-0.07 Hz), LF (0.07-0.20 Hz), HF (0.20-0.30Hz)
Janzarik et al.	97 Total (97F and 0M)<br>Pregnant Without Subsequent Preeclampsia: 62 (62F and 0M)<br>Pregnant With Subsequent Preeclampsia: 9 (9F and 0M)<br>Non-Pregnant Control: 26 (26F and 0M)	Pregnant Without Subsequent Preeclampsia: 31.7 ± 5.3<br>Pregnant With Subsequent Preeclampsia: 31.4 ± 4.9<br>Non-Pregnant Control: NR	MCA, PCA	Deep Breathing	0.10	TFA (gain, phase)
Janzarik et al.	50 Total (50F and 0M)<br>Preeclampsia: 25 (25F and 0M)<br>Controls: 25 (25F and 0M)	Preeclampsia: 35.6 ± 5.7<br>Controls: 35.2 ± 4.8	MCA, PCA	Deep Breathing	0.10	TFA (nGain, phase)
Janzarik et al.	50 Total (50F and 0M)<br>Preeclampsia: 25 (25F and 0M)<br>Controls: 25 (25F and 0M)	Preeclampsia: 32.1 ± 4.5<br>Controls: 32.7 ± 4.9	MCA, PCA	Deep Breathing	0.10	TFA (nGain, phase)
Junejo et al.	83 Total (34F and 49M)<br>Healthy Controls: 24 (11F and 13M)<br>Atrial Fibrillaton: 30 (9F and 21M)<br>Hypertension: 29 (14F and 15M)	Healthy Controls: 68 [IQR: 66; 70]<br>Atrial Fibrillaton: 69 [IQR: 63; 72]<br>Hypertension:	MCA	Squat-Stand manevuers	0.10	TFA (coherence, gain, nGain, phase)

		68 [IQR: 65; 72]				
Katsogridakis et al.	10 Total (1F and 9M)	r: 27-63	MCA	Leg Cuff Oscillations	0.042, 0.083, 0.167	TFA (PSD, coherence) - band averages (0.05-0.15 Hz)
Klein et al.	40 Total (20F and 20M)<br>Young: 20 (9F and 11M)<br>Older: 20 (11F and 9M)	Young: 24 ± 4<br>Older: 71 ± 7	MCA	Sit-to-Stand	0.05	Delta MCAv, delta MAP, delta CVRi, delta CVCi
Kwan et al.	10 total (3F and 10M)	72.5 ± 11.4	MCA	Rhythmic handgrip	0.025	TFA (gain and phase)
Labrecque et al.	27 Total (0F and 27M)<br>Athletes: 19 (0F and 19M)<br>Controls: 8 (0F and 8M)	Athletes: 26 ± 5<br>Controls: 31 ± 4	MCA	Squat-Stand manevuers	0.05, 0.10	TFA (PSD, coherence, gain, nGain, phase)
Labrecque et al.	11 Total (0F and 11M)	25 ± 4	MCA, PCA	Squat-Stand manevuers	0.05, 0.10	TFA (PSD, coherence, gain, nGain, phase)
Labrecque et al.	22 Total (11F and 11M)	Female: 25 ± 4<br>Male: 24 ± 2	MCA	Squat-Stand manevuers	0.05, 0.10	TFA (PSD, coherence, gain, nGain, phase)
Labrecque et al.	18 Total (10F and 8M)	Total: 25 ± 5<br>Female: 24 ± 3<br>Male: 26 ± 6	MCA, PCA	Squat-Stand manevuers	0.05, 0.10	Hysteresis (absolute delta MCAv, absolute delta MAP, time delta MCAv, time delta MAP, delta MCAv time / delta MAP time, %MCAvT/%MAPT [%/%])
Labrecque et al.	74 Total (9F and 65M)	26 (r: 20-74)	MCAv	Squat-Stand manevuers	0.05, 0.10	Hyesteresis (absolute change / time in MCA, absolute change / time in MAP, delta MCAv time / delta MAP time)
Lagi et al.	20 Total (8F and 12M)<br>Cirrhosis and Portal Hypertension: 10 (4F and 6M)<br>Controls: 10 (4F and 6M)	Cirrhosis and Portal Hypertension: 48 ± 11 (r: 33-61)<br>Controls: 48 ± 9 (r: 36-60)	MCA	Neck Suction	0.10	TFA (phase)
Lewis et al.	22 Total (3F and 19M)	41 ± 18 (r: 17-78)	MCA	Ventilated Breathing	0.10	TFA (phase)<br>Correlation coefficient index Mx (mean)
Liu et al.	8 Total (2F and 6M)	N/A	MCA	Oscillatory LBNP	0.083	Linear Autoregressive with Exogenous Input (ARX) Model
Lucas et al.	16 Total (6F and 10M)	25 ± 4	MCA	Deep Breathing	0.10	TFA (PSD, coherence, gain, phase)
Malenfant et al.	22 Total (16F and 6M)<br>Pulmonary Arterial Hypertension: 11 (8F and 3M)<br>Control: 11 (8F and 3M)	Pulmonary Arterial Hypertension: 44 ± 12<br>Control: 43 ± 15	MCA	Squat-Stand manevuers	0.05, 0.10	TFA (PSD, coherence, nGain, phase)
Marthol et al.	20 Total (NR)<br>Type 2 Diabetic: 9 (NR)<br>Controls: 11 (NR)	Type 2 Diabetic: 54.5 ± 10.0<br>Controls: 56.2 ± 5.4	MCA	Neck Suction	0.1	TFA (PSD, gain, phase)
Maxwell et al.	19 Total (6F and 13M)<br>Remote Ischaemic Preconditioning + Exercise: 10 (4F and 6M)<br>Remote Ischaemic Preconditioning: 9 (2F and 7M)	Remote Ischaemic Preconditioning + Exercise: 52 ± 8<br>Remote Ischaemic	MCA	Squat-Stand manevuers	0.05, 0.10	TFA (PSD, coherence, gain, nGain, phase)

		Preconditioning: 51 ± 12				
Maxwell et al.	206 Total (123F and 83M)<br>Healthy: 166 (109F and 57M)<br>CVD Risk: 40 (14F and 26M)<br>Young: 93 (48F and 45M)<br>Middle: 93 (44F and 18M)<br>Older: 93 (31F and 20M)	Healthy: 37 ± 14<br>CVD Risk: 56 ± 3<br>Young: 26 ± 5 (r: 18-35)<br>Middle: 47 ± 6 (r: 36-55)<br>Older: 61 ± 4 (r: 56-70)	MCA	Squat-Stand manevuers	0.10	TFA (PSD, coherence, gain, nGain, phase)
Mense et al.	28 Total (8F and 20M)<br>Carotid stenosis: 18 (3F and 15M)<br>Control: 10 5F and 5M	Median (IQR)<br>Carotid Stenosis: 69 (65; 71)<br>Control: 65 (63; 68)	MCA	Deep Breathing	0.10	Tieck's ARI during Valsalva phase IV<br>Trigonometric regressive spectral analysis (phase)
Mol et al.	94 Total (44F and 50M)<br>MCI: 37 (12F and 25M)<br>Alzheimer's Disease: 57 (32F and 25M)	MCI: 69.2 ± 8.4<br>Alzheimer's Disease: 73.3 ± 6.1	MCA	Sit-to-Stand	0.05	TFA (coherence, gain, phase) - band averages in VLF (0.02-0.07 Hz), LF (0.07-0.20 Hz), HF (0.20-0.50 Hz)
Newel et al.	25 Total (13F and 12M)	Female: 24.5 ± 3.6<br>Male: 26.9 ± 4.6	MCA, PCA	Squat-Stand manevuers	0.05, 0.10	TFA (PSD, coherence, gain, nGain, phase)
Oudegeest-Sander et al.	58 Total (21F and 37M)<br>Young: 20 (11F and 9M)<br>Elderly: 20 (7F and 13M)<br>Older Elderly: 18 (3F and 15M)	Young: 21 ± 2 (r: 21-28)<br>Elderly: 66 ± 1 (r: 65-69)<br>Older Elderly: 78 ± 3 (r: 74-86)	MCA	Sit-to-Stand	0.05	TFA (PSD, coherence, gain, nGain, phase) - band-averages in VLF (0.02-0.07)
Panerai et al.	32 Total (16F and 16M)	40.3 ± 17.6 yr (range 20-71)	MCA	Squat-Stand manevuers	0.05	TFA (PSD, coherence)
Panerai et al.	10 Total (2F and 8M)	22 ± 1	MCA	Squat-Stand manevuers	0.05	Hysteresis<br>Autoregulatory Index
Panerai et al.	14 Total (7F and 7M)	32 ± 9 (r: 23-51)	MCA	Deep Breathing	0.10, 0.167, 0.25	Autoregressive-moving average (ARMA) modelling<br>Autoregulatory Index
Perry et al.	36 Total (3F and 33M)<br>Resistance Trained: 12 (0F and 12M)<br>Endurance Trained: 12 (0F and 12M)<br>Healthy Sedentary: 12 (3F and 9M)	Resistance Trained: 25 ± 6<br>Endurance Trained: 28 ± 9<br>Healthy Sedentary: 26 ± 6	MCA	Squat-Stand manevuers	0.05, 0.10	TFA (PSD, coherence, gain, nGain, phase)
Potter et al.	14 Total (7F and 7M)	32 ± 9	MCA	Deep Breathing	0.10, 0.167	TFA (coherence, nGain, phase)
Purkayastha et al.	7 Total (2F and 5M)	26 ± 1	MCA	Neck Suction	0.10	TFA (PSD, coherence, gain, phase)
Querido et al.	16 Total (7F and 9M)	29 ± 5	MCA	Squat-Stand manevuers	0.10	MCAvmean as a function of MAP with the slope of the linear regression representing dCA gain
Reed et al.	12 Total (4F and 8M)	23 ± 2	MCA, PCA	Squat-Stand manevuers	0.05, 0.10	TFA (coherence, gain, nGain) - band averages in VLF (0.02-0.07 Hz), LF (0.07-0.20 Hz).
Reinhard et al.	60 Total (6F and 54M)<br>Bilateral ICA Stenosis/Occlusion:	Bilateral ICA Stenosis/Occlusi	MCA	Deep Breathing	0.10	TFA (phase)

	30 (4F and 26M)<br>Unilateral Severe Stenosis or Occlusion and No Contralateral Stenosis: 30 (2F and 28M)	on: 68 ± 7<br>Unilateral Severe Stenosis or Occlusion and No Contralateral Stenosis: 66 ± 8				
Reinhard et al.	69 Total (51F and 18M)<br>Migraine Without Aura: 17 (13F and 4M)<br>Migraine With Aura: 17 (12F and 5M)<br>Controls: 35 (26F and 9M)	Total Migraine: 28.7 ± 7.2<br>Migraine Without Aura: 29.1 ± 7.6<br>Migraine With Aura: 28.4 ± 7.1<br>Controls: 28.5 ± 6.4	MCA, PCA, PICA	Deep Breathing	0.10	TFA (gain, phase)<br>Correlation coefficient index Dx
Reinhard et al.	56 Total (28F and 28M)	29 ± 10	MCA, PICA	Deep Breathing	0.10	TFA (gain, phase)<br>Correlation coefficient index Mx (mean) and Dx (diastolic)
Reinhard et al.	165 Total (24F and 141M)	66 ± 8	MCA	Deep Breathing	0.10	TFA (phase)
Reinhard et al.	Total 111 (NR)	NR	MCA	Deep Breathing	0.10	TFA (phase)
Reinhard et al.	36 Total (6F and 30M)<br>Severe ICA stenosis: 19 (1F and 18M)<br>Controls: 17 (5F and 12M)	Severe ICA stenosis: 61 ± 8<br>Controls: 58 ± 4	MCA	Deep Breathing	0.10	TFA (coherence, phase)
Reinhard et al.	29 Total (10F and 19M)<br>Cerebral Amyloid Angiopathy: 15 (5F and 10M)<br>Controls: 14 (5F and 9M)	Cerebral Amyloid Angiopathy: 69.6 ± 10.5<br>Controls: 68.3 ± 9.9	MCA, PCA	Deep Breathing	0.10	TFA (coherence, nGain, phase)
Reinhard et al.	68 Total (NR)<br>Phase: 47 (5F and 42M)<br>Dx: 55 (8F and 47M)	Phase: 65 ± 9<br>Dx: 65 ± 8	MCA	Deep Breathing	0.10	TFA (phase)<br>Correlation coefficient index Dx (diastole)
Reinhard et al.	129 Total (13F and 116M)	65 ± 9	MCA	Deep Breathing	0.10	TFA (PSD, gain, phase)
Rickards et al.	8 Total (5F and 3M)	28 ± 1	MCA	Oscillatory LBNP	0.05, 0.10	TFA (PSD, coherence) - band averages in VLF (0.04-0.07 Hz), LF (0.07-0.20 Hz)
Rodrigues et al.	14 Total (14F and 0M)<br>Sham: 6 (6F and 0M)<br>IMT: 8 (8F and 0M)	Sham: 66 ± 3<br>IMT: 64 ± 3	MCA	Deep Breathing	0.1	TFA (coherence, gain, phase)
Roy et al.	36 Total (4F and 32M)<br>Sedentary: 12 (4F and 8M)<br>Endurance: 12 (0F and 12M)<br>Resistance: 12 (0F and 12M)	Sedentary: 29 (r: 18-33)<br>Endurance: 26 (r: 18-48)<br>Resistance: 24 (r: 18-41)	MCA	Squat-Stand manevuers	0.05, 0.10	Hysteresis (absolute delta MCAv, absolute delta MAP, time delta MCAv, time delta MAP, delta MCAv time / delta MAP time, %MCAvT/%MAPT [%/%])
Saleem et al.	18 Total (9F and 9M)	23 ± 0.67	MCA	Oscillatory LBNP	0.03, 0.05, 0.07	TFA (PSD, coherence, gain, phase), PPR, LOWESS
Ševerdija et al.	37 Total (0F and 37M)	61 ± 6	MCA	Cyclical Pump Flow	0.10	TFA (PSD, coherence, gain, phase)
Smail et al.	22 (8F and 14M)	22 ± 2	MCA	Squat-Stand Maneuvers	0.05, 0.10	TFA (PSD, coherence, gain, nGain, phase)
Smirl et al.	32 Total (3F and 29M)<br>Lowlanders: 16 (3F and 13M)<br>Sherpa: 16 (0F and 16M)	Lowlanders: 28.4 ± 7.2<br>Sherpa: 32.5 ± 14.5	MCA, PCA	Squat-Stand manevuers	0.05, 0.10	TFA (PSD, coherence, gain, phase)

Smirl et al.	20 Total (4F and 16M)	26.7 ± 6.6	MCA	Squat-Stand manevuers	0.05, 0.10	TFA (PSD, coherence, gain, nGain, phase, impulse step response)
Smirl et al.	16 Total (4F and 12M)	27.2 ± 7.2	MCA, PCA	Squat-Stand manevuers	0.05, 0.10	TFA (PSD, coherence, gain, nGain, phase)
Smirl et al.	19 Total (2F and 17M)<br>Younger: 10 (0F and 10M)<br>Older: 9 (2F and 7M)	Younger: 24.8 ± 2.7<br>Older: 66.4 ± 3.7	MCA	Squat-Stand manevuers, Oscillatory LBNP	0.05, 0.10	TFA (PSD, coherence, gain, nGain, phase)
Smirl et al.	17 Total (0F and 17F)<br>Young: 9 (0F and 9M)<br>Older: 8 (0F and 8M)	Young: 24.8 ± 2.7<br>Older: 66.4 ± 3.7	MCA	Oscillatory LBNP	0.05, 0.10	TFA (PSD, coherence, gain, nGain, phase)
Smirl et al.	27 Total (0F and 27M)<br>Heart Transplant Receipents: 8 (0F and 8M)<br>Age-Matched Controls: 9 (0F and 9M)<br>Donor-Matched Controls: 10 (0F and 10M)	Heart Transplant Receipents: 62 ± 8<br>Age-Matched Controls: 63 ± 8<br>Donor-Matched Controls: 27 ± 5	MCA	Squat-Stand manevuers	0.05, 0.10	TFA (PSD, coherence, gain, phase)
Smirl et al.	7 Total (7M and 0F)	24.1 ± 1.5	MCA	Squat-Stand manevuers	0.05, 0.10	TFA (PSD, coherence, nGain, phase)
Sommerlade et al.	93 Total (11F and 82M)	67 ± 8	MCA	Deep Breathing	0.10	TFA (coherence, phase)
Sprick et al.	35 Total (13F and 22M)<br>Chronic Kidney Disease: 15 (5F and 10M)<br>Control: 20 (8F and 12M)	Chronic Kideny Disease: 64 ± 10<br>Control: 59 ± 9	MCA	Sit-to-Stand	0.05, 0.10	TFA (PSD, coherence, gain, nGain, phase)
Stefanidis et al.	58 Total (29F and 29M)<br>Young: 29 (17F and 12M)<br>Older: 29 (12F and 17M)	Young: 23 ± 4 (r: 18-30)<br>Older: 68 ± 3 (r: 60-75)	MCA	Sit-to-Stand	0.05	Delta MCAv / delta MAP (%/%)
Stefanidis et al.	51 Total (26F and 25M)<br>Younger: 27 (17F and 10M)<br>Olderer: 24 (9F and 15M)	Younger: 23 ± 4 (r: 18-30)<br>Older: 68 ± 4 (r: 60-75)	MCA	Sit-to-Stand	0.10	Delta MCAv / delta MAP (%/%)
Stok et al.	22 Total (4F and 18M)	40 ± 8	MCA	Deep Breathing, Head-Up Tilt	0.10, 0.167, 0.25	TFA (phase, nGain)
Tan et al.	43 Total (17F and 26M)	r: 21-40	MCA	Oscillatory LBNP	0.03, 0.04, 0.05, 0.06, 0.07, 0.08	PPR (Falling Slope, Rising Slope, Autoregulatory Slope, Autoregulatory Range)
Tan et al.	16 Total (7F and 9M)	r: 21-30	MCA	Oscillatory LBNP	0.03, 0.04, 0.05, 0.06, 0.07, 0.08	TFA (coherence, gain)<br>PPR (Falling Slope, Rising Slope, Autoregulatory Slope, Autoregulatory Range)
Tarumi et al.	40 aMCI total (23F and 17M)<br>Dippers (BP drop during sleep ≥10%): 26 (15F and 11M)<br>Non-dippers (BP drop during sleep < 10%): 14 (8F and 6M)	Dippers: 63 ± 4<br>Non-dippers: 68 ± 8	MCA	Sit-to-Stand	0.05	TFA (coherence, gain, nGain, phase)
Tarumi et al.	42 Total (22F and 20M)<br>Controls: 15 (6F and 9M)<br>Amnestic MCI: 27 (16F and 11M)	Controls: 67 ± 8<br>Amnestic MCI: 65 ± 6	MCA	Sit-to-Stand	0.05	TFA (coherence, nGain, phase) - band averages in VLF (0.02-0.07 Hz), LF (0.07-0.20 Hz), HF (0.20-0.30 Hz)
Tomoto et al.	60 Total (33F and 27M)<br>Young Sedentary: 20 (11F and 9M)	Young Sedentary: 32 ± 7	MCA	Sit-to-Stand	0.05	TFA (PSD, coherence, nGain, phase)

	Middle Sedentary: 20 (11F and 9M)<br>Middle Athlete: 20 (11F and 9M)	Middle Sedentary: 53 ± 5<br><br>Middle Athlete: 53 ± 4				
Tutaj et al.	32 Total (NR)<br>Controls: 11 (NR)<br>Normal Pressure Glaucoma: 10 (NR)<br>Primary Open Angle Glaucoma: 11 (NR)	Controls: 51 ± 19<br>Normal Pressure Glaucoma: 57 ± 18<br>Primary Open Angle Glaucoma: 52 ± 11	MCA	Deep Breathing	0.1	TFA (PSD, coherence, gain)
Tzeng et al.	179 Total (53F and 126M)<br>Dataset A: 105 (34F and 71M)<br>Dataset B: 29 (8F and 21M)<br>Dataset C: 29 (6F and 23M)<br>Dataset D: 16 (5F and 11M)	A: 26 ± 7<br><br>B: 30 ± 10<br><br>C: 32 ± 12<br><br>D: 22 ± 3.2	MCA	Squat-Stand manevuers	0.05, 0.10	TFA (coherence, gain, nGain, phase)
Tzeng et al.	8 Total (1F and 7M)	25 ± 5	MCA	Oscillatory LBNP	0.05, 0.10	TFA (PSD, coherence, gain, phase)
van Beek et al.	27 Total (8F and 19M)	76 ± 4 (r: 71-86)	MCA	Sit-to-Stand	0.05, 0.10	TFA (PSD, coherence, gain, phase)
van Beek et al.	41 Total (18F and 23M)<br>Healthy: 20 (6F and 14M)<br>Alzheimer's: 21 (12F and 9M)	Healthy: 74.5 ± 2.8<br>Alzheimer's: 72.3 ± 5.7	MCA	Sit-to-Stand	0.05, 0.10	TFA (PSD, coherence, gain, nGain, phase)
Wallis et al.	18 (6F and 12M)	21 ± 1	MCA	Squat-Stand Maneuvers	0.05, 0.10	TFA (PSD, coherence, gain, nGain, phase)
Worley et al.	13 (5F and 8M)	26 ± 4	MCA	Oscillatory LBNP	0.02	TFA (coherence, gain, phase)
Wright et al.	14 Total (0F and 14M)	19.0 ± 1.4	MCA	Squat-Stand manevuers	0.05, 0.10	TFA (coherence, nGain, phase)
Wright et al.	81 Total (0F and 81M)<br>Baseline No Concussion History: 42 (0F and 42M)<br>Baseline Concussion History: 31 (0F and 31M)<br>Concussion: 18 (0F and 18M)	Baseline No Concussion History: 19.0 (1.4)<br>Baseline Concussion History: 19.6 (1.9)<br>Concussion: 18.6 ± 1.5	MCA	Squat-Stand manevuers	0.05, 0.10	TFA (PSD, coherence, gain, nGain, phase)
Wright et al.	52 (0F and 52M)	19.7 ± 1.6	MCA	Squat-Stand manevuers	0.05, 0.10	TFA (PSD, coherence, nGain, phase)
Xing et al.	136 total (81F and 55M)<br>Younger: 41 (25F and 16M)<br>Middle Age: 50 (30F and 20M)<br>Older: 45 (26F and 19M)	Younger: 32 ± 7 (F) and 34 ± 7 (M)<br>Middle Age: 58 ± 5 (F) and 53 ± 5 (M)<br>Older: 71 ± 4 (F) and 70 ± 4 (M)	MCA	Sit-to-Stand	0.05	TFA (PSD, coherence, nGain, phase)

Yoshida et al.	14 Total (0F and 14M)	24.1 ± 0.5	MCA	Oscillatory LBNP	0.01, 0.06	Correlations between physiological variables and CBv
Zhang et al.	12 Total (3F and 9M)	29 ± 6	MCA	Oscillatory LBNP	0.05	TFA (PSD, coherence, gain, phase)

Supplemental D: Studies Using Driven Techniques in Clinical Populations.

Driven Method	Author	Clinical Population	Vessel	Frequency	Outcomes
Cyclical Pump Flow	Ševerdija et al. 2014	Cardiopulmonary Bypass	MCA	0.10	TFA (PSD, coherence, gain, phase)
Deep Breathing	Aaron et al. 2021	Concussion	MCA	NR	PPR (Falling Slope, Rising Slope, Autoregulatory Slope, Autoregulatory Range)
Deep Breathing	Brown et al. 2008	Type II Diabetes	MCA	0.10	TFA (PSD, gain, phase)
Deep Breathing	Diehl et al. 1995	Artery Stenosis or Occlusion	MCA	0.10	TFA (phase)
Deep Breathing	Gong et al. 2013	Artery Stenosis or Occlusion	MCA, PCA	0.10	TFA (coherence, gain, phase) - band averages (0.06-0.12)
Deep Breathing	Janzarik et al. 2013	Pregnant or Preeclampsia	MCA, PCA	0.10	TFA (gain, phase)
Deep Breathing	Janzarik et al. 2018	Pregnant or Preeclampsia	MCA, PCA	0.10	TFA (nGain, phase)
Deep Breathing	Janzarik et al. 2019	Pregnant or Preeclampsia	MCA, PCA	0.10	TFA (nGain, phase)
Deep Breathing	Mense et al. 2010	Artery Stenosis or Occlusion	MCA	0.10	Tieck's ARI during Valsalva phase IV<br>Trigonometric regressive spectral analysis (phase)
Deep Breathing	Reinhard et al. 2001	Artery Stenosis or Occlusion	MCA	0.10	TFA (coherence, phase)
Deep Breathing	Reinhard et al. 2002	Artery Stenosis or Occlusion	MCA	0.10	TFA (phase)
Deep Breathing	Reinhard et al. 2003	Artery Stenosis or Occlusion	MCA	0.10	TFA (phase)
Deep Breathing	Reinhard et al. 2003	Artery Stenosis or Occlusion	MCA	0.10	TFA (PSD, gain, phase)
Deep Breathing	Reinhard et al. 2008	Artery Stenosis or Occlusion	MCA	0.10	TFA (phase)

Deep Breathing	Reinhard et al. 2011	Artery Stenosis or Occlusion	MCA	0.10	TFA (phase)<br>Correlation coefficient index Dx (diastole)
Deep Breathing	Reinhard et al. 2012	Migraineurs	MCA, PCA, PICA	0.10	TFA (gain, phase)<br>Correlation coefficient index Dx
Deep Breathing	Reinhard et al. 2019	Cerebral Amyloid Angiopathy	MCA, PCA	0.10	TFA (coherence, nGain, phase)
Deep Breathing	Sommerlade et al. 2012	Artery Stenosis or Occlusion	MCA	0.10	TFA (coherence, phase)
Deep Breathing	Tutaj et al. 2004	Glaucoma	MCA	0.1	TFA (PSD, coherence, gain)
Leg Cuff Oscillations	Aaslid et al. 2007	Traumatic Brain Injury	MCA	0.03	Autoregulatory Gain Up = delta CCP Up / ABP Up<br>Autoregulatory Gain Down = delta CCP Down / delta ABP Down
Neck Suction	Lagi et al. 2002	Cirrhosis and Portal Hypertension	MCA	0.10	TFA (phase)
Neck Suction	Marthol et al. 2006	Type II Diabetes	MCA	0.1	TFA (PSD, gain, phase)
Rhythmic handgrip	Kwan et al. 2004	Stroke	MCA	0.025	TFA (gain and phase)
Sit-to-Stand	Cornwell et al. 2014	Left Ventricular Assist Device	MCA	0.05	TFA (PSD, coherence, nGain, phase) - band averages in VLF (0.02-0.07Hz), LF (0.07-0.20 Hz)<br>Autoregulatory Index
Sit-to-Stand	de Heus et al. 2018	Alzheimer's, Dementia, and/or MCI	MCA	0.05	TFA (coherence, gain, nGain, phase) - narrow band averages in VLF (0.04-0.06Hz)<br>Autoregulatory Index
Sit-to-Stand	Ding et al. 2020	Traumatic Brain Injury	MCA	0.05	TFA (PSD, coherence, nGain, phase)
Sit-to-Stand	Mol et al. 2021	Alzheimer's, Dementia, and/or MCI	MCA	0.05	TFA (coherence, gain, phase) - band averages in VLF (0.02-0.07 Hz), LF (0.07-0.20 Hz), HF (0.20-0.50 Hz)
Sit-to-Stand	Sprick et al. 2022	Chronic Kidney Disease	MCA	0.05, 0.10	TFA (PSD, coherence, phase, gain, nGain)
Sit-to-Stand	Tarumi et al. 2014	Alzheimer's, Dementia, and/or MCI	MCA	0.05	TFA (coherence, nGain, phase) - band averages in VLF (0.02-0.07 Hz), LF (0.07-0.20 Hz), HF (0.20-0.30 Hz)

Sit-to-Stand	Tarumi et al. 2015	Alzheimer's, Dementia, and/or MCI	MCA	0.05	TFA (coherence, gain, nGain, phase)
Sit-to-Stand	van Beek et al. 2012	Alzheimer's, Dementia, and/or MCI	MCA	0.05, 0.10	TFA (PSD, coherence, gain, nGain, phase)
Squat-Stand Maneuvers	Burma et al. 2021	Heart Transplant Recipients	MCA	0.05, 0.10	TFA (PSD, coherence)
Squat-Stand Maneuvers	Claassen et al. 2009	Alzheimer's, Dementia, and/or MCI	MCA	0.025, 0.05, 0.10	TFA (PSD, coherence, gain, phase)
Squat-Stand Maneuvers	Junejo et al. 2020	Arterial Fibrillation and Hypertension	MCA	0.10	TFA (coherence, gain, nGain, phase)
Squat-Stand Maneuvers	Malenfant et al. 2017	Pulmonary Arterial Hypertension	MCA	0.05, 0.10	TFA (PSD, coherence, nGain, phase)
Squat-Stand Maneuvers	Maxwell et al. 2022	CVD Risk	MCA	0.10	TFA (PSD, coherence, gain, nGain, phase)
Squat-Stand Maneuvers	Smirl et al. 2014	Heart Transplant Recipients	MCA	0.05, 0.10	TFA (PSD, coherence, gain, phase)
Squat-Stand Maneuvers	Wright et al. 2018	Concussion	MCA	0.05, 0.10	TFA (coherence, nGain, phase)
Ventilated Breathing	Lewis et al. 2008	Traumatic Brain Injury	MCA	0.10	TFA (phase)<br>Correlation coefficient index Mx (mean)

Supplemental E: 0.05 Hz TFA Wilcoxon *r* Effect Sizes

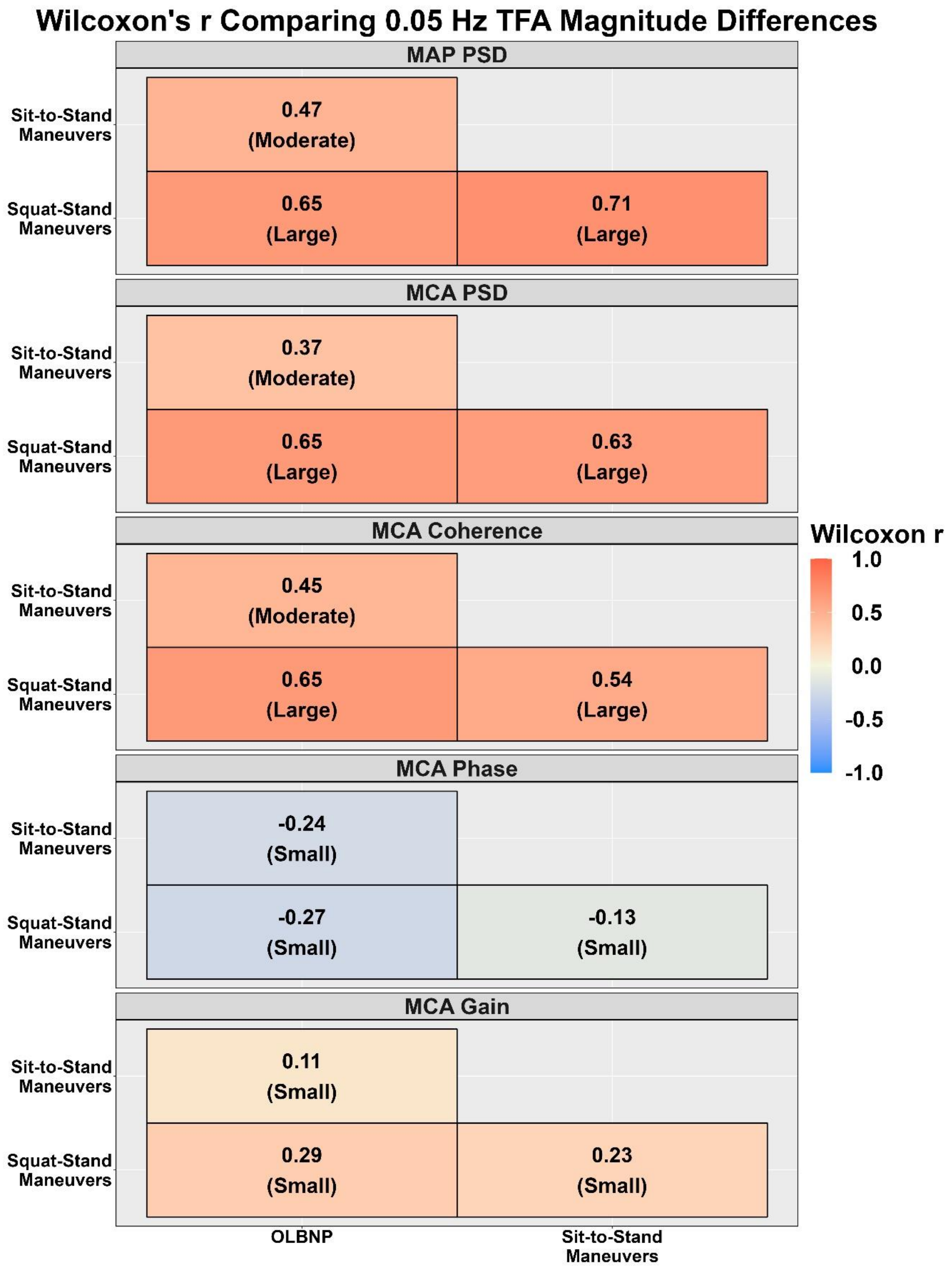

Supplemental F: 0.10 Hz TFA Wilcoxon *r* Effect Sizes

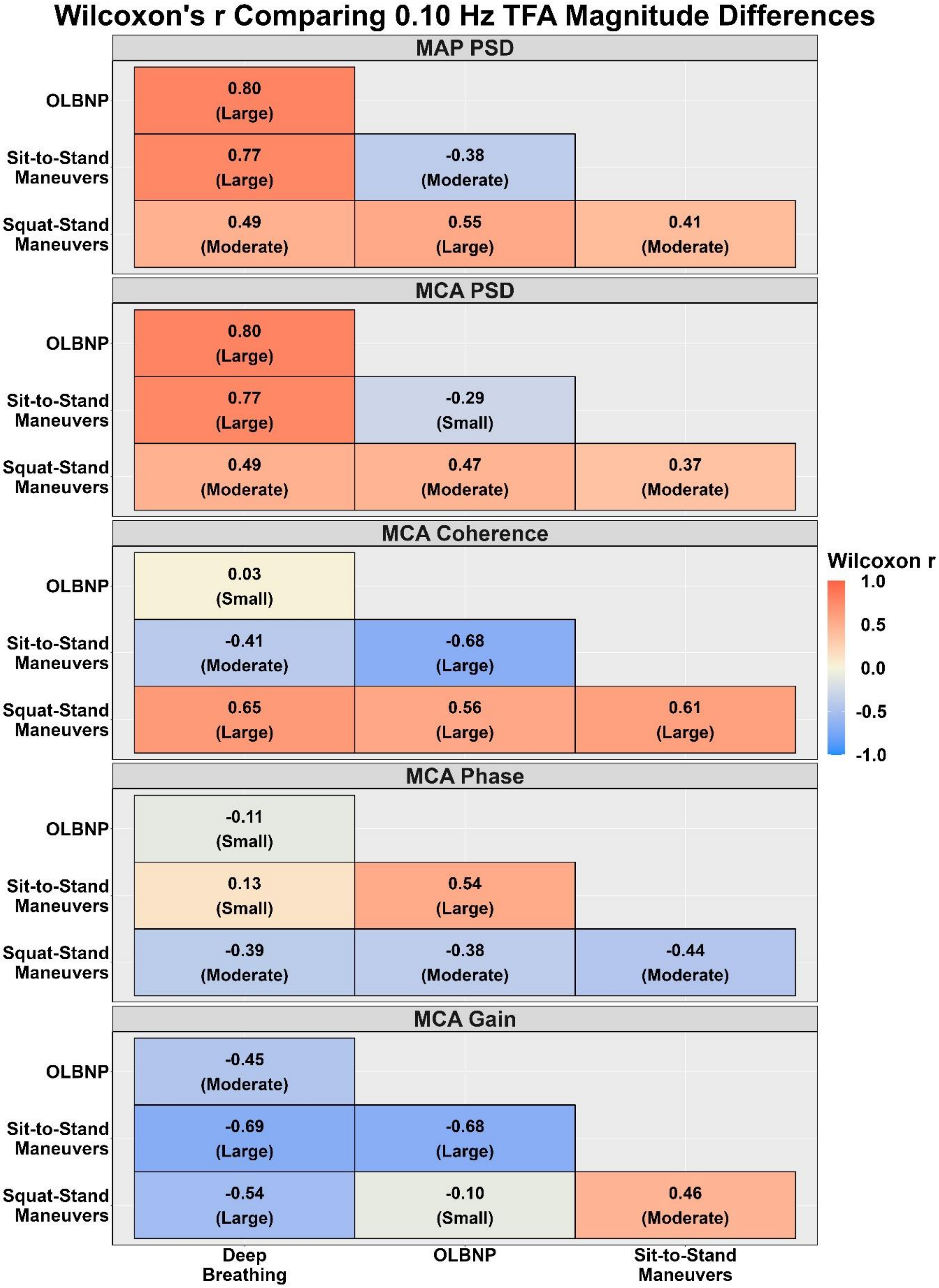

Supplemental G: Forest Plot of Middle Cerebral Artery 0.05 Hz Phase

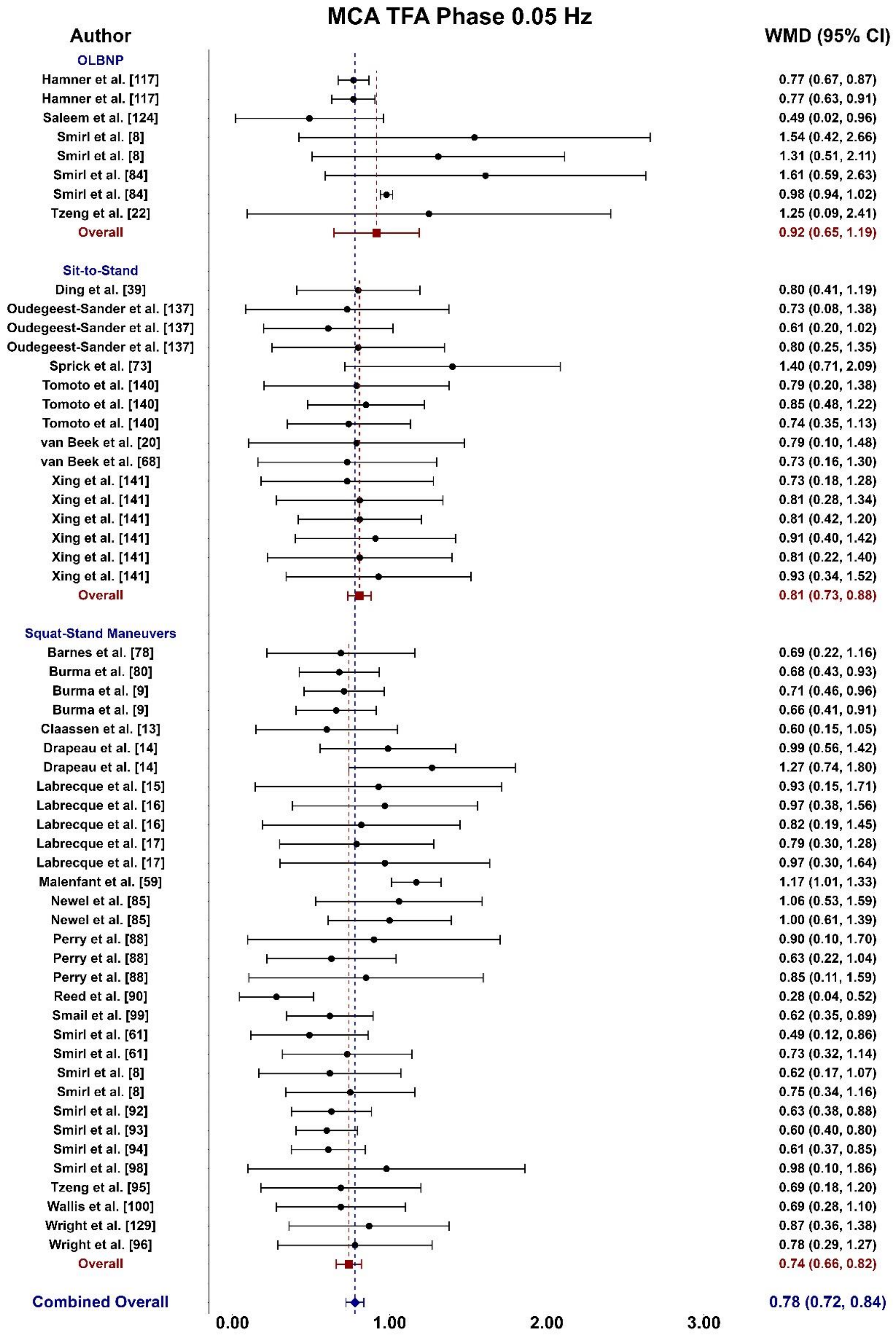

Supplemental H: Forest Plot of Middle Cerebral Artery 0.05 Hz Gain

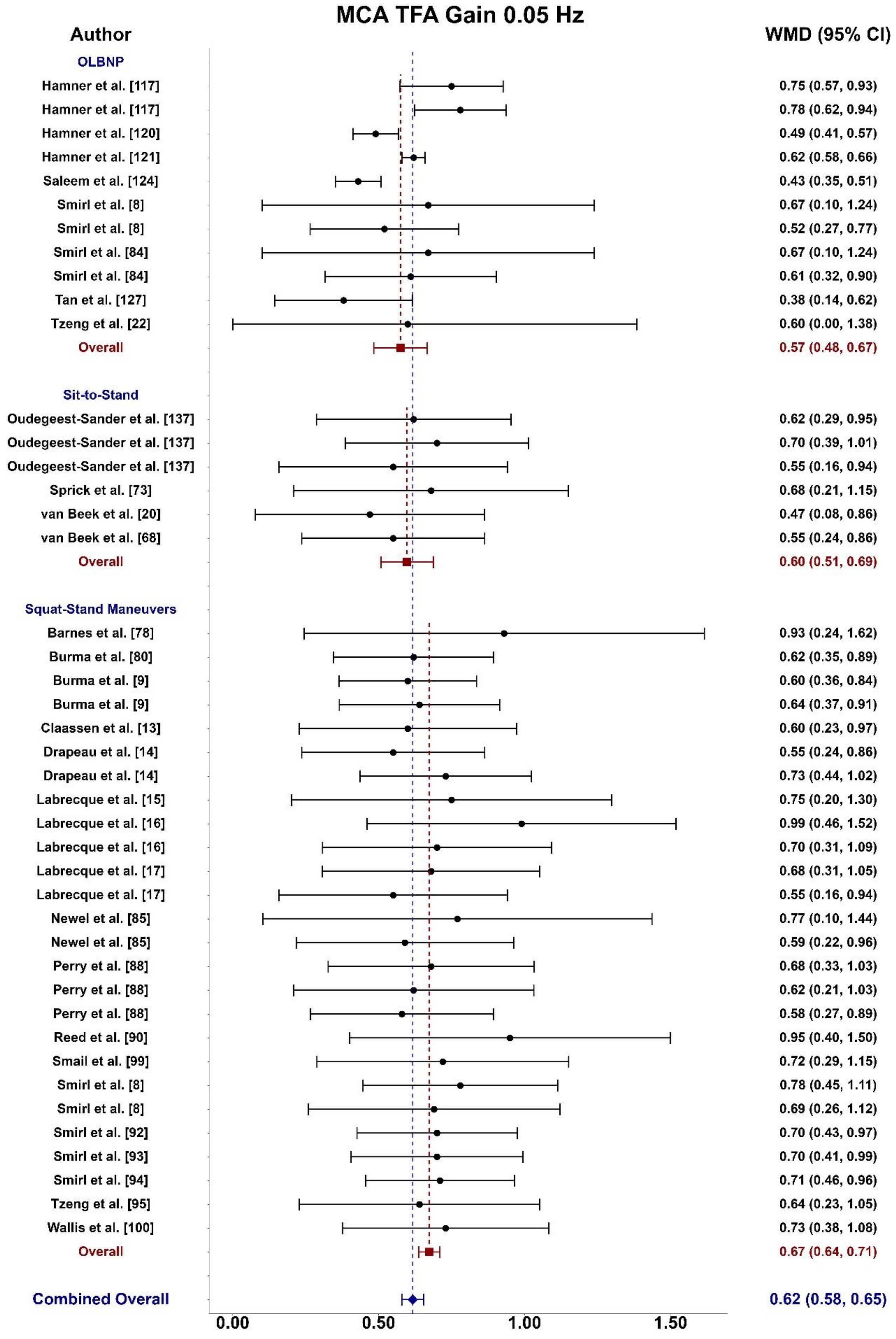

Supplemental I: Forest Plot of Middle Cerebral Artery 0.05 Hz Normalized Gain

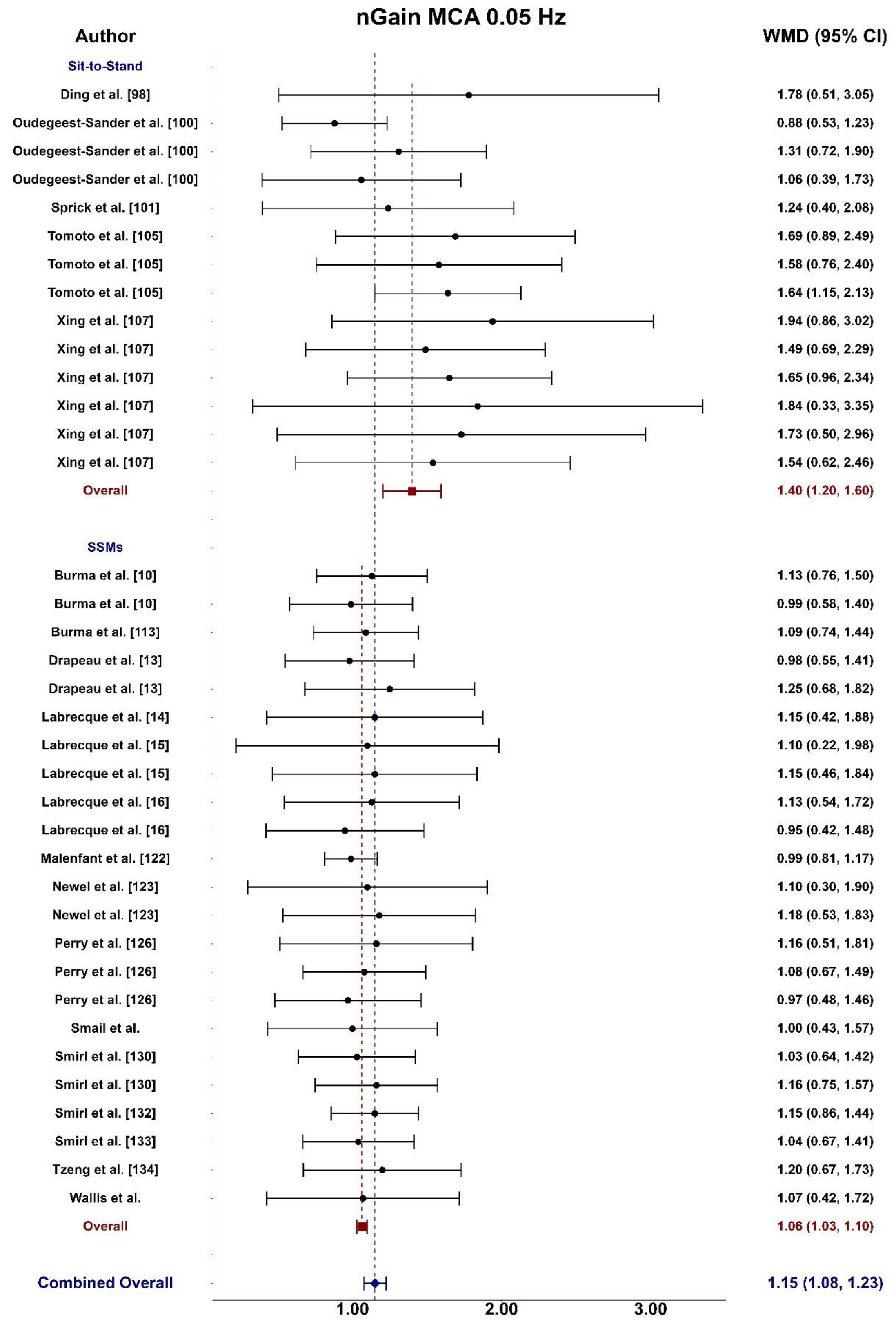

Supplemental J: Forest Plot of Middle Cerebral Artery 0.10 Hz Phase

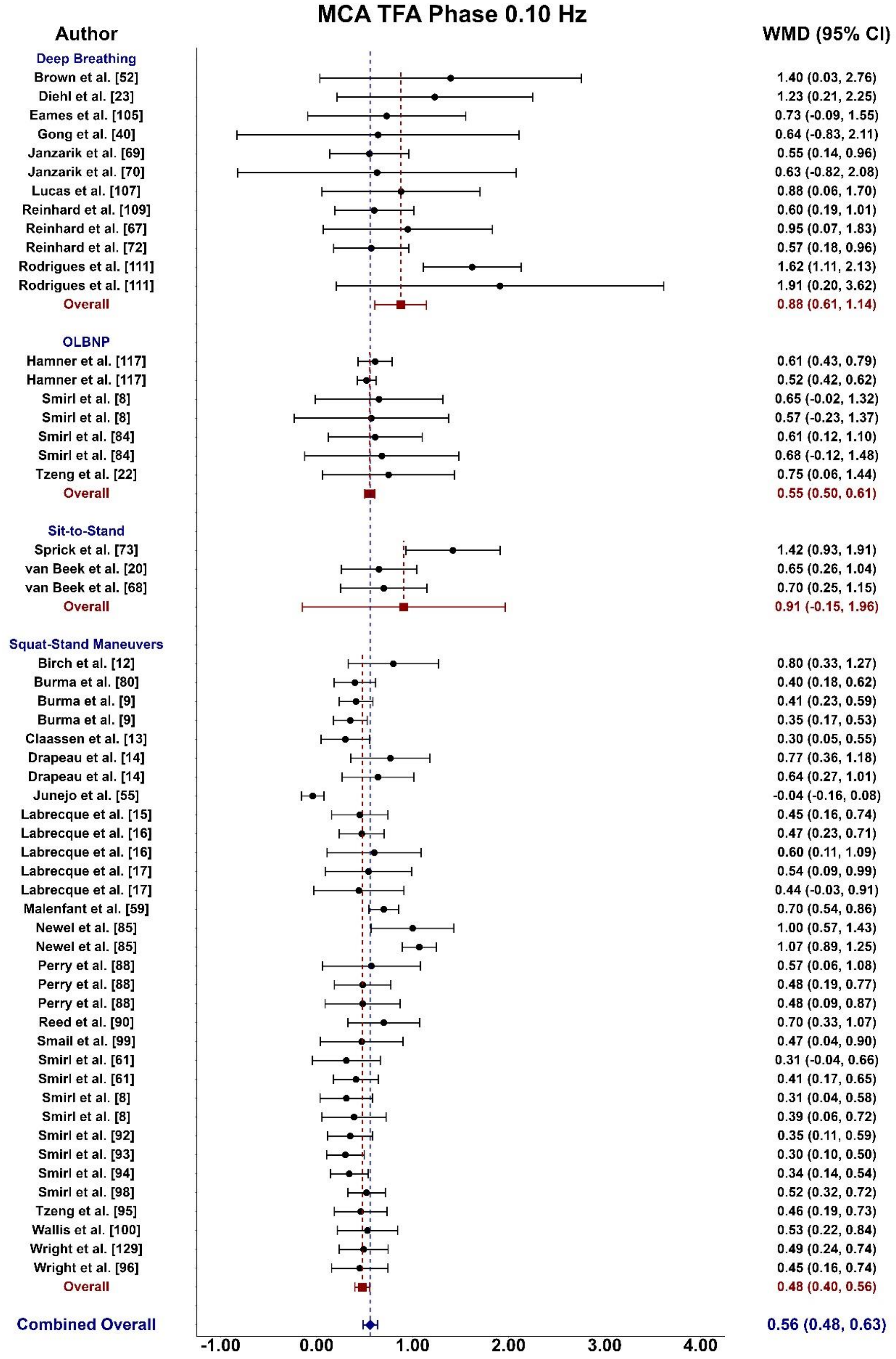

Supplemental K: Forest Plot of Middle Cerebral Artery 0.10 Hz Gain

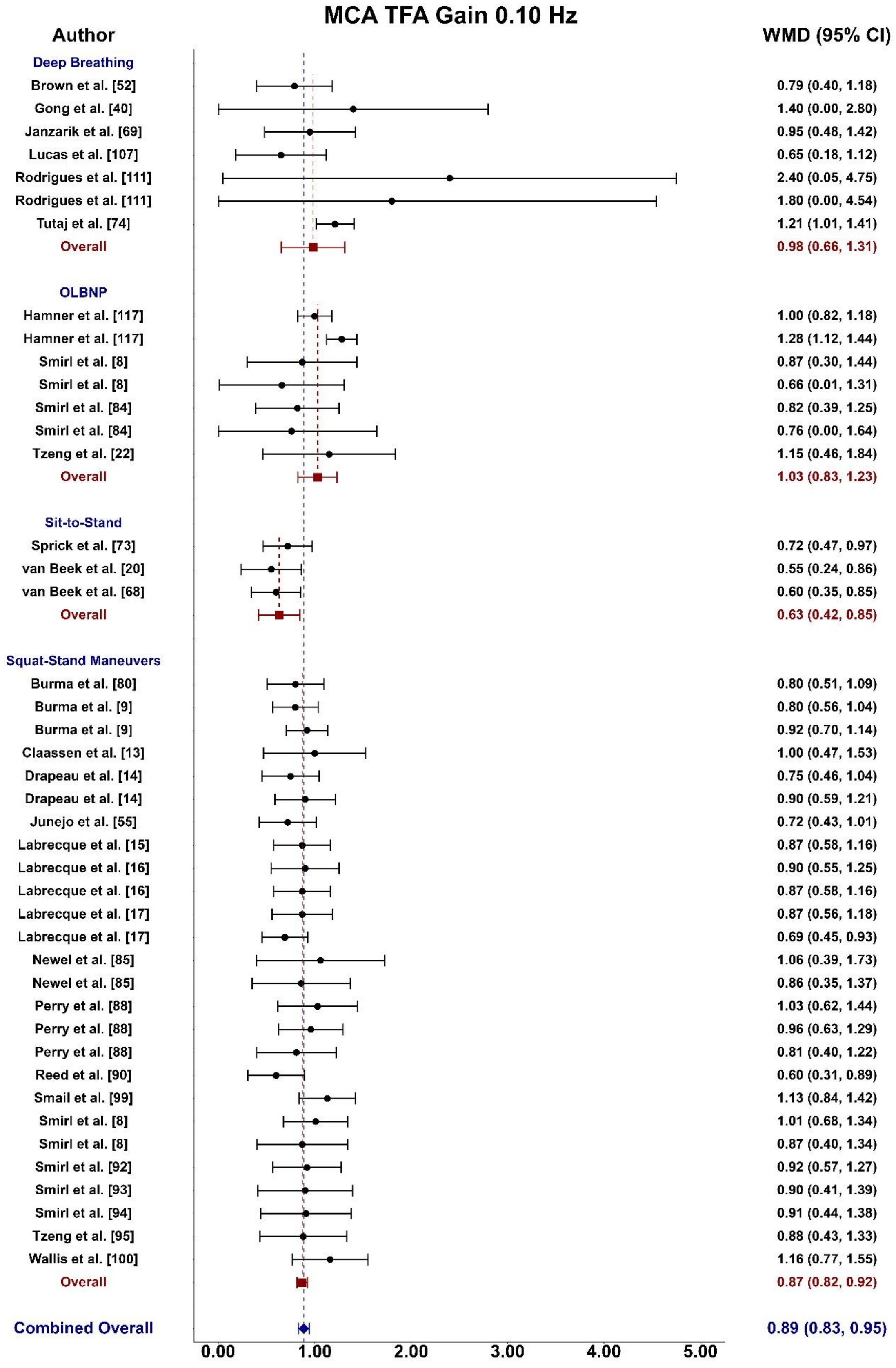

Supplemental L: Forest Plot of Middle Cerebral Artery 0.10 Hz Normalized Gain

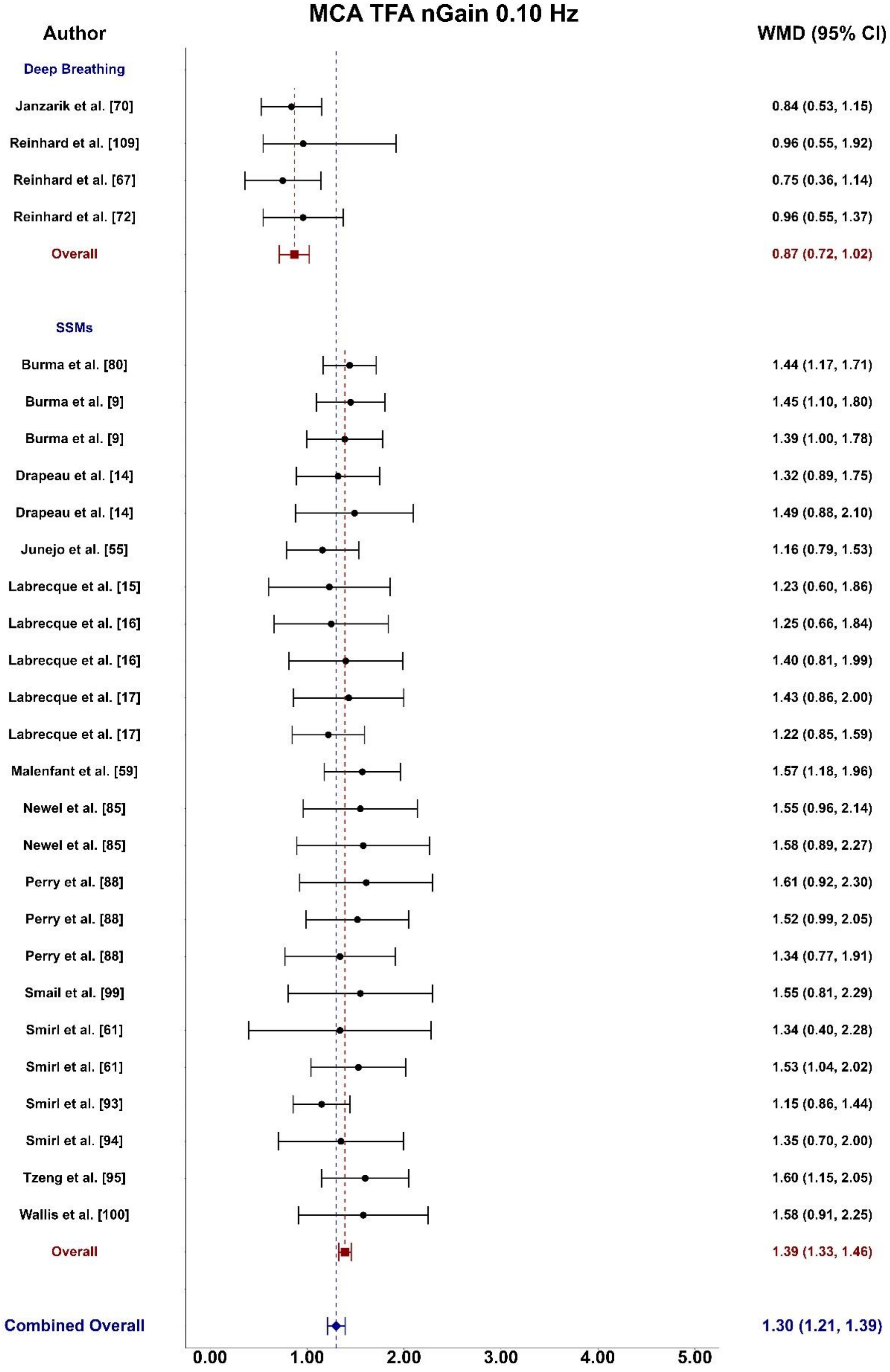

Supplemental M: Meta-Regression Beta Coefficients and 95% Confidence Intervals for Squat-Stand Maneuver TFA Estimates

Frequency	Vessel	Coefficient	Beta Coefficient (95% CI)	P-Value
0.05 Hz	MAP PSD	Intercept	15793 (95% CI: -4383, 35969)	p=0.119
		SexMale	14332 (95% CI: -1417, 30080)	p=0.072
		SexMixed	5080 (95% CI: -9887, 20047)	p=0.489
		Age	-215 (95% CI: -618, 188)	p=0.281
		Number	280 (95% CI: -701, 1261)	p=0.560
	MCA PSD	Intercept	10196 (95% CI: 1357, 19036)	p=0.026
		SexMale	5229 (95% CI: -1670, 12129)	p=0.130
		SexMixed	5058 (95% CI: -1499, 11615)	p=0.124
		Age	***-240 (95% CI: -417, -64)***	***p=0.010***
		Number	246 (95% CI: -184, 676)	p=0.248
	MCA Coherence	Intercept	0.91 (95% CI: 0.86, 0.96)	p=0.001
		SexMale	***0.05 (95% CI: 0.01, 0.10)***	***p=0.027***
		SexMixed	0.03 (95% CI: -0.02, 0.08)	p=0.193
		Age	0.00 (95% CI: -0.00, 0.00)	p=0.419
		Number	0.00 (95% CI: -0.00, 0.00)	p=0.629
	MCA Phase	Intercept	0.89 (95% CI: 0.62, 1.17)	p=0.001
		SexMale	-0.07 (95% CI: -0.30, 0.16)	p=0.541
		SexMixed	-0.23 (95% CI: -0.47, 0.02)	p=0.066
		Age	0.00 (95% CI: -0.01, 0.01)	p=0.887
		Number	-0.00 (95% CI: -0.00, 0.00)	p=0.970
	MCA Gain	Intercept	0.85 (95% CI: 0.67, 1.04)	p=0.001
		SexMale	***-0.14 (95% CI: -0.28, 0.00)***	***p=0.050***
		SexMixed	-0.06 (95% CI: -0.19, 0.08)	p=0.403
		Age	-0.00 (95% CI: -0.01, 0.00)	p=0.386
		Number	-0.00 (95% CI: -0.00, 0.00)	p=0.633
	MCA nGain	Intercept	1.14 (95% CI: 1.01, 1.27)	p=0.001
		SexMale	0.04 (95% CI: -0.06, 0.13)	p=0.427
		SexMixed	-0.04 (95% CI: -0.14, 0.07)	p=0.452
		Age	-0.00 (95% CI: -0.01, 0.00)	p=0.194
		Number	0.00 (95% CI: -0.00, 0.00)	p=0.071
0.10 Hz	MAP PSD	Intercept	20248 (95% CI: 10609, 29888)	p=0.001
		SexMale	8759 (95% CI: -924, 18443)	p=0.074
		SexMixed	3713 (95% CI: -5413, 12840)	p=0.409
		Age	***-262 (95% CI: -484, -40)***	***p=0.023***
		Number	-136 (95% CI: -299, 27)	p=0.098
	MCA PSD	Intercept	24494 (95% CI: 11936, 37051)	p=0.001
		SexMale	5422 (95% CI: -7193, 18036)	p=0.384
		SexMixed	4327 (95% CI: -7562, 16215)	p=0.460
		Age	***-401 (95% CI: -690, -112)***	***p=0.009***
		Number	-121 (95% CI: -334, 92)	p=0.252
	MCA Coherence	Intercept	0.96 (95% CI: 0.93, 0.99)	p=0.001
		SexMale	0.01 (95% CI: -0.01, 0.04)	p=0.315

	SexMixed	-0.01 (95% CI: -0.03, 0.02)	p=0.595
	Age	0.00 (95% CI: -0.00, 0.00)	p=0.232
	Number	0.00 (95% CI: -0.00, 0.00)	p=0.303
MCA Phase	Intercept	0.70 (95% CI: 0.43, 0.97)	p=0.001
	SexMale	-0.01 (95% CI: -0.25, 0.23)	p=0.917
	SexMixed	-0.07 (95% CI: -0.32, 0.18)	p=0.573
	Age	-0.01 (95% CI: -0.01, 0.00)	p=0.109
	Number	-0.00 (95% CI: -0.00, 0.00)	p=0.731
MCA Gain	Intercept	1.02 (95% CI: 0.84, 1.21)	p=0.001
	SexMale	-0.06 (95% CI: -0.22, 0.10)	p=0.464
	SexMixed	-0.02 (95% CI: -0.18, 0.15)	p=0.836
	Age	-0.00 (95% CI: -0.01, 0.00)	p=0.166
	Number	-0.00 (95% CI: -0.00, 0.00)	p=0.948
MCA nGain	Intercept	1.46 (95% CI: 1.26, 1.65)	p=0.001
	SexMale	0.11 (95% CI: -0.06, 0.28)	p=0.179
	SexMixed	0.07 (95% CI: -0.11, 0.25)	p=0.448
	Age	-0.00 (95% CI: -0.01, 0.00)	p=0.084
	Number	0.00 (95% CI: -0.00, 0.00)	p=0.225
